# A multicenter randomized, double-blind, placebo-controlled pilot study to assess the efficacy and safety of riociguat in systemic sclerosis-associated digital ulcers

**DOI:** 10.1186/s13075-019-1979-7

**Published:** 2019-09-03

**Authors:** Vivek Nagaraja, Cathie Spino, Erica Bush, Pei-Suen Tsou, Robyn T. Domsic, Robert Lafyatis, Tracy Frech, Jessica K. Gordon, Virginia D. Steen, Dinesh Khanna

**Affiliations:** 1 Division of Rheumatology/Department of Internal Medicine, University of Michigan Scleroderma Program, Suite 7C27, 300 North Ingalls Street, SPC 5422, Ann Arbor, MI 48109 USA; 2 Department of Biostatistics, School of Public Health, University of Michigan, Ann Arbor, MI USA; 3 Division of Rheumatology and Clinical Immunology, University of Pittsburgh Medical Center, Pittsburgh, PA USA; 4 Division of Rheumatology, University of Utah, Salt Lake City, UT USA; 5 Division of Rheumatology, Hospital of Special Surgery, New York, NY USA; 6 Division of Rheumatology, Georgetown University Medical Center, Washington, DC, USA

**Keywords:** Systemic sclerosis, Digital ulcers, Riociguat, Clinical trial

## Abstract

**Background:**

To determine the effect of riociguat, an oral, selective soluble guanylate cyclase stimulator, on the net digital ulcer (DU) burden in systemic sclerosis (SSc).

**Methods:**

Participants with SSc-related active or painful indeterminate DUs were recruited in a multicenter, double-blind, randomized, placebo-controlled, proof-of-concept trial. Eligible participants were required to have at least one visible, active ischemic DU or painful indeterminate DU at screening, located at or distal to the proximal interphalangeal joint and that developed or worsened within 8 weeks prior to screening. Participants were randomized 1:1 to placebo or riociguat in individualized doses (maximum of 2.5 mg three times daily) during an 8-week titration period, followed by an 8-week stable dosing period. This was followed by an optional 16-week open-label extension phase for participants with active DU/reoccurrence of DUs within 1 month of the end of the main treatment phase. The primary endpoint was the change from baseline to week 16 in net ulcer burden (NUB), analyzed using ANCOVA. Other endpoints included plasma biomarkers and proportion of participants with treatment-emergent adverse events (AEs).

**Results:**

Seventeen participants (eight placebo, nine riociguat) were randomized at five centers. Six participants in each group transitioned to the open-label extension. Baseline characteristics were comparable between the treatment groups, except participants randomized to placebo were older and had longer disease duration (*p* < 0.05). At baseline, the mean (SD) NUB was 2.5 (2.0) in the placebo and 2.4 (1.4) in the riociguat. No significant treatment difference was observed in the change from baseline to 16 weeks in NUB (adjusted mean treatment difference − 0.24, 95% CI (− 1.46, 0.99), *p* = 0.70). Four participants experienced five serious AE (four in riociguat and one in placebo); none was considered related to study medication. Statistically significant elevation of cGMP was observed at 16 weeks in the riociguat group (*p* = 0.05); no other biomarkers showed significant changes. In the open-label extension, participants in the riociguat-riociguat arm had complete healing of their DUs.

**Conclusion:**

In participants with SSc-DU, treatment with riociguat did not reduce the number of DU net burden compared with placebo at 16 weeks. Open-label extension suggests that longer duration is needed to promote DU healing, which needs to be confirmed in a new trial.

**Trial registration:**

ClinicalTrials.gov, NCT02915835. Registered on September 27, 2016.

**Electronic supplementary material:**

The online version of this article (10.1186/s13075-019-1979-7) contains supplementary material, which is available to authorized users.

## Background

Systemic sclerosis (SSc) is an autoimmune disorder featuring chronic, fibrosing, autoimmune responses characterized by small vessel vasculopathy, autoantibody production, and fibroblast dysfunction leading to increased deposition of extracellular matrix [1]. Raynaud’s phenomenon (RP) is an almost universal manifestation of SSc, with 95% of all patients being affected, resulting in digital ulcers (DUs) in approximately 30% of the patients each year. DUs are associated with substantial morbidity (reduced quality of life, pain, disability, and disfigurement) that can escalate to gangrene and amputation in approximately 15% of patients [2, 3]. There are no drugs approved in the USA for the treatment of DUs. Treatments that have shown potential include calcium channel blockers, prostacyclin analogs, and endothelin receptor antagonists. Bosentan, a dual endothelin receptor antagonist, is approved in Europe to reduce the number of new DUs in patients with SSc. Trials and case series show beneficial efficacy of phosphodiesterase 5 (PDE5) inhibitors in healing of SSc-DUs, and this finding is supported by a meta-analysis [4].

Riociguat is the first in class of a new group of compounds, soluble guanylate cyclase (sGC) stimulators. Riociguat directly stimulates sGC, thereby increasing the levels of the signaling molecule cGMP. The cGMP molecule plays a pivotal role in regulating cellular processes, such as vascular tone, proliferation, fibrosis, and inflammation. Riociguat has a dual mode of action, directly stimulating sGC independent of nitric oxide (NO) and increasing the sensitivity of sGC to NO [5, 6]. Riociguat is approved for the treatment of two forms of pulmonary hypertension, pulmonary arterial hypertension (PAH), and chronic thromboembolic pulmonary hypertension [7–9]. In pre-clinical studies, riociguat has been shown to have vasodilatory, anti-proliferative, vascular remodeling, anti-fibrotic, and anti-inflammatory properties [10–15]. A recent single-dose, crossover trial showed that riociguat was well tolerated in patients with RP and resulted in improved digital blood flow in some patient subsets, with high inter-individual variability [16]. In an exploratory analysis of a phase 2 trial of riociguat in patients with early diffuse cutaneous SSc, there was a numerical tendency toward a reduction of RP symptoms and attack frequency with riociguat treatment compared with placebo [17].

The present proof-of-concept trial was designed to assess the efficacy and safety of 16 weeks of treatment with riociguat in a randomized, placebo-controlled clinical trial in patients with SSc-associated DUs followed by an optional open-label extension for additional 16 weeks.

## Methods

### Study design

**Fig. 1:**
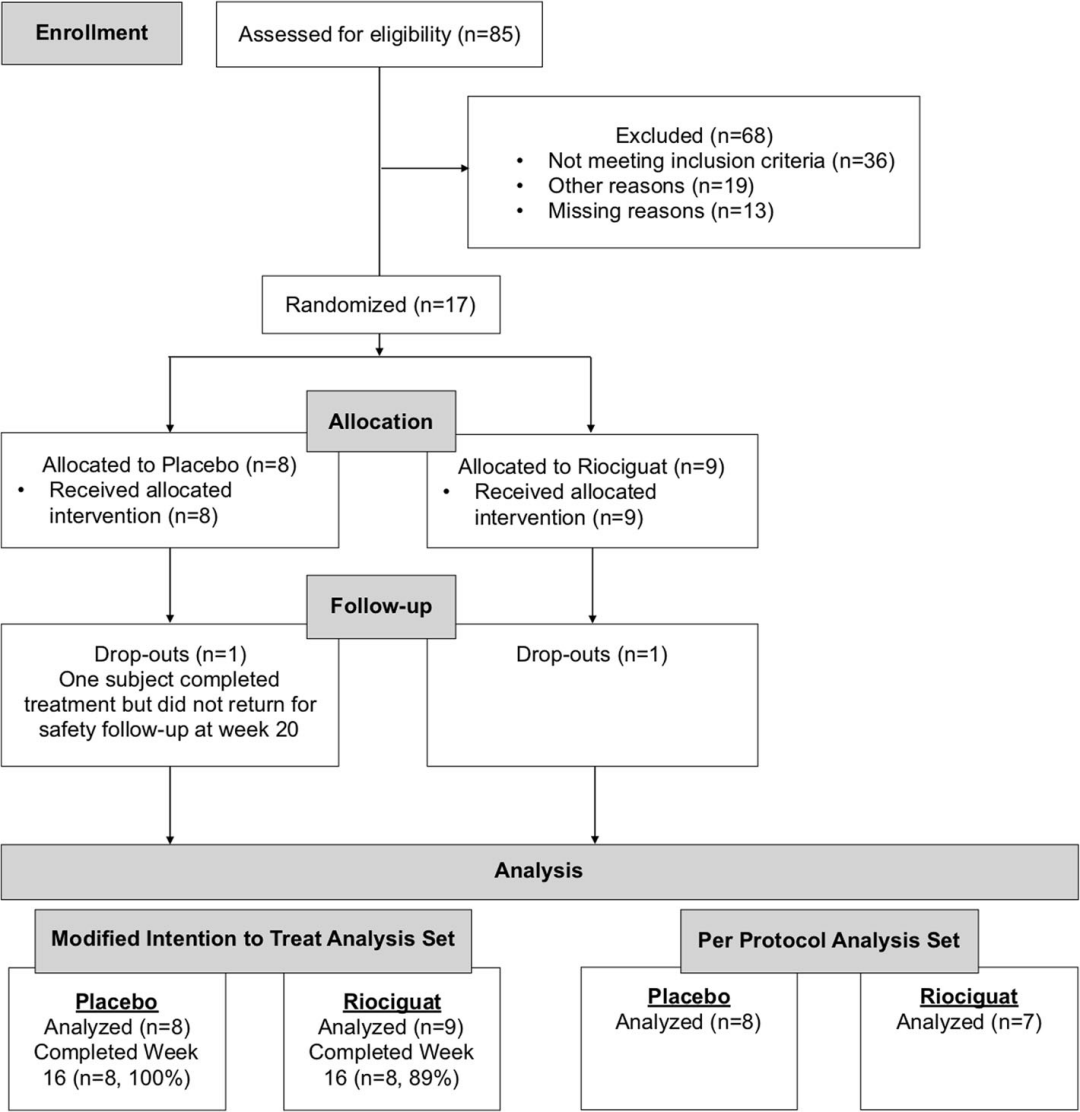
Consort diagram

This was an investigator-initiated, multicenter, double-blind, randomized, placebo-controlled, parallel group, proof-of-concept study comprising a 16-week treatment period (8-week titration, 8-week maintenance) followed by 16-week open-label extension phase for participants with active digital ulcer or reoccurrence of DUs within 1 month of the end of the main treatment phase (ClinicalTrials.gov Identifier: NCT02915835). Participants without an ischemic active or painful indeterminate DU after completion of the treatment period had a safety follow-up visit 4-weeks post-treatment. The study was conducted at five scleroderma centers in the USA. The Sponsor (Dinesh Khanna, MD) received an IND exemption from the Food and Drug Administration. Each site’s institutional review board or ethics committee approved the protocol before the study commenced. The study was conducted in accordance with the principles of the Declaration of Helsinki. After providing written informed consent, participants entered a screening phase (lasting up to 2 weeks), where their eligibility was evaluated (Fig. 1). Participants completed a diary detailing the number and duration of Raynaud’s attacks per day for a period of at least seven consecutive days before the baseline visit.

### Randomization and masking

Eligible participants were randomized in a 1:1 ratio to either riociguat or matching placebo (provided by the Bayer). The Data Coordinating Center (DCC) at the University of Michigan prepared the randomization schedule, using permuted block randomization with size 2 for the first block and then the random block sizes of 2 and 4 thereafter (block sizes were known only by the DCC). A secure web-based randomization and drug dispensing application were built by the DCC that was used by coordinators to obtain the randomization number and medication bottle numbers. This information was printed for the subject binder and used to prepare an investigator-signed prescription for the site pharmacist. The study staff (including the research pharmacists and assessors of DU) and participants were blinded to the treatment assigned.

**Fig. 2:**
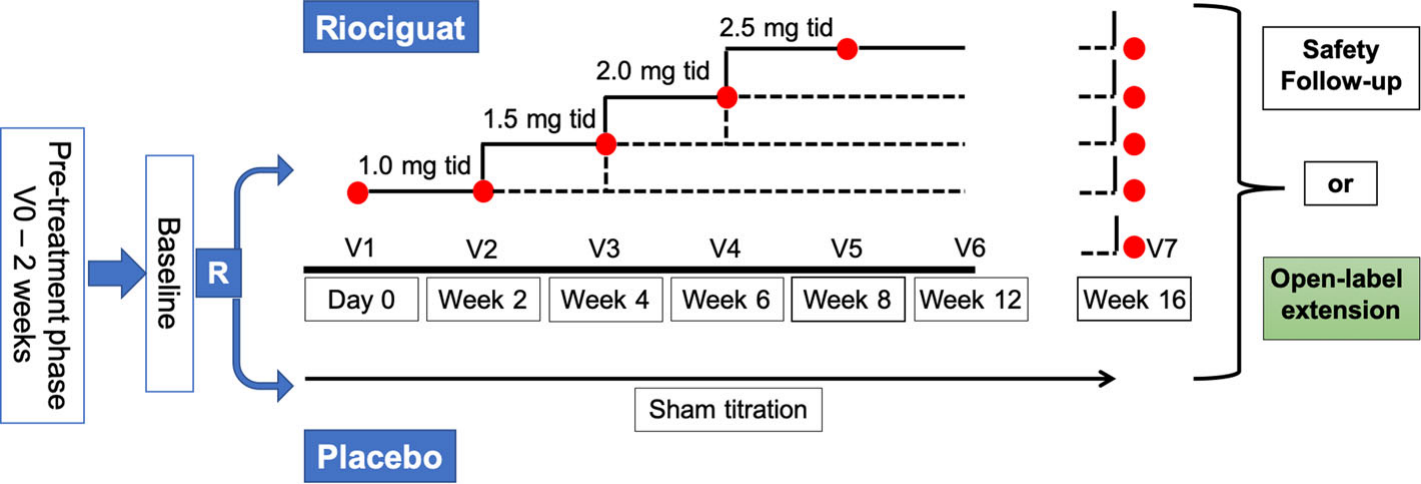
Study design

In the titration phase of 8 weeks, participants started at a dose of 1.0 mg three times a day (TID). The individual study medication dose for the next titration step was determined every 2 weeks according to the patient’s well-being and the peripheral systolic blood pressure measured at trough before intake of the morning dose according to the individual dose titration scheme (Fig. 2). The dose was increased by 0.5 mg increments no sooner than 2 weeks (± 4 days) apart to 1.5 mg, 2 mg, and 2.5 mg TID, resulting in a potential maximum total daily dose of 7.5 mg (2.5 mg TID). Participants were maintained on a lower dose if higher doses were not tolerated (minimum dosage of 0.5 mg TID, total daily dose 1.5 mg). While it was possible for a participant to be up-titrated and then down-titrated during this phase, once a participant had been down-titrated, they remained at that dose, and dose escalation was not implemented again. The established individual dose was then taken as the “optimal individual dose” to be administered for the remaining duration of study. To maintain the blinding of the treatment arms, participants randomized to the placebo group underwent sham titration from visit 1 onwards during the dose-titration period.

At week 16, all participants who agreed to continue in the open-label extension were assigned to treatment with riociguat. During the first 8 weeks of the open-label extension phase, participants previously on placebo were up-titrated on riociguat as per the individual titration algorithm described in the double-blind treatment phase. Participants randomized to riociguat in the double-blind study treatment phase also underwent a titration phase in the open-label extension.

### Patient selection

Eligible patients were ≥ 18 years old with a diagnosis of SSc according to the American College of Rheumatology (ACR)/European League Against Rheumatism (EULAR) 2013 classification criteria (total score of ≥ 9) [18]. Participants were required to have at least one visible, active ischemic DU or painful indeterminate DU at screening, located at or distal to the proximal interphalangeal joint, and that developed or worsened within 8 weeks prior to screening. An active DU was defined as a full-thickness skin lesion, > 3 mm in maximal diameter, with loss of epithelization, epidermis, and dermis. An indeterminate ulcer was defined as one where denudation could not be visualized and there were no other clinical features of activity. We excluded DUs due to calcinosis (based on baseline hand X-ray in every participant), paronychia, and osteomyelitis. We also excluded fissures, pitting scars, hyperkeratotic lesions, and DUs over the metacarpophalangeal joints (MCPs) or elbows. Photographs of the cardinal DU were taken and confirmed to meet the study inclusion after the review of the photograph by DK. We provided a written standardized wound care for each participant. Females of reproductive potential (FRP) were required to have a negative urine pregnancy test. During treatment, FRP were required to obtain monthly urine pregnancy tests during treatment and 1 month after treatment discontinuation. Oral corticosteroids (≤ 10 mg/day of prednisone or equivalent), non-steroidal anti-inflammatory drugs, angiotensin receptor blockers, angiotensin-converting enzyme inhibitors, and calcium channel blockers were permitted if the participant was on a stable dose for ≥ 2 weeks prior to and including the baseline visit. Participants with sitting systolic blood pressure < 95 mmHg, sitting heart rate < 50 beats/min, left ventricular ejection fraction < 40%, or anemia with hemoglobin < 9.0 g/dl, PAH requiring pharmacologic therapy, significant pulmonary disease [FVC ≤ 50% of predicted, or DLCO (uncorrected for hemoglobin) ≤ 40% of predicted done as part of clinical care], active state of hemoptysis or pulmonary hemorrhage, or any history of bronchial artery embolization or massive hemoptysis within 3 months prior to screening were excluded in the trial. We excluded participants with concomitant use of nitrates or NO donors (such as amyl nitrate) in any form, PDE5 inhibitors (such as sildenafil, tadalafil, or vardenafil due to the risk of hypotension and both target the nitric oxide pathway), and endothelin receptor antagonists. If the participant was on PDE5 inhibitors, a wash out of 3 days was required for sildenafil and 7 days for tadalafil or vardenafil prior to the baseline visit. Patients who were actively smoking at the time of consent were excluded due to the impact of nicotine on pharmacokinetics of riociguat; a quit date of 2 weeks prior to screening was acceptable. Complete inclusion/exclusion criteria are provided in Additional file 1.

### Study outcome measures

The primary efficacy endpoint was the change from baseline to week 16 (end of study treatment phase) in net ulcer burden (NUB). NUB is defined as the total number of active and painful indeterminate DUs at an assessment, and the change from baseline in NUB reflects both new DUs as well as healed DUs. For example, if a participant had two active DUs and one painful indeterminate DU at baseline (NUB = 3) and at week 4 had one of the active DUs healed, no change in the other DUs, and development of a two new active DUs (NUB = 4), the change would be one. NUB captures the overall impact of DUs on hand function and quality of life and has been used in a previous trial [2]. Other pre-specified secondary efficacy endpoints included the proportion of participants with the following at week 16: healing of the cardinal DU (healing was defined by re-epithelialization with loss of pain and exudate); healing of all baseline DUs; no DUs; development of new active and indeterminate DUs; development or healing of ulcers over DIP, PIP, MCPs, and elbows; time to healing of the cardinal DU and all baseline DUs; development of new (active or indeterminate) DUs; improvement of RP based on Raynaud’s Condition Score (RCS); number and duration of Raynaud’s attacks per day; patient and physician assessment of RP; symptoms during RP attack (pain, numbness, and tingling); patient’s and physician’s global assessment on a Likert scale; health-related quality of life as measured using Patient-Reported Outcomes Measurement Information System (PROMIS)-29; physical function [as assessed by Health Assessment Questionnaire Disability Index (HAQ-DI); and Hand Disability in Systemic Sclerosis-DU (HDISS-DU)]; visual analog scales from Scleroderma Health Assessment Questionnaire (SHAQ) assessing burden of digital ulcers, Raynaud’s disease, gastrointestinal involvement, breathing, and overall disease; and documentation of digital ischemia requiring intravenous prostacyclin or digital gangrene or amputation.

### Biomarker measurement

To examine the effect of riociguat on biomarkers, patient plasma was collected at baseline and week 16. Plasma from age- and sex-matched healthy controls was also obtained. The biomarkers were measured by enzyme-linked immunosorbent assays (ELISA) and included total vascular endothelial growth factor (VEGF, R&D Systems), tissue plasminogen activator (tPA, Abcam), soluble E-selectin (sE-selectin, R&D Systems), basic fibroblast growth factor (bFGF, R&D Systems), vascular cell adhesion molecule 1 (VCAM-1, RayBiotech), soluble intracellular adhesion molecule 1 (sICAM-1, RayBiotech), N-terminal propeptide of type I collagen (PINP, MyBioSource), matrix metalloproteinase 12 (MMP12, RayBiotech), CXCL4 (R&D Systems), cyclic guanylyl cyclase (cGMP, Cayman Chemical), endostatin (R&D Systems), and soluble fms-like tyrosine kinase 1 (sFLT1, R&D Systems).

### Sample size

The planned sample size of 20 participants was based primarily on practical considerations, not on power to achieve a pre-determined treatment difference. The goal of this pilot study was to obtain preliminary estimates of the magnitude of treatment effects of key efficacy and safety parameters. There are no published data on the minimal clinically important difference (MCID) for the change from baseline in NUB at week 16 (our primary efficacy endpoint), and our study did not seek to establish the MCID. Rather, we designed this pilot study with a placebo control arm and randomization to reduce bias in the estimation of NUB, so that we could also obtain a preliminary estimate of treatment differences. As expected with a pilot study, only large treatment differences can be detected. With the proposed sample of 10 riociguat and 10 placebo participants, we calculated the effect size (mean treatment difference divided by standard deviation) for the primary efficacy endpoint to be 1.253 with 80% power and a two-sided type I error of 5% based on a two-sample *t* test. If a statistically significant would be observed in our small study, it would need to be replicated in a larger confirmatory study.

### Statistical analysis

Continuous variables were summarized using means, standard deviations (SD), median, interquartile range (IQR), and range, and qualitative variables were summarized using counts and percentages. Mean (SD) is reported, unless otherwise noted. The primary and secondary efficacy endpoints were analyzed using the modified intention-to-treat population (MITT), defined as all participants randomized, receiving at least one dose of treatment, and having at least one post-baseline efficacy assessment. As a sensitivity analysis, the primary endpoint was also analyzed using the per-protocol set, defined as the MITT population who did not have a major protocol violation. For the primary analysis, changes in NUB were compared in the two treatment groups using an ANCOVA model, with terms for treatment group and baseline NUB value. Distributional assumptions were assessed. Analysis for secondary outcome measures that are continuous was performed using a similar approach as that for the primary endpoint. For analyses of discrete secondary outcomes measures, we used Fisher’s exact tests. Poisson regression was used for outcome measures that were counts (e.g., number of AEs) and log-rank tests, and Kaplan-Meier plots were used for time-to-event outcomes. Plasma biomarker changes from baseline (week 0) to week 16 were analyzed using the ANCOVA model. Safety analyses were performed on the safety analysis set which included all participants who were randomized and received at least one dose of the study drug. Statistical tests were conducted at the 0.05 significance level (with no adjustments for multiplicity) using two-tailed tests. Statistical analyses were performed using SAS version 9 or higher. Further details on the statistical analysis can be found in Additional file 2.

## Results

### Participant disposition and baseline characteristics

Twenty-five participants were screened across 5 centers in the USA between January 2017 and May 2018. Seventeen participants were randomized to either placebo (*n* = 8) or riociguat (*n* = 9), of which all 17 (88%) participants formed the MITT and safety analysis sets (Fig. 2), and the trial was stopped in May 2018 due to warm weather and poor recruitment and since this was a proof-of-concept study. Fifteen participants were included in the per-protocol analysis set (8 in placebo and 7 in riociguat). One participant withdrew in each group. One riociguat participant was withdrawn by the investigator due to worsening DU and RP, and the placebo participant completed treatment but did not return for the safety follow-up at week 20. Mean compliance with study drug in the treatment phase was 92%, 96% with placebo, and 88% with riociguat. Six subjects in each group progressed to the open-label extension phase with a 100% compliance till the end of the phase.

Baseline demographics and clinical characteristics were largely similar between the two groups, but the participants in the placebo group had longer duration (in years) of SSc diagnosis (mean [SD] 15.0 [8.2] years vs 6.2 [5.8] years) and of non-Raynaud’s symptoms (17.5 [11.2] years vs 7.1 [6.0] years). (Table 1). At baseline, the mean [SD] NUB was 2.5 (2.0) in the placebo group and 2.4 (1.4) in the riociguat group. Participants randomized to riociguat had numerically worse RP—higher RCS, more frequent and longer Raynaud’s attacks, more intense symptoms associated with RP (pain, numbness, and tingling), and higher S-HAQ scores for DUs indicating increased interferences of DUs with daily activities.

**Table 1:** Baseline demographic and clinical characteristics of all of the randomized patients

Characteristics	Double-blind phase			Open-label extension		
	PBO (*n* = 8)	RIO (*n* = 9)	All patients (*n* = 17)	PBO-RIO (*n* = 6)	RIO-RIO (*n* = 6)	All patients (*n* = 12)
Age in years, mean (SD)	61 (17)	43 (14)	51 (18)	61 (20)	44 (14)	52 (19)
Gender, *n* (%)						
Male	3 (38)	1 (11)	4 (24)	3 (50)	0 (0)	3 (25)
Female	5 (63)	8 (89)	13 (76)	3 (50)	6 (100)	9 (75)
Race, *n* (%)						
Caucasian	7 (88)	6 (67)	13 (76)	5 (83)	5 (83)	10 (83)
African-American	1 (13)	2 (22)	3 (18)	1 (17)	0 (0)	1 (8)
Others	0 (0)	1 (11)	1 (6)	0 (0)	1 (17)	1 (8)
SSc subset, *n* (%)						
Limited cutaneous SSc	4 (50)	5 (56)	9 (53)	2 (33)	4 (67)	6 (50)
Diffuse cutaneous SSc	4 (50)	4 (44)	8 (47)	4 (67)	2 (33)	6 (50)
Time since SSc diagnosis, in years, mean (SD)^†^	15.0 (8.2)	6.2 (5.8)	10.4 (8.2)	14.3 (8.0)	5.2 (6.0)	9.7 (8.2)
Time since first non-RP symptom, in years, mean (SD)^††^	17.5 (11.2)	7.1 (6.0)	12 (10.1)	16.9 (12.1)	5.7 (5.8)	11.3 (10.8)
Time since first RP symptom, in years, mean (SD)^††^	14.5 (7.9)	7.5 (6.6)	11 (7.9)	13.2 (6.7)	6.9 (6.9)	10.1 (7.3)
Time since first DU,, in years^†^	8.0 (6.8)	5.4 (4.6)	6.7 (5.7)	9.8 (7.0)	3.5 (3.1)	6.7 (6.1)
Number of DU, mean (SD)^††^	2.5 (1.7)	2.7 (1.8)	2.6 (1.7)	2.7 (1.8)	1.7 (0.8)	2.2 (1.5)
Number of active DU	1.4 (1.1)	1.1 (1.0)	1.2 (1.0)	1.2 (1.0)	0.5 (0.5)	0.8 (0.8)
Number of indeterminate DU	1.1 (1.4)	1.6 (1.3)	1.3 (1.3)	1.5 (1.4)	1.2 (1.2)	1.3 (1.2)
Net ulcer burden	2.5 (2.0)	2.4 (1.4)	2.5 (1.7)	2.7 (2.3)	1.7 (0.8)	2.2 (1.7)
Characteristics of Raynaud’s attacks						
Raynaud’s Condition Score (0–10 Likert scale), mean (SD)^†^	3.4 (2.2)	5.4 (1.6)	4.5 (2.1)	4.0 (1.8)	5.1 (1.9)	4.6 (1.8)
Number of Raynaud’s attacks per day, mean (SD)^†^	2.2 (1.7)	4.3 (1.7)	3.3 (2.0)	2.4 (1.8)	3.6 (1.4)	3.0 (1.7)
Pain during a RP attack (0–100 VAS scale), mean (SD)^†^	37.2 (24.6)	54.9 (13.1)	46.6 (20.7)	41.7 (23.6)	53.4 (14.2)	47.5 (19.5)
Numbness during a RP attack (0–100 VAS scale), mean (SD)^†^	32.0 (30.2)	40.5 (15.9)	36.5 (23.2)	35.6 (31.4)	43.7 (16.3)	39.7 (24.2)
Tingling during a RP attack (0–100 VAS scale), mean (SD)^†^	26.9 (16.1)	34.8 (16.6)	31.1 (16.3)	29.7 (15.7)	37.7 (15.9)	33.7 (15.6)
Duration of RP attacks, in minutes, mean (SD)^†^	47.9 (51.6)	101.4 (117.3)	76.4 (93.8)	55.0 (52.6)	112.5 (136.4)	83.7 (103.0)
Patient assessment of RP (0–10 Likert scale)						
Severity of RP, mean (SD)^†^	4.2 (2.7)	7.1 (1.4)	5.8 (2.5)	4.8 (2.5)	7.0 (1.7)	5.9 (2.3)
Severity of DU, mean (SD)^†^	6.7 (1.9)	8.0 (1.5)	7.4 (1.8)	6.3 (1.6)	8.3 (1.2)	7.3 (1.7)
SHAQ: DUs interfere with daily activities in past week (theoretical range, 0–150), median (IQR)	55 (36–102)	116 (98–125)	98 (62–124)	64.3 (33.8)	107.2 (21.3)	85.7 (35.0)
Physician assessment of RP (0–10 Likert scale)						
Severity of RP, mean (SD)^†^	5.4 (3.0)	6.1 (1.0)	5.8 (2.1)	6.2 (2.2)	6.0 (1.3)	6.1 (1.7)
Severity of DU, mean (SD)^†^	6.3 (2.6)	6.4 (1.9)	6.4 (2.1)	5.6 (2.5)	6.7 (2.5)	6.2 (2.3)
SSc-related antibodies, *n* (%)^‡^						
Anti-centromere B	3 (38)	3 (33)	6 (35)	2 (33)	3 (50)	5 (42)
Anti-topoisomerase I	3 (38)	2 (22)	5 (29)	3 (50)	0 (0)	3 (25)
Anti-RNA polymerase III	0 (0)	1 (11)	1 (6)	0 (0)	1 (17)	1 (8)
Not done	2 (25)	3 (33)	5 (29)	1 (17)	2 (33)	3 (25)
Baseline use of medications, *n* (%)						
Vasodilators	1 (13)	1 (11)	2 (12)	0 (0)	1 (17)	1 (8)
Prednisone	1 (13)	0 (0)	1 (6)	1 (17)	0 (0)	1 (8)
Immunosuppressive	0 (0)	0 (0)	0 (0)	0 (0)	0 (0)	0 (0)

Mean (SD), unless otherwise mentioned

*SSc* systemic sclerosis, *DU* digital ulcer, *RP* Raynaud’s phenomenon, *SHAQ* Scleroderma Health Assessment Questionnaire, *IQR* interquartile range, *PAH* pulmonary arterial hypertension, *ILD* interstitial lung disease, *CCB* calcium channel blocker

^†^Calculated from date of screening or at the screening

^‡^Classes not mutually exclusive

### Dosing and exposure

The median duration of exposure to study drug was 112 days in each treatment group. At the end of the 8-week titration phase, all eight participants in the placebo group reached the 2.5 mg TID dosing level whereas in the riociguat group, three reached 1.5 mg TID, one reached 2.0 mg TID, and four reached the 2.5 mg TID dosing levels.

### Primary efficacy endpoint

**Fig. 3:**
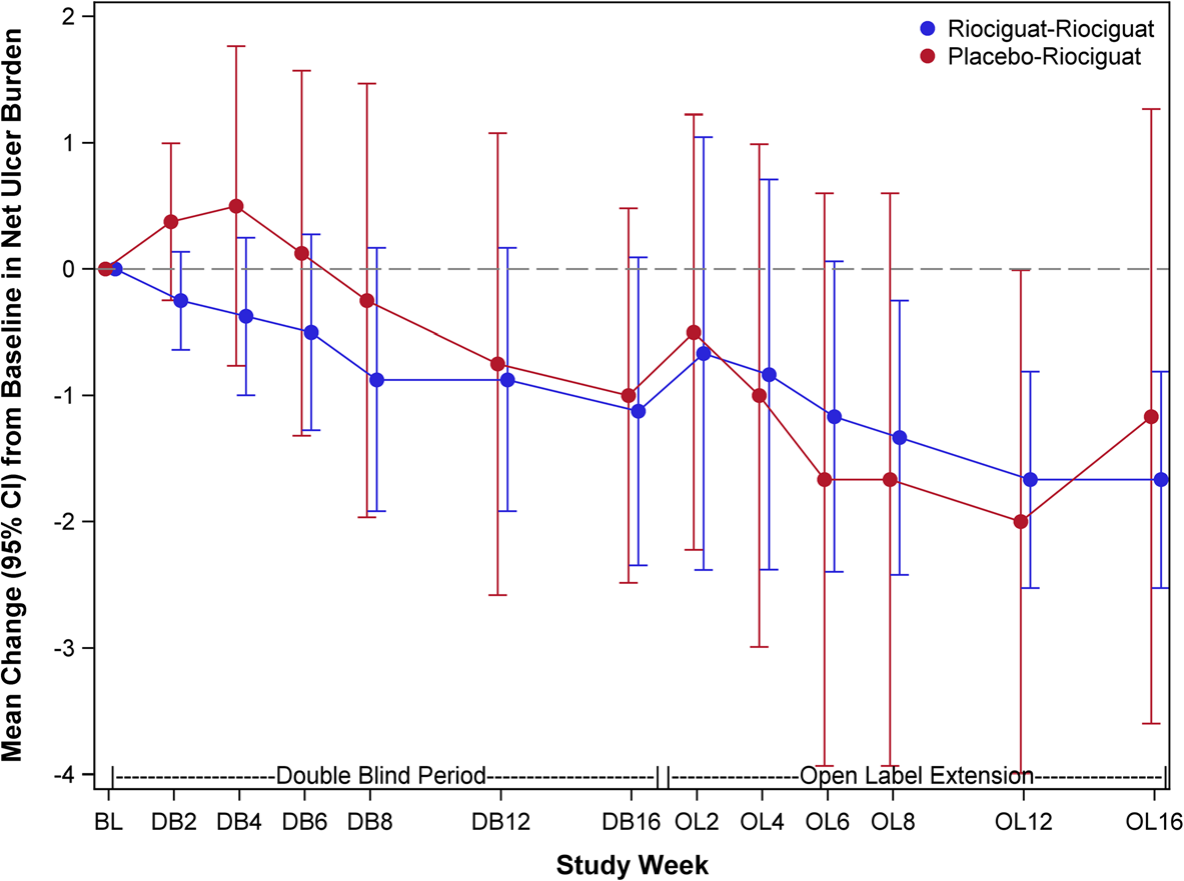
Mean trend over time: change in net ulcer burden. Digital ulcer net burden is defined as the total number of “active” and indeterminate digital ulcers at an assessment. LS, least squares; SE, standard error; CI, confidence interval. Estimates are from an ANCOVA model with terms for the treatment group and baseline digital net ulcer burden. Modified intent-to-treat population is defined as all participants randomized, receiving at least one dose of treatment, and having at least one post-baseline efficacy assessment

There was no statistically significant difference between riociguat and placebo in the change in NUB (Fig. 3). The least square (LS) mean change from baseline to 16 weeks in NUB was − 1.22 in the riociguat group and − 0.98 in the placebo group (negative score denotes improvement, treatment difference − 0.24, 95% CI − 1.46 to 0.99; *p* = 0.70; Table 2). Sensitivity analyses (using the per-protocol analysis set or controlling for age in the MITT analysis set) also showed statistically non-significant treatment differences (LS mean treatment difference − 0.08, 95% CI − 1.62 to 1.46; *p* = 0.92 in ANCOVA adjusting for baseline NUB and age).

**Table 2:** Changes from baseline to week 16 in primary and secondary efficacy endpoints

	Placebo (*N* = 8)	Riociguat (*N* = 7)	Treatment difference (95% CI)	*p* value
Net ulcer burden, LS mean^†^*	− 0.98	− 1.22	− 0.24 (− 1.46 to 0.99)	0.706
Patient global assessment for overall disease, LS mean^†^	− 1.19	0.31	1.50 (− 1.30 to 4.30)	0.27
Patient assessment, LS mean^†^				
Severity of RP	− 1.41	− 3.47	− 2.06 (− 4.63 to 0.51)	0.11
Severity of DU	− 4.00	− 4.63	− 0.63 (− 3.68 to 2.41)	0.66
Pain during RP attack (0–100)	− 7.01	− 0.30	6.71 (− 14.01 to 27.43)	0.49
Numbness during RP attack (0–100)	− 15.44	− 19.73	− 4.28 (− 33.44 to 24.87)	0.75
Tingling during RP attack (0–100)	− 7.49	1.18	8.67 (− 13.75 to 31.09)	0.41
Raynaud’s Condition Score, LS mean^†^	− 0.82	− 1.15	− 0.33 (− 2.60 to 1.94)	0.76
Number of Raynaud’s attacks per day, LS mean^†^	− 0.96	− 1.24	− 0.28 (− 1.36 to 0.79)	0.57
Duration of Raynaud’s attacks (minutes), LS mean^†^	150.3	− 44.8	− 195.1 (− 683.7 to 293.5)	0.40
Physician global assessment for overall disease, LS mean^†^	− 0.66	− 1.17	− 0.51 (− 2.27 to 1.25)	0.54
Physician assessment of the severity of RP, LS mean^†^	− 1.86	− 3.00	− 1.15 (− 3.51 to 1.22)	0.32
Physician assessment of severity of DU, LS mean^†^	− 3.81	− 3.54	0.27 (− 2.55 to 3.10)	0.84
SHAQ-DI (VAS range 0–150), LS mean^†^				
VAS overall disease	− 35.74	− 50.35	− 14.60 (− 45.48 to 16.27)	0.32
VAS burden of DU	− 53.47	− 43.09	10.38 (− 58.03 to 78.79)	0.75
VAS Raynaud’s	− 25.40	− 23.78	1.62 (− 54.56 to 57.80)	0.95
VAS GI	− 7.39	16.72	24.11 (− 25.22 to 73.44)	0.31
VAS breathing	− 12.69	8.35	21.04 (− 8.05 to 50.13)	0.14
PROMIS-29, LS mean^†^				
Anxiety	− 1.50	−3.11	− 1.60 (− 9.77 to 6.56)	0.68
Depression	− 0.25	− 3.35	− 3.10 (− 8.65 to 2.45)	0.25
Fatigue	0.46	0.64	0.19 (− 6.26 to 6.63)	0.95
Physical function	− 2.24	− 2.46	− 0.22 (− 3.96 to 3.53)	0.90
Sleep disturbance	0.94	− 0.47	− 1.41 (− 4.76 to 1.95)	0.38
Pain interference	− 3.06	− 3.69	− 0.62 (− 6.54 to 5.30)	0.82
Pain intensity	− 1.63	− 2.74	− 1.11 (− 3.59 to 1.37)	0.35
Ability to participate in social activities	− 0.46	− 1.68	− 1.22 (− 4.80 to 2.36)	0.47
HAQ-DI, LS mean^†^				
Overall	− 0.06	− 0.01	0.04 (− 0.44 to 0.53)	0.84
Dressing and grooming	0.05	− 0.16	− 0.21 (− 0.96 to 0.54)	0.55
Hygiene	− 0.27	− 0.39	− 0.11 (− 1.00 to 0.78)	0.79
Arising	0.02	0.36	0.34 (− 0.41 to 1.09)	0.34
Reach	0.01	0.37	0.36 (− 0.46 to 1.19)	0.36
Eating	0.03	− 0.53	− 0.56 (− 1.08 to − 0.03)	*0.04*
Grip	− 0.18	0.16	0.34 (− 0.37 to 1.04)	0.31
Walking	− 0.12	0.35	0.47 (− 0.18 to 1.12)	0.14
Common daily activities	0.04	− 0.29	− 0.33 (− 1.17 to 0.51)	0.40
HDISS-DU, LS mean^†^	− 0.32	− 0.47	− 0.14 (− 1.75 to 1.46)	0.85

*LS mean* least squares mean from an ANCOVA model with treatment and baseline value as covariates, *SE* standard error, *DU* digital ulcer, *RP* Raynaud’s phenomenon, *SHAQ-DI* Scleroderma Health Assessment Questionnaire Disability Index, *VAS* visual analog scale, *GI* gastrointestinal, *PROMIS* Patient-Reported Outcomes Measures Information System, *HAQ-DI* Health Assessment Questionnaire Disability Index, *HDISS-DU* Hand Disability in Systemic Sclerosis Digital Ulcer

*DU net burden is defined as the total number of active and painful indeterminate digital ulcers at an assessment; †Estimates and p-values are from an ANCOVA model, adjusting for baseline values of the outcome

### Secondary endpoints

There were no statistically significant treatment differences in secondary efficacy endpoints, except for the eating component of HAQ-DI (Table 2). There were no statistically significant differences between the two treatments in RCS or frequency of RP attacks, patient self-assessments, PROMIS-29 measures, and overall HAQ-DI score. However, we noted greater numerical improvements in riociguat, relative to placebo, in some outcome measures but were not significant (Table 2). Participants in the riociguat group reported decreased pain due to RP (LS mean treatment difference 6.71, 95% CI − 14.01 to 27.43; *p* = 0.49) and tingling due to RP (LS mean treatment difference 8.67, 95% CI − 13.75 to 31.09; *p* = 0.41) during RP attacks and decreased duration of RP attacks (LS mean treatment difference − 195.1 min, 95% CI − 683.7 to 293.5; *p* = 0.40) compared to the placebo group (both pain and tingling due to RP are measured on a 0–100 scale). In both treatment groups, three (38%) participants underwent healing of all baseline DUs by week 16, with a median time to healing of 16 weeks (placebo, 1.15 riociguat; *p* = 0.35). Healing of the cardinal DU by week 16 was seen in 6 (75%) placebo and four (50%) riociguat participants. The median time to healing of the cardinal DU was 16.0 weeks (IQR 0.14) in the placebo group and 16.6 weeks (IQR 1.15) in the riociguat group (*p* = 0.56). The percentage of participants without any DUs at the end of the study period was two (25%) in each of the treatment groups. Four (50%) and three (38%) of placebo and riociguat participants, respectively, developed new ulcers of the 16 weeks of treatment (*p* = 1.0).

### Biomarker data

There were statistical differences in the baseline values for the biomarkers between healthy controls and all patients for cGMP, sE-selection, and sICAM1 (*p* < 0.05, Additional file 3). No statistical differences were observed in patients in the placebo and riociguat arms at baseline except for MMP12; patients in the riociguat group had significantly higher MMP12 levels compared to the placebo group (*p* < 0.05, Additional file 3). Our ANCOVA analysis revealed that after 16 weeks of riociguat treatment, there were no significant changes in the biomarkers measured, except for cGMP, which was significantly elevated in the riociguat group, confirming target engagement (*p* = 0.046, Table 3).

**Table 3:** Plasma biomarker changes from baseline (week 0) to week 16. Data presented as Mean (SD)

Biomarkers	Adjusted Means (SE)^a^		
	Placebo (*n* = 8)	Riociguat (*n* = 9)	p-value
cGMP (nM)	41.22 (50.2)	198.5 (47.03)	**0.046**
CXCL4 (ng/ml)	-7.3 (105.9)	-190.7 (99.8)	0.23
sE-Selectin (ng/ml)	-2.5 (2.0)	-4.7 (1.9)	0.45
VEGF (pg/ml)	-34.7 (10.1)	-29.8 (9.5)	0.73
sFLT1 (pg/ml)	-218.0 (211.8)	-220.5 (183.2)	0.99
tPA (ng/ml)	-0.5 (0.5)	-1.2 (0.5)	0.32
bFGF (pg/ml)	-0.03 (0.29)	0.20 (0.28)	0.58
sICAM1 (ng/ml)	-39.2 (50.2)	70.5 (47.3)	0.14
VCAM1 (ng/ml)	-3.4 (11.0)	10.3 (10.4)	0.39
PINP (pg/ml)	-6.6 (11.0)	7.1 (10.3)	0.38
MMP12 (ng/ml)	0.12 (0.14)	-0.09 (0.13)	0.31
Endostatin (ng/ml)	-96.8 (132.2)	-299.0 (124.5)	0.29

^1^biomarker values analyzed on natural scale using ANCOVA, adjusting for baseline biomarker value

### Safety and tolerability

Four serious AEs (SAEs) in 3 (14%) riociguat participants were reported: non-Hodgkin lymphoma, non-ST elevation myocardial infarction, digital ischemia, and worsening digital ulcer that required hospitalization for intravenous prostacyclin. One (8%) placebo participant experienced an SAE: persistent digital ischemia in a toe that required hospitalization for intravenous prostacyclin. All SAEs were considered not related to the study treatment. There were no deaths during the study. Thirteen adverse events (AEs) were reported in 8 (100%) participants in the placebo group and 21 were reported in 9 (100%) participants in the riociguat group (Table 4). Most AEs were reported as mild or moderate according to the Common Terminology Criteria for Adverse Events 5.0 (CTCAE 5.0) severity grading system: 13 (100%) in the placebo group and 77% in the riociguat group. There was no osteomyelitis or AEs of special interest (clinically significant hypotension or hemoptysis).

**Table 4:** Summary of adverse events

	Double-blind phase		Open-label extension	
	PBO (*n* = 8)	RIO (*n* = 9)	PBO-RIO (*n* = 6)	RIO-RIO (*n* = 6)
Treatment emergent AEs/SAEs	13	21	35	19
Treatment emergent AEs, *n* (%)	12 (92)	18 (86)	29 (83)	17 (90)
Participants with AEs, *n* (%)	8 (100)	9 (100)	6 (100)	5 (83)
Treatment emergent SAEs, *n* (%)	1 (8)	4 (19)	6 (17)	2 (10)
Participants with SAEs, *n* (%)	1 (13)	3 (33)	3 (50)	1 (17)
Participants with AEs leading to study drug discontinuation, *n* (%)	0 (0)	1 (11)	2 (33)	0 (0)
Participants with SAEs leading to study drug discontinuation, *n* (%)	0 (0)	0 (0)	1 (17)	0 (0)3
AEs according to system organ class^‡^, number of events (%)				
Blood and lymphatic system disorders	0 (0)	3 (14)	1 (3)	0 (0)
Cardiac disorders	1 (8)	2 (10)	3 (9)	2 (11)
Gastrointestinal disorders	1 (8)	2 (10)	9 (26)	4 (21)
General disorders	1 (8)	0 (0)	3 (9)	2 (11)
Hepatobiliary disorders	0 (0)	1 (5)	0 (0)	0 (0)
Infections and infestations	1 (8)	2 (10)	6 (17)	2 (11)
Injury, poisoning, and procedural complications	1 (8)	0 (0)	0 (0)	0 (0)
Metabolism and nutrition disorders	0 (0)	0 (0)	0 (0)	1 (5)
Musculoskeletal and connective tissue disorders	1 (8)	3 (14)	1 (3)	4 (21)
Nervous system disorders	4 (31)	5 (24)	3 (9)	0 (0)
Renal and urinary disorders	0 (0)	0 (0)	6 (17)	0 (0)
Respiratory, thoracic and mediastinal disorders	0 (0)	0 (0)	1 (3)	3 (16)
Surgical and medical procedures	1 (8)	0 (0)	0 (0)	0 (0)
Vascular disorders	2 (15)	3 (14)	1 (3)	1 (5)
SAEs according to system organ class^‡^, number of events (%)				
Blood and lymphatic system disorders				
Lymphoma	0 (0)	1 (33)	0 (0)	0 (0)
Cardiac disorders				
NSTEMI	0 (0)	1 (33)	0 (0)	0 (0)
Gastrointestinal disorders				
Acute ileus	0 (0)	0 (0)	1 (17)	0 (0)
Omental adhesions of lower abdomen	0 (0)	0 (0)	1 (17)	0 (0)
Infections and infestations				
Aspiration pneumonia	0 (0)	0 (0)	1 (17)	0 (0)
Musculoskeletal and connective tissue disorders				
Acute right rib fractures with hemopneumothorax	0 (0)	0 (0)	0 (0)	1 (17)
Nervous system disorders				
Possible transient ischemic attack	0 (0)	0 (0)	1 (17)	0 (0)
Respiratory, thoracic, and mediastinal disorders				
Acute respiratory failure secondary to pneumonia	0 (0)	0 (0)	1 (17)	0 (0)
Vascular disorders				
Digital ischemia	1 (100)	1 (33)	0 (0)	0 (0)
DU	0 (0)	1 (33)	0 (0)	0 (0)
Deep vein thrombosis	0 (0)	0 (0)	1 (17)	0 (0)
Pulmonary embolism	0 (0)	0 (0)	0 (0)	1 (17)

Safety analysis set for the double-blind treatment period. Values are the number (%)

*AEs* adverse events, *SAEs* serious adverse events, *NSTEMI* non-ST elevation myocardial infarction, *DU* digital ulcer

^‡^According to the Medical Dictionary for Regulatory Activities, version 18.0

#### Open-label extension

There was an improvement in the NUB in both placebo-riociguat and riociguat-riociguat groups from baseline and from week 16 of the treatment phase (Fig. 3). In the riociguat-riociguat group, all the DUs healed by week 16 of the open-label extension. Numerical improvements were noted in almost all the secondary efficacy endpoints by week 16 of the open-label extension in both groups but numerically favor in the riociguat-riociguat group (Table 5). Six SAEs were reported in total. In the placebo-riociguat arm, SAEs included the following: acute ileus (participant #1), omental adhesion of lower abdomen, transient ischemic attack, aspiration pneumonia, and deep vein thrombosis (all in participant #2), and acute respiratory failure secondary to pneumonia (participant #3). In the riociguat-riociguat arm, SAEs included the following: pulmonary embolism and acute right rib fractures with hemopneumothorax (both in participant #1). All SAEs were considered not related to the study treatment. There were no deaths during the open-label extension phase study. Thirty-five AEs were reported in 6 (100%) participants in the placebo-riociguat group and 19 were reported in 5 (83%) participants in the riociguat-riociguat group (Table 4). Most AEs were reported as mild or moderate according to the CTCAE 5.0 severity grading system.

**Table 5:** Summary of changes in the efficacy measures at the end of open-label extension

	Placebo-riociguat (*N* = 6)			Riociguat-riociguat (*N* = 6)		
	Baseline*	Week 16 OLE	Change from baseline	Baseline*	Week 16 OLE	Change from baseline
Net ulcer burden	2.7 (2.2)	1.5 (1.1)	− 1.2 (2.3)	1.67 (0.8)	0 (0)	− 1.67 (0.8)
Patient global assessment for overall disease	4.3 (2.2)	3.3 (1.9)	− 1.0 (2.3)	4.2 (2.9)	3.5 (1.6)	− 0.7 (2.6)
Patient assessment						
Severity of RP	4.8 (2.5)	3 (1.9)	− 1.8 (2.3)	7.0 (1.7)	3.0 (2.7)	− 4.0 (3.3)
Severity of DU	6.3 (1.6)	2.8 (1.5)	− 3.5 (2.6)	8.3 (1.2)	1.0 (0.9)	− 7.3 (1.9)
Physician global assessment for overall disease	4.8 (1.8)	5.2 (1.7)	0.6 (2.9)	6 (1.7)	3.2 (1.7)	− 1.8 (2.7)
Physician assessment of severity of RP	6.2 (2.2)	4.7 (2.2)	− 1.4 (2.4)	1.3 (5.5)	3.0 (2.2)	− 3.0 (1.3)
Physician assessment of severity of DU	5.6 (2.5)	2.8 (2.1)	− 2.2 (2.2)	6.7 (2.2)	0.5 (0.8)	− 6.2 (2.1)
SHAQ-DI (VAS range 0–150)						
VAS overall disease	94.2 (20.1)	65.5 (24.1)	− 28.7 (26.9)	100.5 (24.4)	42.8 (25.8)	− 57.7 (32.7)
VAS burden of DU	64.3 (33.8)	39.2 (35.2)	− 25.2 (42.1)	107.2 (21.3)	10.0 (6.3)	− 97.2 (24.2)
VAS Raynaud’s	56.8 (36.7)	37.8 (39.7)	− 19.0 (47.5)	69.7 (38.3)	36.7 (40.2)	− 33.0 (46.5)
VAS GI	6.8 (7.5)	46.2 (47.8)	39.3 (47.7)	44.5 (29.9)	45.5 (33.6)	1.0 (28.6)
VAS breathing	18.5 (29.4)	20.5 (27.5)	2.0 (14.7)	40.3 (22.6)	33.7 (27.2)	− 6.7 (24.9)
PROMIS-29						
Anxiety	51.5 (9.8)	51.9 (9.5)	3.2 (9.4)	61.4 (14.3)	56.7 (9.4)	− 4.8 (11.4)
Depression	51.2 (8.8)	51.4 (8.7)	0.2 (4.2)	57.4 (7.1)	55.7 (8.3)	− 1.7 (7.8)
Fatigue	44.7 (8.7)	54.0 (3.8)	9.2 (6.4)	57.6 (6.7)	54.7 (7.0)	− 2.9 (9.5)
Physical function	35.3 (5.4)	33.8 (3.4)	− 1.5 (4.1)	34.7 (3.3)	32.0 (3.6)	− 2.7 (1.9)
Sleep disturbance	51.1 (5.1)	49.8 (4.3)	− 1.3 (7.9)	52.3 (2.0)	49.9 (1.9)	− 2.4 (3.2)
Pain interference	60.3 (3.4)	49.6 (9.0)	− 10.6 (8.3)	63.8 (6.1)	58.5 (3.1)	− 5.3 (7.5)
Pain intensity	6.5 (1.5)	3.7 (2.1)	− 2.8 (3.0)	8.7 (0.8)	5.0 (1.1)	− 3.7 (1.0)
Ability to participate in social activities	41.1 (3.0)	40.7 (5.0)	− 0.4 (7.2)	45.7 (7.5)	41.8 (3.6)	− 4.0 (5.7)
HAQ-DI	1.3 (0.7)	1.5 (0.5)	0.9 (0.5)	1.2 (0.6)	0.9 (0.5)	− 0.3 (0.5)
HDISS-DU	5.2 (1.7)	5.5 (1.8)	0.3 (1.0)	4.8 (2.3)	4.2 (1.9)	− 0.7 (2.0)

* Baseline data is at the start of double blind study. DU digital ulcer, *RP* Raynaud’s phenomenon, *SHAQ-DI* Scleroderma Health Assessment Questionnaire Disability Index, *VAS* visual analog scale, *GI* gastrointestinal, *PROMIS* Patient-Reported Outcomes Measures Information System, *HAQ-DI* Health Assessment Questionnaire Disability Index, *HDISS-DU* Hand Disability in Systemic Sclerosis Digital Ulcer

## Discussion

The proof-of-concept trial was designed to evaluate the effect of riociguat on NUB in patients with SSc-related DUs. We did not find any statistical or clinically meaningful differences in the NUB and other secondary outcome measures, but we showed target engagement, as exemplified by an increase in plasma cGMP in the riociguat group. With a longer duration of treatment with riociguat, complete healing of DUs was observed as noted in the open-label extension phase. The safety profile of riociguat was consistent with that observed previously in studies of patients with PAH, with no new safety events identified [9].

Few therapies are available for DUs in patients with SSc. In the recently published EULAR guidelines, intravenous iloprost and oral sildenafil are recommended for the treatment of SSc-related DU, and oral bosentan is recommended for the prevention of new DUs, especially in patients with multiple digital ulcers despite the use of CCBs, PDE5 inhibitors, or iloprost therapy [19]. In the USA, iloprost is currently unavailable, and neither sildenafil nor bosentan is approved by the Food and Drug Administration for the treatment of DUs. Hence, there is a clear need for therapeutic agents for the management of DUs.

We designed the pilot trial to explore the unmet need of a therapeutic agent to treat SSc-DU by utilizing the pleiotropic effect of riociguat on vascular remodeling and anti-fibrotic, anti-proliferative effect, and anti-inflammatory effects [10–15]. We did not find statistically significant improvements in the primary and secondary outcome measures, including patient-reported symptoms of RP and PROs. There are several reasons for this negative trial. First, the trial design excluded PDE5 inhibitors due to relative contraindication of PDE5 inhibitors and riociguat (both target nitric oxide pathway and risk of significant hypotension). The trial allowed background stable CCBs, ACE inhibitors, and anti-platelet therapies. PDE5 inhibitors have become the mainstay for the management of SSc-DU, especially those who do not respond or are intolerant to CCBs [19]. This likely led to the recruitment of a population with milder burden of digital disease—the mean number of active and painful intermediate DUs at baseline was 2.6. Second, the participants in the trial had longer disease duration (mean [SD] 12.0 [10.1] years) compared to the recently completed RISE-SSc trial—a phase 2b randomized controlled trial in participants with early dcSSc (disease duration of mean 9 months) where riociguat was associated with trends in improvement in RP at 14 weeks and DUs at 52 weeks [17]. Although the participants in the placebo group had longer disease duration (15 [8.2] years vs 6.2 [5.8] years), both groups of patients may have had chronic irreversible vasculopathy that was not amenable to oral therapy like riociguat. Third, the duration of riociguat treatment in the RESCUE study, especially with an 8-week titration to maximum tolerated dose, may be suboptimal for healing of DUs. In the open-label extension phase, all baseline and cardinal ulcers in the riociguat-riociguat arm. This observation is a likely indication that a longer duration of riociguat treatment can cause healing of DUs. A similar observation was demonstrated in a RCT of oral treprostinil where the primary outcome of change in net DU burden was not met at week 20 but showed efficacy during the 1-year open-label extension phase when exposed to oral treprostinil [20]. Also, an increase in DU burden was noted in the year after oral treprostinil discontinuation despite adjustment to seasonal variations.

The biomarkers that we chose have been shown to be associated with SSc vasculopathy [21]. At baseline, there were statistically significant elevations in plasma cGMP, sE-selection, and sICAM1 in participants compared to healthy controls, which coincides with the published literature [22–24]. After riociguat treatment, none of the biomarkers measured was significantly altered, except for cGMP, which is expected, and supports the target engagement.

### Strengths and limitations of the study

We included experienced centers with specialized expertise in the management of SSc-related DUs. The wound care of the DUs was standardized across the participating sites. All DUs were defined, photographed, and confirmed by the corresponding author, suggesting a level of standardization in DU assessment. The study is not without limitations. First, we had a small sample size as this was a pilot study to obtain preliminary estimates of treatment effects in efficacy and safety. We also stopped the trial prematurely due to difficulty in recruitment, as majority of current management of DU in the USA includes PDE5 inhibitors. Second, there was a baseline imbalance in the study population with the placebo group having participants with longer disease duration, but the participants in the riociguat group had more severe self-reported disease in terms of RP and DU.

## Conclusions

In conclusion, treatment with riociguat in this trial did not reduce the NUB in patients with SSc. The negative results may reflect lack of power, low NUB at baseline, moderate-to-severe vasculopathy with long-term disease, shorter duration of the trial, and difficulty to recruit patients in the era of widespread use of PDE5 inhibitors. This and other recent trials also highlight the changing epidemiology of SSc-DU due to the availability of somewhat effective pharmacologic therapies such as PDE5 inhibitors, prostacyclin analogs, and better wound care management of these ulcers [20, 25, 26]. Future trials should acknowledge this during the trial design and plan longer trials with background standard of care treatments. There was a trend toward DU healing with longer duration of treatment with riociguat in the open-label extension, but this observation will need to be confirmed in a larger RCT.

## Additional files

Additional file 1: Complete inclusion/exclusion criteria. (PDF 904 kb)

Additional file 2: Further details on the statistical analysis. (PDF 681 kb)

Additional file 3: Comparison of the statsitical change in biomarkers levels from baseline between healthy controls and all patients. (DOCX 23 kb)

## Data Availability

The datasets used and/or analyzed during the current study are available from the corresponding author on reasonable request.

## Abbreviations

SSc: Systemic sclerosis
RP: Raynaud’s phenomenon
DU: Digital ulcers
PDE5: Phosphodiesterase
sGC: Soluble guanylate cyclase
NO: Nitric oxide
DCC: Data Coordinating Center
MCPs: Metacarpophalangeal joints
FRP: Females of reproductive potential
RCS: Raynaud’s Condition Score
HAQ-DI: Health Assessment Questionnaire Disability Index
PROMIS: Patient-Reported Outcomes Measures Information System
HDISS-DU: Hand Disability in Systemic Sclerosis-DU
VAS: Visual analog scale
SHAQ: Scleroderma Health Assessment Questionnaire
VEGF: Vascular endothelial growth factor
tPA: Tissue plasminogen activator
sE-Selectin: Soluble E-selectin
bFGF: Basic fibroblast growth factor
VCAM-1: Vascular cell adhesion molecule-1
sICAM-1: Soluble intracellular adhesion molecule 1
PINP: N-terminal propeptide of type I collagen
MMP12: Matrix metalloproteinase 12
cGMP: Cyclic guanylyl cyclase
sFLT1: Soluble fms-like tyrosine kinase-1
MITT: Modified intention to treat
