# Supplementary material for: A multicenter randomized, double-blind, placebo-controlled pilot study to assess the efficacy and safety of riociguat in systemic sclerosis-associated digital ulcers
Source: Arthritis Res Ther. 2019 Sep 3;21:202. doi: 10.1186/s13075-019-1979-7 (PMC6724329; doi:10.1186/s13075-019-1979-7)
Supplement: Supplementary file 1 — Complete inclusion/exclusion criteria. (PDF 904 kb) [file 13075_2019_1979_MOESM1_ESM.pdf]

Title page

A MULTI-CENTER RANDOMIZED, DOUBLE-BLIND, PLACEBO-CONTROLLED PILOT STUDY TO ASSESS THE EFFICACY AND SAFETY OF RIOCIGUAT IN SCLERODERMA-ASSOCIATED DIGITAL ULCERS

Riociguat in scleroderma-associated digital ulcers

Test drug:	BAY63-2521/ riociguat
Study purpose:	Safety and efficacy of Riociguat
Clinical study phase:	IIa
Version no:	**4**
Version Date	**July 10, 2017**
Sponsor Investigator:	Dinesh Khanna, MD, MSc<br>Professor of Medicine<br>Division of Rheumatology/Dept. of Internal Medicine<br>Suite 7C27<br>300 North Ingalls Street Ann Arbor, MI 48109-5422<br>khannad@med.umich.edu Phone: 734.232-2104<br>Direct: 734.763.7182<br>Fax: 734.763.5761

The study will be conducted in compliance with the protocol, ICH-GCP and any applicable regulatory requirements.

Confidential

The information provided in this document is strictly confidential and is intended solely for the guidance of the clinical investigation. Reproduction or disclosure of this document - whether in part or in full - to parties not associated with the clinical investigation, or its use for any other purpose, without the prior written consent of the sponsor is not permitted.

Throughout this document, symbols indicating proprietary names (®, TM) may not be displayed. Hence, the appearance of product names without these symbols does not imply that these names are not protected.

Signature of Investigator Sponsor

The signatory agrees to the content of the final clinical study protocol as presented.

Signature:

Name: Dinesh Khanna, MD, MSc

Date: July 10, 2017

Synopsis

**Title**	A MULTI-CENTER RANDOMIZED, DOUBLE-BLIND, PLACEBO-CONTROLLED PILOT STUDY TO ASSESS THE EFFICACY AND SAFETY OF RIOCIGUAT IN SCLERODERMA-ASSOCIATED DIGITAL ULCERS
**Short title**	Riociguat in scleroderma-associated digital ulcers
**Clinical study phase**	IIa
**Study objective(s)**	The primary objective of this study is to provide preliminary data on the efficacy (digital ulcer net burden) and safety of riociguat administered 3 times daily (TID) in comparison to placebo in patients with scleroderma-associated digital ulcers
**Test drug(s)**	BAY 63-2521 / riociguat
**Name of active ingredient**	BAY 63-2521 / riociguat
**Dose(s)**	0.5 mg, 1 mg, 1.5 mg, 2 mg and 2.5 mg administered TID; dose titration starting with 1.0 mg (planned up-titration every 2 weeks, with possibility of dose reduction for tolerability; 0.5 mg is the lowest dose and 2.5 mg is the highest dose to be administered)
**Route of administration**	Oral
**Duration of treatment**	Total of 16 weeks, consisting of an 8-week titration period and 8 weeks of maintenance.
**Reference drug(s)**	Placebo
**Name of active ingredient**	Not applicable
**Dose(s)**	Matching placebo tablets to BAY 63-2521/ riociguat 0.5 mg, 1 mg, 1.5 mg, 2 mg and 2.5 mg administered TID; dose titration starting with 1.0 mg matching placebo tablet.
**Route of administration**	Oral
**Duration of treatment**	Total of 16 weeks, consisting of an 8-week titration period and 8 weeks of maintenance.
**Background treatment**	Not applicable
**Indication**	Scleroderma-associated digital ulcers (SSc-DU)
**Site Numbers**	Up to 10 sites in the US. Currently participating sites are listed in this protocol with the opportunity to add up to 5 more if necessary for recruitment

**Diagnosis and main criteria for inclusion**	Men or women aged 18 years and older, inclusive<br>Systemic Sclerosis as defined by 2013 the American College of Rheumatology (ACR) / European League Against Rheumatism (EULAR) classification.<br>Patients had to have at least one visible, active ischemic digital ulcer(DU) or painful indeterminate DU at screening located at or distal to the proximal interphalangeal joint, and that developed or worsened within 8 weeks prior to screening<br>Oral corticosteroids (≤ 10 mg/day of prednisone or equivalent), nonsteroidal anti-inflammatory drugs (NSAIDs), angiotensin converting enzyme (ACE) inhibitors and calcium channel blockers are permitted if the participant is on a stable dose for ≥ 2 weeks before and including at baseline.<br>Women of childbearing potential must have a negative urine pregnancy test at the screening and baseline visit, negative monthly urine pregnancy tests, and must use reliable methods of contraception.
**Study design**	Randomized (1:1), double-blind, placebo-controlled, parallel-group, multicenter, US study
**Methodology**	This study is designed to investigate the efficacy and safety of riociguat (1 mg,<br>1.5 mg, 2 mg and 2.5 mg TID; 0.5 mg is the lowest dose administered if the participant experiences side effects) in patients with SSc-DU. The study consists of two parts: 1) a main double-blind placebo-controlled 16-week study treatment phase, and 2) an optional 16-week open-label extension phase for participants with active digital ulcer/reoccurrence of digital ulcers within 1 month of the end of the main treatment phase.
**Type of control**	Placebo
**Number of participants**	20 randomized participants
**Primary variable**	Change in digital ulcer net burden from baseline to the end of the double-blind study treatment phase

**Plan for statistical analysis**	The planned sample size of 20 SSc participants is based on practical considerations to obtain preliminary estimates of the magnitude of treatment differences in efficacy and safety rather than a desired power for a pre-specified difference as would be necessary for a confirmatory study. However, with this proposed sample of 20 participants (10 riociguat and 10 placebo), we can calculate the magnitude of treatment differences (riociguat – placebo) for the primary efficacy endpoint – the change from baseline to end of double- blind treatment in digital ulcer net burden (a continuous endpoint), or safety outcomes – characterized by the proportion of participants who experience an AE. There would be 80% power to detect an effect size (mean treatment difference divided by standard deviation) of 1.253 or greater with a two-sided type I error of 5% in the primary endpoint. Given the pilot nature of this Phase IIa study, the difference between mean change in digital ulcer net burden between riociguat and placebo that can be detected with sufficient power is large. Similarly, we can calculate power for safety outcomes for this sample size: there is 81% power to detect treatment differences of 51%, assuming 40% of placebo-treated participants experience an AE.<br><br>Descriptive statistics will be derived for all clinical variables, mean and standard deviation for continuous outcomes and percentages for categorical and dichotomous variables. The main outcome of interest is the mean change in digital ulcer net burden at the end of 16 weeks in the two groups. We will compare the mean change in the riociguat vs. placebo groups using an ANCOVA model, with treatment and baseline digital ulcer net burden as covariates. Extension of the Wilcoxon rank-sum test to adjust for baseline digital ulcer net burden will be used if the assumptions of the parametric ANCOVA model are violated. We will also compare the proportion of patients with healing of the cardinal DU, the mean change in number of DUs from baseline to week 16, the number of new DUs during the double blind phase of the study, new ulcers or healing of ulcers over the distal interphalangeal (DIP), proximal interphalangeal (PIP), and metacarpophalangeal (MCP) joints and elbows, and the change in patient-reported outcomes (PROs) including Raynaud's phenomenon (RP) measures and global assessments by the patient and physician in the riociguat vs. placebo group using similar methods as used for the primary endpoint for continuous outcomes and Fisher's exact tests for discrete outcomes.<br><br>The p-values resulting from formal statistical tests will be interpreted from a hypothesis-generating, rather than a confirmatory framework.

Table of contents

1. Introduction......9

1.1 Background......9
1.1.1 Scleroderma (systemic sclerosis)......9
1.1.2 Riociguat......10
1.1.2.1 Mechanism of Action......10
1.1.2.2 Clinical Data......10

1.2 Rationale of the study......11
1.3 Benefit-risk assessment......11

2. Study objectives......12

2.1 Secondary objectives......12

3. Investigators......13

4. Study design......14

4.1 Design overview......14
4.1.1 Screening phase (up to 2 weeks)......15
4.1.2 Double-blind Treatment phase (Week 0 to Week 16)......16
4.1.3 Open-Label Extension phase (OLE)......17
4.1.4 Early Termination Visit and Safety Follow-up Visit......18

5. Study population......18

5.1 Eligibility......18
5.1.1 Inclusion criteria......18
5.1.2 Exclusion Criteria......19
5.1.3 Justification of selection criteria......20

5.2 Withdrawal of participants from study......21
5.2.1 Withdrawal......21

6. Treatment......22

6.1 Treatments to be administered......22
6.2 Identity of study treatment......23
6.3 Treatment assignment......24

6.4 Dosage and administration......24
6.4.1 Selection of doses in the study......24
6.4.2 Special populations......24

6.5 Blinding......25
6.6 Treatment compliance......26
6.7 Post-study therapy......26
6.8 Prior and concomitant therapy......26

7. Procedures and variables......27

7.1 Schedule of events 27
7.1.1 Schedule of Events 29
7.1.2 Timing of assessments 31
7.1.2.1 Visit 0 − Screening........ 31
7.1.2.2 Visit 1 − Baseline (Day 0, Week 0) – Double-Blind Treatment Phase 31
7.1.2.3 Visits 2 through 7 − Dose titration/Stable dosing periods (Double-blind treatment phase) 32
7.1.2.4 Early Termination Visit during Double Blind 33
7.1.2.5 Unscheduled visit 33
7.1.2.6 Visits 8 through 14 − Dose-titration phase/ Stable dosing periods (Open label extension phase) 33
7.1.2.7 Early Termination Visit during Open Label Extension 34
7.1.2.8 Unscheduled visit 34

7.2 Population characteristics 34
7.2.1 Demographic 34
7.2.2. Medical history 35
7.3 Efficacy 35
7.4 Pharmacokinetics / pharmacodynamics 36

7.5 Safety 36
7.5.1 Adverse events 36
7.5.1.1 Definitions 36
7.5.1.2 Classifications for adverse event assessment 38
7.5.1.2.1 Seriousness 38
7.5.1.2.2 Intensity 38
7.5.1.2.3 Causal relationship 38
7.5.1.2.4 Action taken with study treatment 39
7.5.1.2.5 Other specific treatment(s) of adverse events 39
7.5.1.2.6 Outcome 40
7.5.1.3 Assessments and documentation of adverse events 40
7.5.1.4 Reporting of serious adverse events 40
7.5.1.5 Expected adverse events 41
7.5.1.6 Adverse events of special safety interest 41
7.5.1.7 Overdose of Study Medication 41
7.5.2 Pregnancies 41

7.6 Other procedures and variables 41
7.6.1 Pregnancy testing 41
7.6.2 Laboratory parameters 42
7.6.3 Exploratory Biomarkers 42
7.6.4 Blood pressure and heart rate measurement 42
7.6.5 Digital ulcer net burden 42
7.6.6 Digital Gangrene 43
7.6.7 Raynaud's attacks assessment 43
7.6.8 Patient-Reported Outcomes (PROs) / Health-Related Quality of Life (HRQoL) questionnaires 43
7.6.9 Participant and Physician Global Assessment 44

8. Statistical methods and determination of sample size 44
8.1 General considerations 44
8.2 Analysis sets 45
8.3 Statistical and analytical plans 45
8.3.1 Demographic and other baseline characteristics 45
8.3.2 Efficacy 45
8.3.3 Safety 46
8.4 Planned interim analyses 46
8.5 Determination of sample size 46
8.6 Data recording 47
8.7 Monitoring 47
8.8 Archiving 47
9. Premature termination of the study 48
10. Ethical and legal aspects 49
10.1 Ethical and legal conduct of the study 49
10.2 Compensation for health damage of participants / insurance 49
11. Appendices 49
11.1 Child-Pugh Classification of Liver Disease 49
11.2 Digital Ulcer Care Protocol 50
12. References 51

1. Introduction

1.1 Background

1.1.1 Scleroderma (systemic sclerosis)

Systemic sclerosis (SSc) is a rare, orphan disease featuring chronic, fibrosing, autoimmune responses characterized by small vessel vasculopathy, autoantibody production, and fibroblast dysfunction leading to increased deposition of extracellular matrix(1).

Systemic sclerosis is further divided into 2 subtypes defined by the extent of skin involvement: limited cutaneous systemic sclerosis (lcSSc) and diffuse cutaneous systemic sclerosis (dcSSc). In limited disease (formerly called CREST [calcinosis, Raynaud's phenomenon, esophageal dysmotility, sclerodactyly, and telangiectasias] syndrome), skin tightening is confined to the fingers, hands, and forearms distal to the elbows, with or without tightening of skin of the feet and of the legs distal to the knees. Proximal extremities and the trunk are not involved. In dcSSc, the skin of the proximal extremities and trunk is also involved. Both dcSSc and lcSSc are associated with internal organ involvement; however, patients with dcSSc are at greater risk for clinically significant major organ dysfunction. dcSSc is one of the most fatal rheumatic diseases, and is associated with substantial morbidity and many detrimental effects on health-related quality of life.

SSc is an orphan disease with an estimated prevalence of 50,000 in Europe and 276 per 100,000 in the United States (US). Applying these estimates to the 2010 US census of 309 million people, the project number of patients in the US with SSc is less than 100,000. Approximately 60% have lcSSc and 35-40% have dcSSc.

Raynaud’s phenomenon (RP) is an almost universal manifestation of SSc, with 95% of all patients being affected, and resulting in digital ulcers (DUs) in approximately 30% of the patients each year. DUs are a major clinical problem, being associated with substantial morbidity (reduced quality of life, pain, disability and disfigurement) that can escalate to gangrene and amputation in approximately 15% of patients. Treatments that have shown potential include calcium channel blockers, prostacyclin analogues and endothelin receptor antagonists (ERAs) . Bosentan, a dual endothelin receptor antagonist, is approved in Europe to reduce the number of new DUs in patients with SSc. However, bosentan is not approved in United States and does not appear to heal the ulcer. Small studies and case series have shown efficacy of PDE-5 inhibitors in scleroderma-spectrum DU and this finding is supported by a meta-analysis(2).

Riociguat is the first-in-class of a new group of compounds, soluble guanylate cyclase (sGC) stimulators. Riociguat directly stimulates sGC, thereby increasing levels of the signaling molecule cGMP. The cGMP molecule plays a pivotal role in regulating cellular processes, such as vascular tone, proliferation, fibrosis, and inflammation. Two key features of riociguat are (i) it directly stimulates sGC independently of nitric oxide (NO), and (ii) it sensitizes sGC to low levels of NO.

Riociguat has recently been approved in the US and Canada for two forms of pulmonary hypertension, namely pulmonary arterial hypertension (PAH) and treatment of chronic thromboembolic pulmonary hypertension (CTEPH).

From the literature, there is ample evidence from *in vitro* and *in vivo* investigations for efficacy of cGMP elevation in fibrotic diseases and a large trial is planned in skin fibrosis associated with SSc(3-6).

The goal of the current study is to provide preliminary data on the efficacy and safety of 16 weeks of treatment with riociguat in a randomized, placebo-controlled clinical trial in patients with SSc-associated DU.

1.1.2 Riociguat

1.1.2.1 Mechanism of Action

As described above, riociguat is the first-in-class sGC stimulator that directly stimulates sGC independently of NO and sensitizes sGC to low levels of NO. Because of the broad spectrum of cGMP actions and specific mode of action of riociguat, the efficacy of sGC stimulators for the treatment of patients with systemic sclerosis (SSc) was investigated. Both, the vascular effects of riociguat and the direct antifibrotic effects could be beneficial for the treatment of patients with SSc. More specifically, it was shown that sGC stimulators decrease collagen production in dermal fibroblasts from SSc patients. In addition, sGC stimulators block fibroblast to myofibroblasts differentiation of dermal fibroblasts. Thus, sGC stimulators could block production of collagen and extracellular matrix components, which may explain, at least in part, the antifibrotic effects of sGC stimulators.

1.1.2.2 Clinical Data

Riociguat (Adempas) was recently approved in the US and Canada for the treatment of PAH and/or CTEPH.

The safety data described below reflect exposure to riociguat in two, randomized, double-blind, placebo-controlled trials in patients with inoperable or recurrent/persistent CTEPH (CHEST-1) and treatment naive or pre-treated PAH patients (PATENT-1). The population (Riociguat: n = 490; Placebo: n = 214) was between the age of 18 and 80 years.

The safety profile of riociguat in patients with inoperable or recurrent/persistent CTEPH (CHEST-1) and treatment naive or pre-treated PAH (PATENT-1) were similar. Therefore, adverse drug reactions (ADRs) identified from the 12- and 16-week placebo-controlled trials for PAH and CTEPH, respectively, were pooled, and those occurring more frequently on riociguat than placebo (≥3%) are displayed in Table 1 below.

Most adverse reactions in Table 1 can be ascribed to the vasodilatory mechanism of action of riociguat. The overall rates of discontinuation due to an adverse event in the pivotal placebo-controlled trials were 2.9% for riociguat and 5.1% for placebo (pooled data).

**Adverse Reactions**	**Adempas % (n=490)**	**Placebo % (n=214)**
Headache	27	18
Dyspepsia and Gastritis	21	8
Dizziness	20	13
Nausea	14	11
Diarrhea	12	8
Hypotension	10	4
Vomiting	10	7
Anemia (including laboratory parameters)	7	2
Gastroesophageal reflux disease	5	2
Constipation	5	1

Further details can be found in the Package Insert (PI), which contains comprehensive information on the study drug.

1.2 Rationale of the study

DUs occur in approximately 30% of the patients each year (2). DUs are a major clinical problem, being associated with substantial morbidity (reduced quality of life, pain, disability and disfigurement) that can escalate to gangrene and amputation in approximately 15% of patients. There are no drugs approved in the US for treatment of DU. Riociguat is a direct stimulator of the sGC *in vitro* and *in vivo* which is independent from NO, the endogenous activator of the enzyme. Moreover, in the presence of NO, it enhances the effects of NO.

The sGC catalyzes the production of cyclic guanosine monophosphate (cGMP) upon binding of NO. Increased intracellular cGMP levels induce vasorelaxation, inhibition of cell proliferation and migration as well as inhibition of platelet adhesion and aggregation.

Because various preclinical *in vitro* and *in vivo* data revealed that riociguat was safe and efficacious in PAH, the hypothesis is that riociguat may bring significant clinical benefit to patients with scleroderma by improving vasculopathy. In addition, riociguat has anti-fibrotic action that may augment the benefits in the vascular pathway.

1.3 Benefit-risk assessment

There are currently no disease-specific pharmacotherapies approved for healing of DU and therefore there is a high unmet medical need in this indication. During the pivotal riociguat PATENT-1 trial for PAH, participants with PAH-SSc were included and the overall benefit-risk profile was positive.

Considering the mechanism of action of riociguat and the results of the PAH trials, we believe that riociguat will:

- Decrease digital ulcer net burden.
- Heal digital ulcers.
- Decrease number and severity of Raynaud's attacks.

Taking into account the seriousness of the disease as well as the medical need for an effective and safe therapy, on balance the expected benefit to participants with this life-threatening condition outweighs potential risks.

2. Study objectives

The primary objective of this study is to provide preliminary data on the efficacy (digital ulcer net burden) of riociguat administered 3 times daily (TID) in comparison to placebo in patients with SSc.

The primary efficacy outcome is the change from baseline to end of double-blind treatment in digital ulcer net burden. Digital ulcer net burden is defined as the total number of "active" and indeterminate digital ulcers at an assessment.

2.1 Secondary objectives

The secondary objectives of this study are to provide preliminary data on safety and additional measures of efficacy of riociguat administered TID as compared with placebo.

Additional efficacy measures include:

- Healing of the cardinal DU. For each participant, one digital ulcer must be identified and designated by the investigator as the cardinal ulcer at screening. The cardinal ulcer must have met the qualifications for designation as an active or painful indeterminate ulcer. If only one ulcer was determined at entry to be active or painful indeterminate, it will be designated as the cardinal ulcer. If several digital ulcers qualified, the cardinal ulcer could be either the largest or the most painful ulcer, or the ulcer that disturbed the patient the most. The cardinal ulcer will be selected by the investigator based on the clinical judgment that it was amenable to and evaluable for healing.
- Development of DU
- Development and healing of pressure ulcers over the Distal Interphalangeal (DIP), Proximal Interphalangeal (PIP), Metacarpophalangeal (MCPs) and elbows.
- Time to healing of cardinal DU
- Time to healing of all DU
- Improvement of Raynaud's phenomenon (RP) (7, 8)
    - Raynaud's condition score
    - Number of Raynaud's attacks/day
    - Patient and physician assessment of RP; pain, numbness, and tingling during an

RP attack; and duration of attacks

- Patient's and physician's global assessment on a Likert scale
- Health-related quality of life (HRQOL) using PROMIS-29
- Physical function as assessed by HAQ-DI and HDISS-DU
- Visual analog scales from scleroderma-health assessment questionnaire (SHAQ) assessing burden of digital ulcers, Raynaud's disease, gastrointestinal involvement, breathing, and overall disease
- Digital ischemia requiring intravenous prostacyclin or digital gangrene or amputation
- Vascular biomarkers in the plasma (VEGF, tPA, sE-Selectin, BFGF, VCAM-1, ICAM)

Safety outcomes include adverse events, clinically significant changes in vital signs, laboratory test abnormalities, and clinical tolerability of the drug.

3. Investigators

Title/Name:	Site Investigator, Dinesh Khanna, MD, MSc, University of Michigan
MD, Address:	300 North Ingalls Street, NI7C27<br>Ann Arbor, MI 48109-5422<br>USA
Telephone No:	+1(734) 763-7182
Fax No:	+1(734) 763-5761

Title/Name:	Site Investigator, Virginia Steen
MD, Address:	Georgetown University Hospital<br>Department of Rheumatology, Immunology, & Allergy<br>3800 Reservoir Road, NW<br>3PHC, Suite 3004E<br>Washington, DC 20007
Telephone No:	+1(202) 444-6200

Title/Name:	Site Investigator, Robyn T. Domsic, MD, MPH
MD, Address:	University of Pittsburgh School of Medicine<br>S726 Biomedical Science Tower<br>3500 Terrace Street<br>Pittsburgh, PA 15261
Telephone No:	+1 (412) 648-7040

Title/Name:	Site Investigator, Tracy Frech, MD
MD, Address:	University of Utah, Division of Rheumatology<br>30 North 1900 East 4B200 SOM<br>Salt Lake City, UT 84132
Telephone No:	+1(801) 581-4333

Title/Name:	Site Investigator, Jessica Gordon, MD
MD, Address:	Hospital of Special Surgery, Division of Rheumatology<br>535 East 70th Street

New York, NY 10035
Telephone No: +1(212) 774-7381

Data Safety and Monitoring Board

An independent data and safety monitoring board (DSMB) will be convened that will review safety data. The purpose of the DSMB is to assure independent review as to whether study participants are exposed to unreasonable risk because of study participation. Detailed information on the roles and responsibilities of the DSMB will be described in the DSMB Charter.

4. Study design

4.1 Design overview

This clinical trial is a US, multicenter, double-blind, randomized placebo-controlled, parallel-group study with a total of 20 participants planned to be randomized (approximately 10 participants to the riociguat group and 10 to the placebo group). In addition, a standardized wound care protocol (see Appendix 11.2) will be followed by the investigators and digital photography will be taken of the cardinal ulcer.

The study will allow standard of care medications for the management of DU as background therapy. These may include calcium channel blockers, low dose aspirin, angiotensin enzyme inhibitors, etc. and will be determined by the participant’s local physician.

The study design consists of three phases:

- **Screening phase:** up to 2 weeks

- **Double-blind Treatment phase:** 16 weeks of double-blind treatment, consisting of:
  - Dose titration period of up to 8 weeks, and
  - Stable dosing period of up to 8 weeks

- **Open-label Extension phase** for participants with active or painful indeterminate DU at the end of the double- blind treatment phase or development of an active or painful indeterminate DU within a month of completing double-blind phase, consisting of:
  - Dose titration phase of up to 8 weeks
  - Stable dosing period for 8 weeks

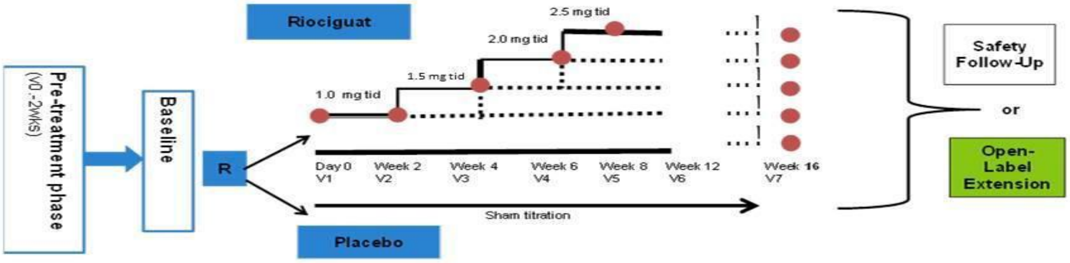

Figure 4-1. Main Study Treatment phase design
Abbreviations: V = visit; R = randomization; TID = 3 times a day.

4.1.1 Screening phase (up to 2 weeks)

After providing written informed consent, participants will undergo a screening evaluation to determine their eligibility (see Section 7.1 for a detailed schedule of events). Participants will complete a diary detailing the number and duration of Raynaud's attacks per day for a period of at least 7 consecutive days leading up to the start of treatment / Day 0.
If the Raynaud's attack diary can be completed prior to the screening visit it will allow the patient with a painful ulcer to be screened and randomized the same day thereby eliminating the burden of an additional clinic visit.

4.1.2 Double-blind Treatment phase (Week 0 to Week 16)

At the baseline visit, participants who have met all of the inclusion and none of the exclusion criteria will be randomized in 1:1 fashion to riociguat or placebo using a web-based randomization system.

Dose titration phase (Visits 1 to 5)

In the first 8 weeks of the double-blind treatment phase, participants will undergo individual dose titration.

The starting dosage is 1.0 mg TID. The dose should be increased by 0.5 mg increments no sooner than 2 weeks(± 4 days) apart to 1.5 mg, 2 mg and 2.5 mg TID, resulting in a maximum total daily dose of 7.5 mg (2.5 mg TID). Participants can be maintained on a lower dose if higher doses are not tolerated (minimum dosage of 0.5 mg TID, total daily dose 1.5 mg). It is possible for a participant to be up-titrated and then down-titrated during the course of this phase of the study.

However, once a participant has been down-titrated, they will remain at that dose and escalation will not be implemented again. If necessary, a participant can down-titrate multiple times to a minimum of 0.5 mg TID.

To maintain blinding of the treatment arms, participants randomized to the placebo group will undergo sham titration from Visit 1 onwards during the dose-titration period following the rules of the individual dose titration scheme.

Individual dose titration algorithm

The individual study medication dose for the next titration step will be determined every 2 weeks according to the patient's well-being and the peripheral systolic blood pressure (SBP) measured at trough before intake of the morning dose according to the following algorithm (individual dose titration scheme):

- If SBP is ≥ 95 mmHg and the participant has no signs or symptoms of hypotension, the dosage should be increased by 0.5 mg TID. If the participant has signs or symptoms of hypotension, the dose should be decreased by 0.5 mg TID, regardless of SBP.
- If SBP is < 95 mmHg, the dosage should be maintained provided the participant does not show any signs or symptoms of hypotension.
- If SBP is < 95 mmHg and the participant exhibits signs or symptoms of hypotension the current dosage should be decreased by 0.5 mg TID.
- If the participant develops SBP < 95 mmHg or exhibits signs or symptoms of hypotension on 0.5 mg TID, then the participant is discontinued from study medication but should continue with follow-up visits. The participant may continue in the open label phase at the discretion of the PI if an active or painful indeterminate DU is present at week 16 or week 20.

Stable dosing period (Visits 6, and 7)

The overall duration of the double-blind treatment phase is 16 weeks, including the dose titration period. At the end of the dose titration period (Week 8/Visit 5), the participant's stable dose for the next 8 weeks will be determined using the treatment algorithm above. No further increase in dose will be allowed. The established individual dose will then be taken as the "optimal individual dose" to be administered for the remaining duration of the main study (up to Week 16).

Dose reductions for safety reasons (e.g., in case of any treatment-emergent adverse event [AE]) are allowed, but a subsequent dose increase during the maintenance period is not permitted.

Dose Interruptions

If treatment is interrupted between visits for any reason other than signs and symptoms of hypotension, the following rules should be applied:

- ≤ 3 consecutive days without treatment: restart with last dose
- > 3 days but ≤ 14 consecutive days without treatment it is at the discretion of the investigator whether the study medication can be restarted: if restarted, start with last dose
- > 14 consecutive days without treatment: withdraw the participant from study medication (but continue follow-up in the study).

The algorithm below will be followed for dose titration at study visits 2-5 and OL Visits 9-12 if treatment is interrupted for any length of time:

# of missed days (consecutive)	No Signs or symptoms of hypotension and SBP ≥ 95 mmHg	Signs or symptoms of hypotension present (any SBP)	No signs or symptoms of hypotension but SBP < 95 mmHg
0	Up titrate	Down Titrate	Same Dose
≤ 3 and missed days do not include day prior to visit	Up titrate	Down Titrate	Same Dose
≤ 3 and missed days include day prior to visit	Same Dose	Down Titrate	Same Dose
4-14	Same Dose	Down Titrate	Same Dose
> 14	Stop Study Med	Stop Study med	Stop Study med

4.1.3 Open-Label Extension phase (OLE)

At Visit 7 (Week 16), all participants with active or painful indeterminate DU will be offered the opportunity to initiate active treatment with riociguat, as part of the open-label phase.

At Visit 7 (Week 16), participants without an active or painful indeterminate DU will stop double-blind therapy and be scheduled for a Safety Follow-up at Week 20. Those who have a reoccurrence of DU prior to and including their scheduled Week 20 visit (i.e., the 4 week period after the end of the double- blind period), will be offered open-label riociguat. Those who do not develop an active DU by Week 20 will terminate the study at the Week 20 visit.

- All participants who agree to remain in the study for the OLE, regardless of their original randomized treatment, will receive riociguat for an additional 16 weeks according to the dose-titration and stable dosing regimen that was used in the double blind phase, starting with 1.0 mg riociguat.

Figure 7.1 provides a flow diagram of the end of double-blind to either end-of-study or start of open-label extension phases of the study.

<u>Dose Titration Phase (Visits 7 to 12)</u>

At Visit 7 (Week 16), all participants who agree to continue in the open-label extension will be assigned to treatment with riociguat. During the first 8 weeks of the open-label extension phase, participants previously on placebo will be up-titrated on riociguat according to the individual titration algorithm described above in the double-blind treatment phase. Participants randomized to riociguat in the double-blind study treatment phase will also undergo titration (to protect the blind of the double-blind portion of the study).

Stable dosing period (Visits 13 and 14)

Stable dosing will continue for another 8 weeks.

4.1.4 Early Termination Visit and Safety Follow-up Visit

A termination visit and a safety follow-up visit (4 weeks after last dose of study medication) should be performed for all patients who withdraw from study at any time during the double blind phase. A termination visit should be performed for anyone who withdrawals early during the OLE. If there are no study medication related Adverse events nor any digitial ulcers the follow-up visit can be performed as a phone call to alleviate the participant's burden of an in person clinic visit.

5. Study population

5.1 Eligibility

5.1.1 Inclusion criteria

Participants must meet the following criteria to be eligible for enrollment in the study:

1. Signed written informed consent
2. Men or women aged 18 years and older
3. Diagnosis of Systemic sclerosis, as defined by 2013 American College of Rheumatology/ European Union League Against Rheumatism classification of SSc
4. Patients had to have at least one visible, active ischemic DU or painful indeterminate DU at screening located at or distal to the proximal interphalangeal joint, and that developed or worsened within 8 weeks prior to screening.

Figure 5.1

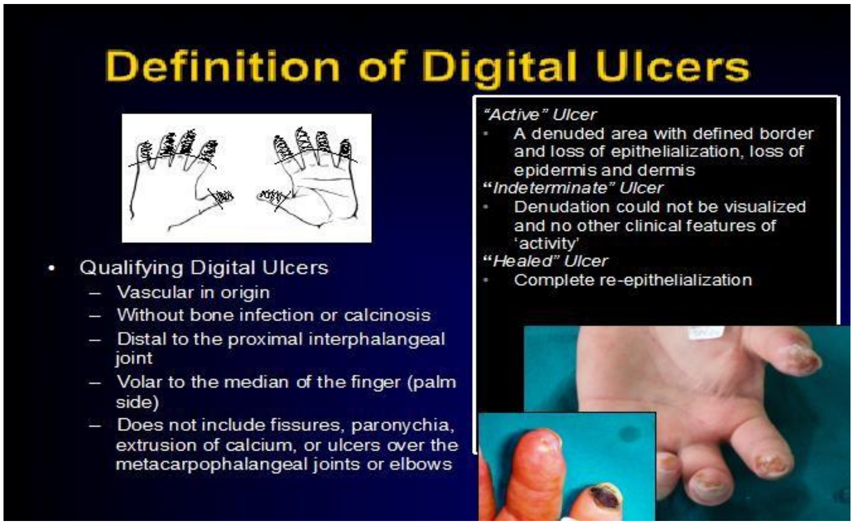

5. Females of reproductive potential (FRP) must have a negative, pre-treatment urine pregnancy test.
6. FRP must obtain monthly urine pregnancy tests during treatment and one month after treatment discontinuation. Post-menopausal women (defined as no menses for at least 1 year or post-surgical from bilateral oophorectomy) are not required to undergo a pregnancy test.
7. FRP and all non-vasectomized male participants must agree to use reliable contraception when sexually active. (For FRP's, 'Adequate contraception' is defined as any combination of at least 2 effective methods of birth control, of which at least one is a physical barrier (e.g., condoms with hormonal contraception or implants or combined oral contraceptives, certain intrauterine devices). This applies from the time of signing the informed consent form until one month after the last study drug administration.)
8. Oral corticosteroids (≤ 10 mg/day of prednisone or equivalent), nonsteroidal anti-inflammatory drugs (NSAIDs), angiotentin receptor blockers, angiotensin converting enzyme (ACE) inhibitors and calcium channel blockers are permitted if the participant is on a stable dose for ≥ 2 weeks prior to and including the baseline visit
9. Ability to comply with the clinical visits schedule and the study-related procedures.

5.1.2 Exclusion Criteria

Participants who meet any of the following criteria will be excluded from enrollment in the study:

1. Active DU related to calcinosis (as assessed by clinical examination or radiographic evaluation at screening)
2. Medical and surgical history
    - Major surgery (including joint surgery) within 8 weeks prior to screening
    - Participants with a history of malignancy in the last 5 years other than non-melanoma skin cell cancers cured by local resection or carcinoma in situ
3. Hepatic-related criteria
    - Hepatic insufficiency classified as Child-Pugh C at screening (see Appendix 11.1

for classification table) at screening visit

4. Renal-related criteria
    - Estimated glomerular filtration rate (eGFR) < 15 mL/min/1.73m^2 (MDRD formula) or on dialysis at the screening visit
5. Cardiovascular-related criteria
    - Sitting systolic blood pressure < 95 mmHg at the screening visit
    - Sitting heart rate < 50 beats per minute (BPM) at the screening visit
    - Left ventricular ejection fraction < 40% prior to screening on echocardiogram done as part of clinical care
6. Pulmonary-related criteria
    - Active state of hemoptysis or pulmonary hemorrhage, including those events managed by bronchial artery embolization
    - Any history of bronchial artery embolization or massive hemoptysis within 3 months prior to screening. Massive hemoptysis being defined as acute bleeding >240 mL in a 24-hour period or recurrent bleeding >100 mL/d over several days
    - PAH requiring pharmacologic therapy.
    - Significant pulmonary disease with FVC ≤ 50% of predicted, or DLCO (uncorrected for hemoglobin) ≤ 40% of predicted
7. Laboratory examinations
    - Participants with hemoglobin < 9.0 g/dL, white blood cell (WBC) count < 3000/mm^3 (< 3 × 10^9/L), platelet count < 100,000/mm^3 (< 3 × 10^9/L) at the screening visit
8. Prior and concomitant therapy
    - Concomitant use of nitrates or NO donors (such as amyl nitrate) in any form, including topical; phosphodiesterase (PDE) 5 (PDE5) inhibitors (such as sildenafil, tadalafil, vardenafil); and nonspecific PDE5 inhibitors (theophylline, dipyridamole). **If the patient is on PDE5 inhibitors, a wash out of 3 days is required for sildenafil and 7 days for tadalafil or vardenafil prior to the baseline visit**
    - Concomitant Endothelin receptor antagonist
    - Patients who are actively smoking at time of consent. (Quit date of two weeks prior to screening acceptable)
9. Pregnant or breastfeeding women
10. Other
    - Any other condition or therapy that would make the participant unsuitable for this study and will not allow participation for the full planned study period
    - Participation in another clinical study with an investigational drug or medical device within 30 days prior to randomization (phase I-III clinical studies)

Note: One re-assessment of laboratory parameters is allowed during the screening phase to assess the eligibility of participants.

5.1.3 Justification of selection criteria

The selection criteria were carefully selected to exclude participants from the study who may

potentially be exposed to specific risks after administering the study drug as well as participants with conditions that may have an impact on the aims of this study.

5.2 Withdrawal of participants from study

5.2.1 Withdrawal

Participants *must* be withdrawn from the study for the following reasons:

- At their own request or at the request of their legally acceptable representative
  At any time during the study and without giving reasons, a participant may decline to participate further. The participant will not suffer any disadvantage as a result.
- If, in the investigator's opinion, continuation of the study would be harmful to the participant's well-being
- Occurrence of AEs or intercurrent diseases which the investigator judges unacceptable for continuation of participation in the study
- Occurrence of adverse drug reactions, which in the investigator’s opinion have a negative impact on the participant’s individual risk-benefit ratio. (Investigators are obliged to reassess the participant’s individual risk-benefit ratio on a continuous basis. Factors such as anticipated treatment effect, progression of underlying disease, occurrence of side effects, and alternative treatment options must be considered.)
- Non-compliance with the conditions for the trial or instructions by the investigator that could negatively affect the participant or impact the validity of the clinical trial.
- Although not preferred, participants may interrupt their intake of study medication for reasonable circumstances/reasons at any time (e.g., hospitalization in a remote hospital without study medication access, safety reasons, and side effects). If an interruption lasts longer than 14 consecutive days, the participant must be withdrawn from treatment, but continue with follow-up visits. The participant will have the opportunity to participate in OLE if s/he meets the criteria. In case treatment requires interruption for > 3 days and ≤ 14 days, it is at the discretion of the investigator if the study medication can be restarted.
- In case of pregnancy or breast feeding.
- In case a female patient of childbearing potential is not compliant with 4-weekly pregnancy testing.
- Participation in another clinical trial. Participation in observational registries is allowed.
- In case no further dose reduction is possible and the participant does not tolerate the lowest possible riociguat dose (0.5 mg TID).

Participants *may* be withdrawn from the study for the following reasons:

- At the specific request of the sponsor and in liaison with the investigator (e.g., safety concerns).
- Development of DU complications requiring prostacyclin therapy, gangrene, or amputation.

Escape therapy

There is no escape therapy. If a participant decides to stop study medication due to AEs or lack of efficacy, they will be encouraged to stay in the study and will be treated by the physician using standard of care.

All reasons for withdrawal (including lost to follow up), will be recorded in the eCRF and site medical record.

In the event that a participant is lost to follow-up, every possible effort must be made by study site personnel to contact the participant to obtain complete data and determine the reason for withdrawal.

A participant may withdraw from further participation in the study and still allow further release of information. In this situation, the participant's consent to the collection of further data should be documented in the site's source documents.

6. Treatment

6.1 Treatments to be administered

Test Drug:	Riociguat/ placebo
Dosage:	0.5 mg, 1 mg, 1.5 mg, 2 mg and 2.5 mg (individual dose titration)
Route of administration:	Oral
Time and frequency of administration:	TID

The individual optimal dose should be determined during the initial 8-week dose titration period based on monitoring of the participant's systolic blood pressure and well-being.

The starting dose is 1.0 mg TID. The intervals between drug intake should be 6-8 hours. The dosage should be increased by 0.5 mg increments no sooner than 2 weeks apart to 1.5 mg, 2 mg, and 2.5 mg TID, resulting in a maximum total daily dose of 7.5 mg (2.5 mg TID). Participants should be maintained on lower doses if higher doses are not tolerated (minimum dose of 0.5 mg TID.). If the participant cannot tolerate 0.5 mg po TID, then the participant is discontinued (see section 5.2.1). After the dose titration period, riociguat should be continued at the optimal individual dose for the duration of the stable dosing period.

6.2 Identity of study treatment

Riociguat

Riociguat is supplied in various strengths as film-coated, immediate-release tablets. (see Table 0-1)

All study drugs will be labeled according to the requirements of local law and legislation. Label text will be approved according to the sponsor's agreed procedures, and a copy of the labels will be made available to the study site upon request.

For all study drugs, a system of numbering in accordance with all requirements of Good Manufacturing Practice (GMP) will be used, ensuring that each dose of study drug can be traced back to the respective bulk ware of the ingredients. Lists linking all numbering levels will be maintained by the sponsor's clinical supplies Quality Assurance group.

A complete record of batch numbers and expiry dates of all study treatment, as well as the labels, will be maintained in the sponsor study file. Almac will act as central distributor for the drug and coordinate drug distribution with the clinical sites.

Table 0-1 Identity of investigational product

International non-proprietary name (INN)	Riociguat
Sponsor's internal reference number	BAY 63-2521
Formulation	Film-coated tablets
Galenical form	Round immediate-release tablets, diameter 6 mm

Composition	Active ingredient:	*Methyl 4,6-diamino-2-[1-(2- fluorobenzyl)-1H-pyrazolo [3,4 b]pyridine-3-yl]-5-pyrimidinyl (methyl)carbamate*
	Empirical formula:	C20H19FN8O2
	Molar mass:	422.42 g/mol (molarity)
	Excipients:	lactose, microcrystalline cellulose, magnesium stearate, crospovidone* , hypromellose*, and sodium lauryl sulphate*
	Coating:	hydroxypropyl cellulose, hypromellose, propylene glycol, iron oxide (red and yellow), and titanium dioxide.
Strength	0.5 mg, 1 mg, 1.5 mg, 2 mg, 2.5 mg	
Material numbers	83296470 BAY 63-2521 TABL 0.5 mg 511 COAT<br>83296535 BAY 63-2521 TABL 1 mg 512 COAT<br>83296543 BAY 63-2521 TABL 1.5 mg 513 COAT<br>83296578 BAY 63-2521 TABL 2 mg 504 COAT<br>83296608 BAY 63-2521 TABL 2.5 mg 515 COAT	
Packaging	High-density polyethylene (HDPE) bottles	

Abbreviation: TABL = tablet
*Not in placebo tablets

Matching placebo

Matching placebo tablets will appear identical to active riociguat tablets but will not contain active study drug product (riociguat) or any of the asterisked excipients as outlined in Table 0-1.

Riociguat and placebo tablets will have similar appearance at all dose strengths. They will be labeled "BAY 63-2521/placebo" and a med number that will be assigned by IVRS.

All study drugs will be labelled according to the requirements of local law and legislation.

Label text will be approved according to the sponsor's agreed procedures, and a copy of the labels will be made available to the study site upon request.

For all study drugs, a system of numbering in accordance with all requirements of Good Manufacturing Practice (GMP) will be used, ensuring that each dose of study drug can be traced back to the respective bulk ware of the ingredients. Lists linking all numbering levels will be maintained by the sponsor's clinical supplies Quality Assurance group.

A complete record of batch numbers and expiry dates of all study treatment, as well as the labels, will be maintained in the sponsor study file.

Storage requirements:

All investigational drugs used during the trial will be stored at the investigational sites at room temperature in a place inaccessible to unauthorized personnel, i.e. in a locked cabinet.
No special storage conditions are required.

6.3 Treatment assignment

This is a randomized, double-blind, placebo-controlled, parallel-group, multicenter study. Participants who complete all screening procedures and meet all the eligibility criteria are to be randomized in a 1:1 ratio to receive either riociguat or placebo. The randomization schedule will be prepared by the Data Coordinating Center (DCC) using a computer-generated block randomization with the block size(s) known only by the DCC statistician

6.4 Dosage and administration

6.4.1 Selection of doses in the study

Riociguat has been safe and well tolerated in previous clinical studies at multiple doses between 0.5 and 2.5 mg TID in patients with various forms of PH, including PAH, CTEPH, and, in a small patient population, SSc-related PH. The starting dose for this study will be at 1.0 mg PO TID.

6.4.2 Special populations

Riociguat has not been studied in all populations. Particular care should be exercised when dosing the following:

Elderly

In elderly patients (≥65 years) particular care should be exercised during individual dose titration as this population showed a higher exposure.

Renal impairment

Patients with mild, moderate or severe renal impairment (creatinine clearance 15-80 mL/min) showed a higher exposure to riociguat. Patients with creatinine clearance <15 mL/min or on dialysis have not been studied. Therefore, the use of riociguat is not recommended in these patients who are consequently excluded from the study.

Hepatic impairment

Patients with moderate hepatic impairment (Child- Pugh B) showed a higher exposure to riociguat. Patients with severe hepatic impairment (Child-Pugh C) have not been studied (Appendix 11.1). Therefore, the use of riociguat is not recommended in these patients who are consequently excluded from the study.

Smokers

Plasma concentrations in smokers are reduced by 50-60% compared to nonsmokers. Safety and effectiveness of riociguat doses higher than 2.5 mg three times a day have not been established. Smokers should not be enrolled in this study. Participants who have quit at least two weeks prior to screening may be considered as a "non-smoker.

6.5 Blinding

The study will be conducted in double-blind fashion. Active riociguat and placebo tablet formulations will be identical in appearance (size, shape, color) and smell. The packaging and labeling will be designed to maintain blinded conditions for the investigator's team and the participants. The study data will remain blinded until database lock and authorization of data release according to standard operating procedures.

Unblinding by drug safety personnel of the sponsor

For regulatory reporting purposes, drug safety personnel of the sponsor are permitted to unblind individual cases.

In compliance with applicable regulations, in the event of a suspected unexpected serious adverse reaction (SUSAR) (see Section 7.5.1.4), the participant's treatment code will usually be unblinded before reporting to the health authorities, Independent Ethics Committees / Institutional Review Boards (IRBs), and investigators (see Section 7.5.1.4) if the SUSAR was

related to the blinded treatment.

Emergency unblinding by the investigator

In case of emergency, investigators are permitted to unblind individual cases. However, investigators are obligated to restrict such unblinding to cases of emergency where the unblinding result is of importance for the acute treatment strategy.

Investigators should note that the occurrence of an SAE should not routinely precipitate the immediate unblinding of the label. If unblinding is necessary for the treatment of a participant with an SAE, every attempt should be made to contact the investigator sponsor prior to unblinding.

If a participant misses a menstrual period or suspects that they are pregnant, they should stop study medication immediately and should be unblinded to determine if they have received riociguat if the pregnancy is confirmed .

Drug logistics and accountability

All study drugs will be stored at the investigational site in accordance with Good Clinical Practice (GCP) and GMP requirements and the instructions given by the clinical supplies department of the sponsor, and will be inaccessible to unauthorized personnel. Special storage conditions and a complete record of batch numbers and expiry dates can be found in the sponsor study file; the site-relevant elements of this information will be available in the investigator site file. The responsible site personnel will confirm receipt of study drug and will use the study drug only within the framework of this clinical study and in accordance with this protocol. Receipt, distribution, return and destruction (if any) of the study drug must be properly documented according to the sponsor's agreed and specified procedures.

Written instructions on medication destruction will be made available to affected parties as applicable.

6.6 Treatment compliance

A drug dispensing log will be completed for each participant. The date of dispensing the study drug to the participant will be documented.

Participants will receive study medication dispensed per visit schedule (see Section 7.1).

To record treatment compliance, participants will be instructed to bring all study drug packaging, including unused study drug and empty packaging, to the investigative site at each study visit. Tablets will be counted for a compliance check.

If a dose of study drug is missed, the participant should take a dose immediately and continue with the TID intake as recommended on the following day. The dose should not be doubled to make up for a missed dose within the same day.

Participants should not take study drug the day of their clinic visit at the end of the double-blind period of the study (Visit 7/Week16).

6.7 Post-study therapy

In case participants permanently discontinue study drug treatment during this study, further therapy is at the discretion of the investigator.

6.8 Prior and concomitant therapy

A summary of the prohibited prior and concomitant therapy, outlined in the exclusion criteria (Section 5.1.2), is provided in Table 6-1.

Table 6-1 Prohibited prior and concomitant therapy

Therapy	Timeframe
Nitrates or NO donors (such as amyl nitrate) in any form, including topical; phosphodiesterase (PDE) 5 (PDE5) inhibitors (such as sildenafil, tadalafil, vardenafil); and nonspecific PDE inhibitors (theophylline, dipyridamole)	Concomitant therapy with study drug

In addition, caution is advised during the intake of any of the following concomitant medications:

- The concomitant use of riociguat with strong multi-pathway cytochrome P450 (CYP) and P-glycoprotein 1 (P-gp)/breast cancer resistance protein inhibitors such as azole antimycotics (e.g., ketoconazole, itraconazole) or human immunodeficiency virus protease inhibitors (e.g., ritonavir) is not recommended, due to the pronounced increase in riociguat exposure.
- The concomitant use of riociguat with strong CYP1A1 (CYP family 1, subfamily A, polypeptide 1) inhibitors, such as the tyrosine kinase inhibitor erlotinib, and strong P-gp inhibitors, such as the immunosuppressive agent cyclosporine A, may increase riociguat exposure (see section 'interaction with other medicinal products and other forms of interaction'). These drugs should be used with caution. Blood pressure should be monitored and dose reduction of riociguat considered.

Other medications considerations

- Pre- and concomitant treatment with the proton pump inhibitor omeprazole (40 mg once daily) reduced riociguat mean area under the plasma concentration versus time curve (AUC) by 26% and mean maximum plasma concentration (C_{max}) by 35%. This is not

considered clinically relevant.

- Co-administration of the antacid aluminum hydroxide / magnesium hydroxide reduced riociguat mean AUC by 34% and mean C_{max} by 56%. Antacids should be taken at least 1 hour after riociguat.

7. Procedures and variables

7.1 Schedule of events

The study will be divided into the following phases: screening phase, double-blind treatment phase, and open-label extension phase. Please refer to Section 7.1.1 for the schedule of events.

Figure 7.1 Flow diagram of Double Blind to Open Label Transition

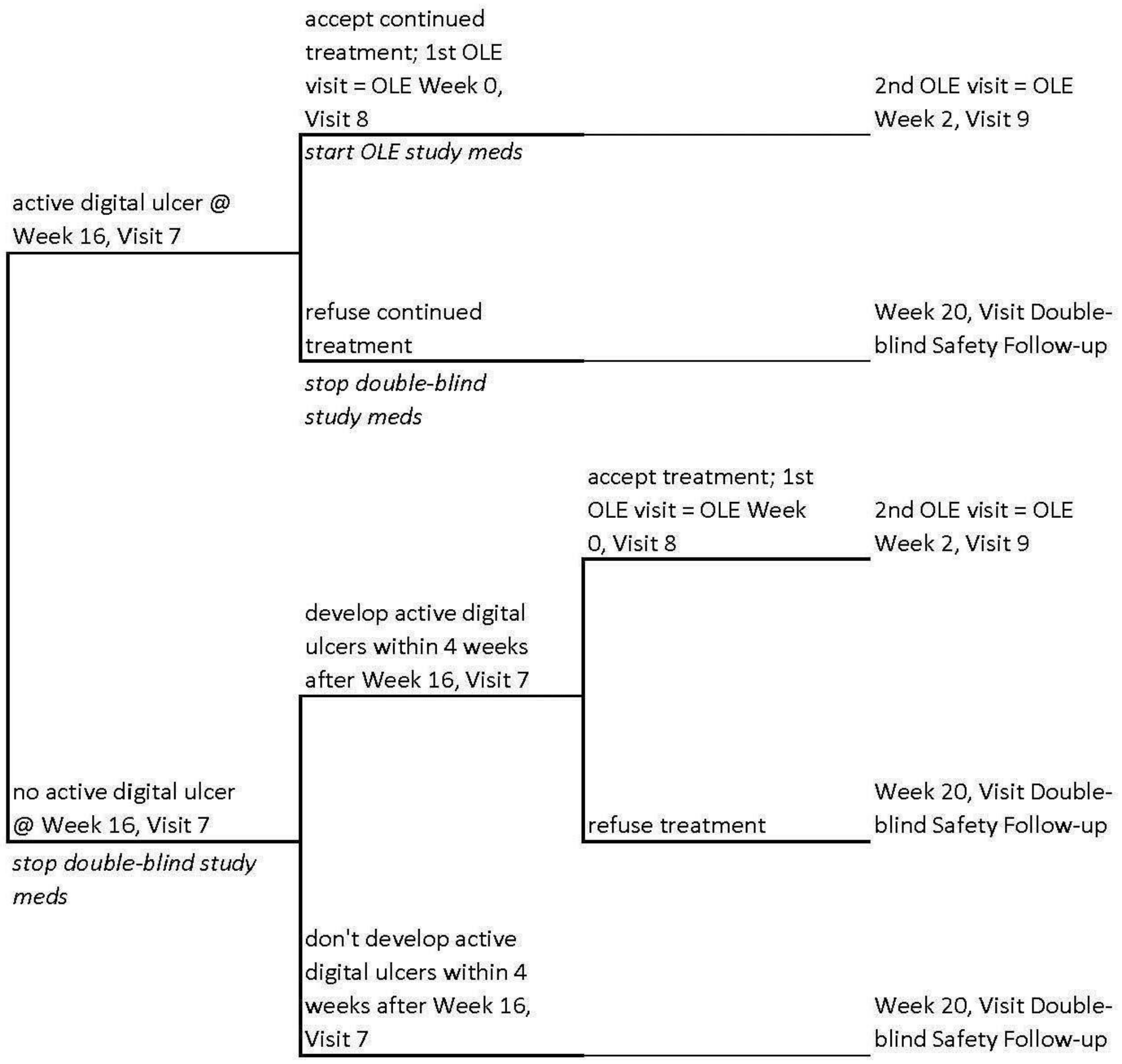

7.1.1 Schedule of Events

**Study Period: Double-Blind Phase**		**Dose Titration Period**						**Stable Dosing Period**		**Safety [h] Follow Up**
**Study Visit**	**Screening** Visit 0	**Baseline** Visit 1	**Week 2** Visit 2	**Week 4** Visit 3	**Week 6** Visit 4	**Week 8** Visit 5	**UNSH**[f]	**Week 12** Visit 6	**Week 16**[g] Visit 7	**Week 20** Visit 8
**Window (days):**	14	+4	+/-4	+/-4	+/-4	+/-4		+/-4	+/-4	+/-4
**Type of Contact:**	**Office**	**Office**	**Office**	**Office**	**Office**	**Office**	**Office**	**Office**	**Office**	**Office/Phone Call**
Informed consent	X									
Eligibility assessment	X	X								
Demographics, including smoking & alcohol history	X									
Complete medical history	X									
Prior/Concomitant therapy	X	X	X	X	X	X		X	X	X
Vitals	X	X	X	X	X	X	X	X	X	X
Physical examination	X			X			X		X	X
12-lead ECG	X					X			X	
Hematology, serum chemistry[a]	X					X			X	
Urine pregnancy test (women of childbearing potential)	X	X		X		X		X	X	
Hand x-rays for calcinosis (*not required at Screening if performed in prior 6 months*)	X									
Physician's Global Assessment		X							X	
Patient's Global Assessment		X							X	
PROMIS -29		X							X	
HAQ-DI/SHAQ		X							X	
HDISS-DU		X							X	
Raynaud's phenomenon diary	X[b]	X[c]						X[b]	X[c]	
Digital Ulcer Assessment	X	X	X	X	X	X		X	X	X
Digital photo of cardinal ulcer	X								X	
Dispense study medication		X	X	X	X	X	X	X	X[d]	
Assess for Adverse Events		X	X	X	X	X	X	X	X	X
Plasma Biomarkers[e]		X							X	

a	Participants with eGFR 15-29 ml/min or other laboratory abnormalities per physician judgment can repeat labs once during the screening period
b	Dispense 7-day diary;
c	Collect 7-day diary;
d	Open-Label study drug will be dispensed at this visit if participant is continuing to the OLE phase of the study. If a participant is exiting the study, all study drug should be returned at this visit and no more will be dispensed.
e	10 mL for biomarkers: VEGF, tPA, sE-Selectin, BFGF, VCAM-1, ICAM
f	This visit is for participants who experience toleration issues and return to clinic for assessment and dispense a lower dose.
g	Complete these visit assessment for any participants who terminate/withdrawal from the study prior to visit 7.
h	Complete this visit for any subject who does not enter the OLE. This can be a phone call if there are no new or ongoing adverse events attributable to the study medication and no digital ulcers to assess.

**Study Period: Open-Label Phase**	**Dose Titration Period**					**Stable Dosing Period**		
**Study Visit**	**OLE Week 0**[a] Visit 8	**OLE Week 2** Visit 9	**OLE Week 4** Visit 10	**OLE Week 6** Visit 11	**OLE Week 8** Visit 12	**OLE Week 12** Visit 13	**OLE Week 16/EOS**[c] Visit 14	**UNSH**[d]
**Window:**	+4	+/-4	+/-4	+/-4	+/-4	+/-4	+/-4	
**Type of Contact:**	**Office**	**Office**	**Office**	**Office**	**Office**	**Office**	**Office**	**Office**
Concomitant therapy	X	X	X	X	X	X	X	
Vitals	X	X	X	X	X	X	X	X
Physical examination	X[b]						X	X
12-lead ECG	X[b]	X					X	
Hematology, serum chemistry	X[b]				X		X	
Urine pregnancy test (women of childbearing potential)	X		X		X	X	X	
Physician's Global Assessment	X						X	
Patient's Global Assessment	X						X	
PROMIS -29	X						X	
HAQ-DI/SHAQ	X						X	
HDISS-DU	X						X	
Digital Ulcer Assessment	X	X	X	X	X	X	X	
Dispense study medication	X	X	X	X	X	X		X
Assess for Adverse Events	X	X	X	X	X	X	X	X

a Visit 8 would be performed for a participant who completed the double blind week 16 without DU and then returned to the clinic within the protocol timeline parameters with a new DU and wants to continue onto the OLE phase. For those participants who had active DU(s) at end of double blind and proceeded into the OLE immediately Visit 7/Visit 8 run concurrently (Visit 7→ Visit 9)

b The safety labs, ECG and physical exam do not need to be repeated at visit 8 if the participant returns with a DU within 7 days of completing the Visit 7.

c Complete this visit as an Early Termination/End of Study visit for any participant who ends the study prior to OLE Week 16/Visit 14.

d his visit is for participants who experience toleration issues and return to clinic for assessment and dispense a lower dose.

7.1.2 Timing of assessments

If not stated otherwise, all assessments and procedures will be performed by or under the supervision of an investigator.
For timing of assessments and procedures, please refer to Section 7.

7.1.2.1 Visit 0 – Screening

Screening evaluations will be performed only after the participant has provided written informed consent. The following evaluations will be performed and information obtained up to 14 days before randomization and the start of study drug treatment:

- Participant information and obtaining of written informed consent
- Eligibility: Assessment of inclusion and exclusion criteria (see Section 5.1)
- Provide written information on local wound care
- Take digital picture of the cardinal ulcer
- Demographic data, including sex, race, ethnic group, year of birth, smoking history and alcohol consumption
- Medical and surgical history
- Prior and concomitant therapy
- Physical examination, including height and weight
- Vital signs (blood pressure and heart rate). Blood pressure and heart rate will be measured at all visits in a sitting position after the subject has been at rest for at least 5 minutes. The same arm is always used for these measurements (See Section 7.6.4)
- Digital ulcer assessment (see Section 7.6.5)
- Provide 1-week diary to participant which they will complete daily for 7 consecutive days prior to Visit 1 to document Raynaud attacks (see Section 7.6.7)
- Pregnancy test (urine) for all women of childbearing potential (see Section 7.6.1)
- A standard 12-lead ECG will be obtained with the participant in the supine position after resting for at least 5 minutes
- Hand x-rays for calcinosis
- Blood samples for hematology, serum chemistry

7.1.2.2 Visit 1 – Baseline (Day 0, Week 0) – Double-Blind Treatment Phase

The following assessments will be performed at the Baseline visit (Day 0 of study drug treatment):

<u>Before intake of study medication</u>

- Reconfirmation of eligibility
- Discuss adherence with the local wound care
- Take digital picture of the cardinal ulcer, if not done at screening visit
- Patient reported outcomes (patient global assessment, PROMIS-29, and HAQ- D1/SHAQ and HDISS-DU) and physician global assessment
- Vital signs (blood pressure and heart rate). Blood pressure and heart rate will be

measured at all visits in a sitting position after the participant has been at rest for at least 5 minutes. The same arm is always used for these measurements
- Digital ulcer assessment
- Assessment of Raynaud attacks – participant to return completed diary
- Blood plasma sample for biomarkers to be obtained before the start of study drug treatment (see Section 7.6.3)
- Pregnancy test (urine) for all women of childbearing potential (see Section 7.6.1)
- Recording and assessment of AEs (see Section 7.5.1.3)
- Concomitant therapy
- Randomization to study drug treatment with either riociguat or placebo
- Dispense study drug

7.1.2.3 Visits 2 through 7 – Dose titration/Stable dosing periods (Double-blind treatment phase)

At these study visits, the participant should attend the clinic without having taken the morning dose of study medication.

During the dose titration period and stable dosing periods of the double-blind treatment phase (Visits 2 to 7), the following assessments will be performed according to the schedule of events (Section 7.1.1):

- Physical examination at Visits 3 and 7, including weight
- Vital signs (blood pressure and heart rate) at every visit. Blood pressure and heart rate will be measured at all visits in a sitting position after the participant has been at rest for at least 5 minutes. The same arm is always used for these measurements
- Discuss adherence with the local wound care at every visit
- Take digital picture of the cardinal ulcer at week 7
- Digital ulcer assessment at all visits
- Recording and assessment of AEs at all visits
- Pregnancy test (urine) for all women of childbearing potential at Visits 3, 5, 6, and 7
- A standard 12-lead ECG will be obtained with the participant in the supine position after resting for at least 5 minutes at Visits 5 and 7
- Hematology and serum chemistry at Visits 5 and 7
- Patient reported outcomes (patient global assessment, PROMIS-29, HAQ- DI/SHAQ and HDISS-DU) and physician global assessment at Visit 7.
- Assessment of Raynaud's attacks – participant to be provided 1-week diary at Visit 6 and returned on Visit 7. <u>Since the diary is only for 1 week, participant can do it for one continuous week between the 2 visits and bring it back at Visit 7.</u>
- Concomitant therapy at all visits
- Dispensation of study drug (At Visit 7 if participant has active or painful indeterminate DU, Open Label drug will be dispensed if participant consents to continued participation in the study.)** Important note: Participants should not take the last dose of randomized drug the night prior to coming to the clinic for visit 7.
- Blood plasma sample for biomarkers to be obtained before the start of study drug

treatment (see Section 7.6.3) at Visit 7

7.1.2.4 Early Termination Visit during Double Blind

In the case of an early termination/withdrawal during double-blind period, follow the schedule of events for Visit 7. The only exception is that Raynaud's diary is not collected if it was not dispensed at least 1 week prior to early termination visit.

7.1.2.5 Unscheduled visit

Participants can be brought into the clinic for an unscheduled (UNSCH) visit should they experience dose toleration issues or other treatment related adverse events in between their regularly-scheduled visits.

During an UNSCH visit, vitals, physical exam, and assessment for AE's should occur. Should a dose reduction be warranted the participant should return the previously dispensed medication bottles. The site can then dispense a lower dose through the drug dispensing system.

7.1.2.6 Visit 8 Safety Follow-up for double-blind period or start of Open-label

If the participant does not qualify for or chooses not to participate in the open-label phase of the study at visit 7, they will return in 4 weeks for visit 8 which is intended to be a Safety Follow-up visit. However, if at this time (visit 8), they have a DU and choose to enter the open-label phase, this visit is considered to be the start of the Open-Label phase (OLE Week 0) and they can receive study medication at this visit. If at this time (visit 8), they don't have DU, then this visit is the Week 20 Safety Follow-up (Visit 8). If a participant does not have any adverse events that are attributable to the study medication [as assessed by the physician] the week 20 safety follow-up can be performed over the phone, if the participant chooses, in order to minimize their burden.

Week 20 Safety Follow-up (Visit 8) Procedures:

- Concomitant therapy
- Physical examination, including height and weight (if in-person visit)
- Vital signs (blood pressure and heart rate). (if in-person visit)
- Discuss adherence with the local wound care if digital ulcers present
- Digital ulcer assessment (if in-person visit)
- Recording and assessment of AEs

Open-Label phase (OLE Week 0) Visit 8

- Physical examination including weight (not required if the participant returns with a DU within 7 days of completing the Visit 7)
- Vital signs (blood pressure and heart rate).
- Discuss adherence with the local wound care
- Digital ulcer assessment

- Recording and assessment of AEs
- Pregnancy test (urine) for all women of childbearing potential
- A standard 12-lead ECG, (not required if the participant returns with a DU within 7 days of completing the Visit 7)
- Hematology and serum chemistry (not required if the participant returns with a DU within 7 days of completing the Visit 7)

7.1.2.7 Visits 9 through 14 – Dose-titration phase/ Stable dosing periods (Open label extension phase)

- Concomitant therapy at every visit
- Physical examination, including height and weight (Visit 14)
- Vital signs (blood pressure and heart rate) at every visit. Blood pressure and heart rate will be measured at all visits in a sitting position after the participant has been at rest for at least 5 minutes. The same arm is always used for these measurements
- Discuss adherence with the local wound care at every visit
- Digital ulcer assessment at all visits
- Patient reported outcomes (patient global assessment, PROMIS-29, and HAQ-DI/SHAQ) and physician global assessment at Visit 14.
- Hematology and blood chemistry Visits 12, and 14
- Pregnancy test (urine) for all women of childbearing potential at Visits 10, 12, 13, and 14.
- A standard 12-lead ECG will be obtained with the participant in the supine position after resting for at least 5 minutes at Visits 9 and 14.
- Recording and assessment of AEs at every visit
- Dispensation of open-label riociguat study drug at every visit up to and including Visit 13
- Visit 14 will be the end of study visit.

7.1.2.8 Early Termination Visit during Open Label Extension

In the case of an early termination/withdrawal during OLE, follow the schedule of events for Visit 14.

7.2 Population characteristics

7.2.1 Demographic

The following demographic data will be recorded:

- Date of birth (age)
- Sex
- Race and Ethnicity
- Alcohol consumption
- Smoking History

7.2.2. Medical history

Medical history findings (i.e., previous diagnoses, diseases or surgeries) meeting all criteria listed below will be collected:

- Pertaining to the study indication
- Started before signing of the informed consent
- Considered relevant to the study
- Medical history related to concomitant therapy

7.3 Efficacy

Primary efficacy outcome measure:

- Change from baseline to end of the double-blind study treatment phase in digital ulcer net burden
    - Digital ulcer net burden is defined as the total number of “active” or “indeterminate” digital ulcers at that assessment.
    - Should be assessed as any ulcer including skin area distal to Metacarpophalangeal (MCP) joint..

Secondary efficacy measures during double-blind period:

- Healing of the cardinal DU
- Time to healing of the cardinal DU
- Time to healing of all DUs
- Development of DU during the trial
- Development/healing of ulcers over DIP, PIP, MCPs and elbows.
- Improvement of Raynaud’s phenomenon
    - Raynaud’s condition score
    - Number of Raynaud’s attacks/day
    - Patient and physician assessment of RP; pain, numbness, and tingling during an RP attack; and duration of attacks
- Patient’s and physician’s global assessment on a Likert scale
- HRQOL using PROMIS-29
- Physical function as assessed by HAQ-DI, and HDISS-DU
- Visual analog scales from scleroderma-health assessment questionnaire (SHAQ) assessing burden of digital ulcers, Raynaud’s disease, gastrointestinal involvement, breathing, and overall disease
- Digital ischemia requiring intravenous prostacyclin or digital gangrene or amputation
- Vascular biomarkers in the plasma (VEGF, tPA, sE-Selectin, BFGF, VCAM-1, ICAM)

Secondary efficacy measures during OLE period:

- Healing of the cardinal DU

- Development of DU during the trial
- Development/healing of ulcers over DIP, PIP, MCPs and elbows.
- Patient's and physician's global assessment on a Likert scale
- HRQOL using PROMIS-29
- Physical function as assessed by HAQ-DI and HDISS-DU
- Visual analog scales from scleroderma-health assessment questionnaire (SHAQ) assessing burden of digital ulcers, Raynaud's disease, gastrointestinal involvement, breathing, and overall disease
- Digital ischemia requiring intravenous prostacyclin or digital gangrene or amputation

7.4 Pharmacokinetics / pharmacodynamics

Pharmacokinetics will not be performed in this study.

7.5 Safety

7.5.1 Adverse events

7.5.1.1 Definitions

Definition of adverse event (AE)

In a clinical study, an AE is any untoward medical occurrence (i.e., any unfavorable and unintended sign [including abnormal laboratory findings], symptom or disease) in a patient or clinical investigation participant after providing written informed consent for participation in the study. Therefore, an AE may or may not be temporally or causally associated with the use of a medicinal (investigational) product.

A surgical procedure that was planned prior to the start of the study by any physician treating the participant should not be recorded as AE (however, the condition for which the surgery is required may be an AE).

In the following differentiation between medical history and AEs, the term "condition" may include abnormal physical examination findings, symptoms, diseases, laboratory results, and ECG findings.

- Conditions that started before signing of informed consent and for which no symptoms or treatment are present until signing of informed consent are recorded as medical history (e.g., seasonal allergy without acute complaints).
- Conditions that started before signing of informed consent and for which symptoms or treatment are present after signing of informed consent, at *unchanged intensity*, are recorded as medical history (e.g., allergic pollinosis).
- Conditions that started or deteriorated after signing of informed consent will be documented as AEs.

Definition of serious adverse event (SAE)

An SAE is classified as any untoward medical occurrence that, at any dose, meets any of the following criteria (a – f):

a. Results in death

b. Is life-threatening

   The term 'life-threatening' in the definition refers to an event in which the participant was at risk of death at the time of the event, it does not refer to an event which hypothetically might have caused death if it were more severe.

c. Requires inpatient hospitalization or prolongation of existing hospitalization

   A hospitalization or prolongation of hospitalization will not be regarded as an SAE if at least one of the following exceptions is met:

   - The admission results in a hospital stay of less than 12 hours
   - The admission is pre-planned (i.e., elective or scheduled surgery arranged prior to the start of the study)
   - The admission is not associated with an AE (e.g., social hospitalization for purposes of respite care).

   However, it should be noted that invasive treatment during any hospitalization may fulfill the criterion of 'medically important' and as such may be reportable as an SAE dependent on clinical judgment. In addition, where local regulatory authorities specifically require a more stringent definition, the local regulation takes precedence.

d. Results in persistent or significant disability / incapacity

   Disability means a substantial disruption of a person's ability to conduct normal life's functions.

e. Is a congenital anomaly / birth defect

f. Is another medically important serious event as judged by the investigator

7.5.1.2 Classifications for adverse event assessment

All AEs will be assessed and documented by the investigator according to the categories detailed below.

7.5.1.2.1 Seriousness

For each AE, the seriousness must be determined according to the criteria given in Section 7.5.1.1 .

7.5.1.2.2 Severity

The severity of an AE is classified according to the following categories:

- Grade 1 Mild
- Grade 2 Moderate
- Grade 3 Severe
- Grade 4 Very Severe
- Grade 5 Death

7.5.1.2.3 Causal relationship

The assessment of the causal relationship between an AE and the administration of treatment is a clinical decision based on all available information at the time of the completion of the eCRF.

The assessment is based on the question whether there was a "reasonable causal relationship" to the study treatment in question.

Possible answers are "Related" or "Not Related" An assessment of "Not Related" would include:

1. The existence of a clear alternative explanation, e.g., mechanical bleeding at surgical site.

or

2. Non-plausibility, e.g., the participant is struck by an automobile when there is no indication that the drug caused disorientation that may have caused the event; cancer developing a few days after the first drug administration.

An assessment of "Related" indicates that there is a reasonable suspicion that the AE is associated with the use of the study treatment.

Important factors to be considered in assessing the relationship of the AE to study treatment include:

- The temporal sequence from drug administration:
  The event should occur after the drug is given. The length of time from drug exposure to event should be evaluated in the clinical context of the event.
- Recovery on drug discontinuation (de-challenge), recurrence on drug re-introduction (re-challenge):
  Participant's response after de-challenge or participants response after re-challenge should be considered in the view of the usual clinical course of the event in question.
- Underlying, concomitant, intercurrent diseases:
  Each event should be evaluated in the context of the natural history and course of the disease being treated and any other disease the participant may have.
- Concomitant medication or treatment:

The other drugs the participant is taking or the treatment the participant receives should be examined to determine whether any of them may be suspected to cause the event in question.

- The pharmacology and pharmacokinetics of the study treatment:
  The pharmacokinetic properties (absorption, distribution, metabolism and excretion) of the study treatment, coupled with the individual participant's pharmacodynamics should be considered.

Causal relationship to protocol-required procedure(s)

The assessment of a possible causal relationship between the AE and protocol-required procedure(s) is based on the question whether there was a "reasonable causal relationship" to protocol-required procedure(s).

Possible answers are "yes" or "no"

7.5.1.2.4 Action taken with study treatment

Any action on study treatment to resolve the AE is to be documented using the categories listed below.

- Drug withdrawn
- Drug interrupted
- Dose reduced
- Dose not changed
- Not applicable
- Unknown

7.5.1.2.5 Other specific treatment(s) of adverse events

- None
- Remedial drug therapy
- Other

7.5.1.2.6 Outcome

The outcome of the AE is to be documented as follows:

- Recovered/resolved without sequelae
- Recovered/resolved with sequelae
- Not recovered/not resolved (ongoing)
- Fatal
- Unknown

7.5.1.3 Assessments and documentation of adverse events

The investigator has the obligation to report AEs. All non-serious events will be assessed and recorded during the specified observational phase (from signing the informed consent form up to 30 [+5] days after last study medication intake), whether believed to be related or unrelated to the treatment. AE forms will be included in the eCRFs. The record will include clinical symptoms or final diagnosis when available, date of appearance, duration, severity and relationship to treatment. A record will also be kept of the action taken and the follow-up until resolution of the AE.

7.5.1.4 Reporting of serious adverse events

Reporting of SAEs to the FDA

The sponsor-investigator will report serious adverse event to the FDA according to the regulations found at 21 CFR 312.32.

Notification of the IECs / IRBs

Notification of the IECs / IRBs about all relevant events (e.g., SAEs, suspected, unexpected, serious adverse reactions [SUSARs]) will be performed by the investigator according to all applicable regulations.

Sponsor's notification of the investigational site

The sponsor will inform all investigational sites about reported relevant events (e.g., SUSARs) according to all applicable regulations.

Sponsor Investigator reporting to Bayer

All serious adverse events should be reported to the DCC who is responsible for reporting all serious adverse events that are unexpected and related to study medication to Bayer, the manufacturer of riociguat, within 24 hours.

7.5.1.5 Expected adverse events

For this study, the applicable reference document is the most current version of the package insert.

The expectedness of AEs will be determined by the sponsor according to the applicable reference document and according to all local regulations.

7.5.1.6 Adverse events of special safety interest

Symptomatic hypotension and serious hemoptysis have been defined as AEs of special interest and must be documented accordingly in the corresponding AE eCRF. Events of symptomatic hypotension and non-serious hemoptysis should not automatically be upgraded by the reporting Investigator to serious. Declaration of an event as serious should only occur when 1 or more of

the serious criteria (as defined in Section 7.5.1.1) are applicable.

7.5.1.7 Overdose of Study Medication

In case of overdose, standard supportive measures should be adopted as required. In case of pronounced hypotension, active cardiovascular support may be required. If symptoms develop after the investigational drug has been administered, any therapy that becomes necessary has to be guided by the predominant symptoms. Participants must remain under medical supervision until all relevant adverse effects have resolved in the event of overdose; participant continuation remains at the discretion of the investigator.

7.5.2 Pregnancies

Riociguat is a category X drug and does come with a black box warning for embryo-fetal toxicity. Riociguat should not be administered to a pregnant female because it may cause fetal harm. For females of reproductive potential, pregnancy should be avoided before the start of riociguat treatment, during treatment, and for 1 month after stopping treatment. Pregnancy will be monitored with monthly urine pregnancy tests during treatment. Acceptable methods of contraception should be used to prevent pregnancy during treatment and for one month after stopping treatment.

The investigator must report to the sponsor any pregnancy occurring in a study participant during the participant's participation in this study. The report should be submitted within the same timelines as an SAE, although a pregnancy per se is not considered an SAE. Any pregnancies should be reported to the DCC who is responsible for reporting to Bayer, the manufacturer of riociguat.

For a study participant, the outcome of the pregnancy should be followed up carefully, and any outcome of the mother or the child should be reported.

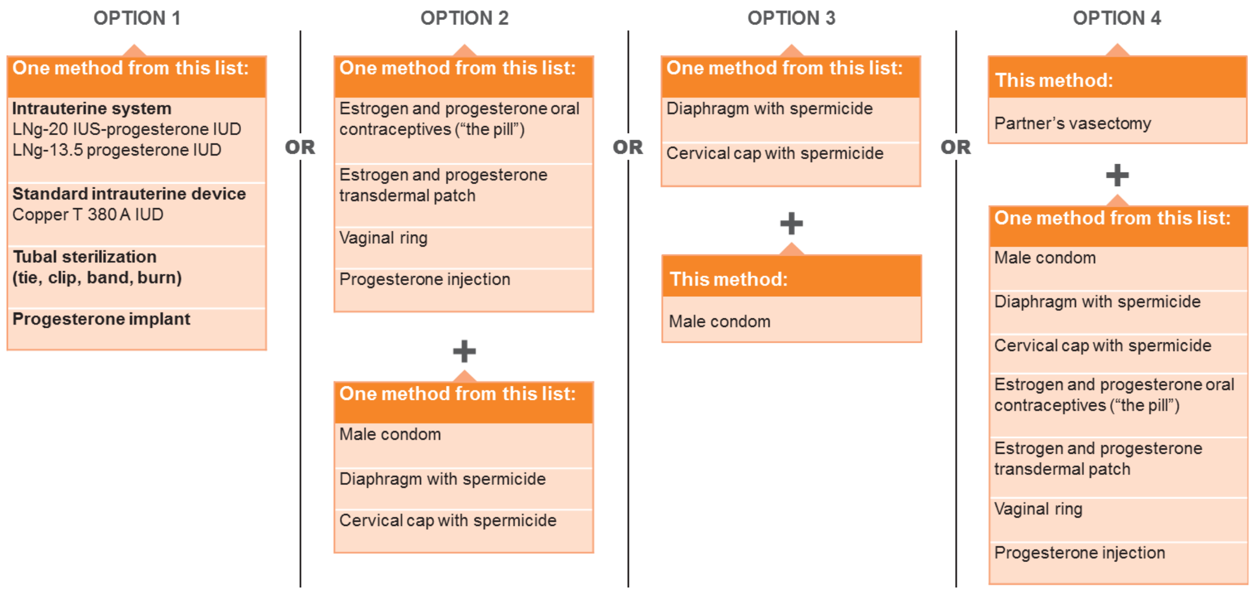

7.6 Other procedures and variables

7.6.1 Pregnancy testing

Pregnancy testing is to be performed at the Screening Visit, Visit 1 (Day 0; urine) and in 4-weekly intervals thereafter (urine) until one month after the participant stops intake of study drug. The results are to be documented in the eCRF and in the patient file.

In the event of pregnancy, study medication must be stopped and a referral to a gynecologist for confirmation of pregnancy must be organized as soon as possible. In addition, the pregnancy must be reported to the sponsor as described in Section 7.5.2 and further consequences with regard to ongoing participation in the study must be discussed with the participant. In the event uncertainties about a pregnancy test outcome exist, the participant should contact the site immediately to discuss further steps (e.g., exclusion of pregnancy by serum pregnancy test).

Women of childbearing potential and non-vasectomized male participants must agree to use adequate contraception when sexually active.

'Adequate contraception' is defined as any combination of at least 2 effective methods of birth control, of which at least one is a physical barrier (e.g., condoms with hormonal contraception or implants or combined oral contraceptives, certain intrauterine devices).

7.6.2 Laboratory parameters

The laboratory parameters will be analyzed in a local laboratory and reported on the eCRF.

7.6.3 Exploratory Biomarkers

Exploratory vascular biomarkers will be evaluated at Visit 1 (Day 0) and Visit 7 (Week 16). Plasma for determination of VEGF, tPA, sE-Selectin, BFGF, VCAM-1, and ICAM will be collected and shipped to the University of Michigan laboratory. Details on collection, processing and storage are described in the Manual of Operations.

7.6.4 Blood pressure and heart rate measurement

Blood pressure and heart rate will be measured at all visits in a sitting position after the participant has been at rest for 5 minutes. The same arm is always used for these measurements.

Methodology: Noninvasive measurement preferably with a validated electronic device in accordance with published guidelines (e.g., American Heart Association Recommendations for Blood Pressure Measurement in Humans and Experimental Animals).

7.6.5 Digital ulcer net burden

Digital ulcers are defined as a full thickness skin lesion with loss of epithelium. Ulcers should be > 3mm in maximal diameter. Healing is defined by re-epithelialization with loss of pain and exudate. Pitting scars and hyperkeratotic lesions are always excluded. Also, eschar is not considered as a DU.

Digital ulcer net burden will be assessed by the following methods:

- Ulcer count – Ulcer assessment will ideally be performed by the same physician at every visit:
    - Total ulcer counts distal to MCP joint (this can include both distal ulcer [defined as distal to PIP joint] and proximal ulcer [defined between MCP and PIP joint}
    - Distal (fingertip) ulcer counts: any ulcer including skin area distal to proximal interphalangeal (PIP) joint
- Ulcer net burden: Ulcer net burden is defined as the total number of “active” or indeterminate digital ulcers at that assessment. Any ulcerfrom the Metacarpophalangeal (MCP) joint to distal phalanges on the dorsal and palmar (volar) surface of the hand, (including the digital tip) and the elbows will be assessed.
- Visual analog score for patient-reported severity of digital ulcers as part of the SHAQ (see section 7.6.8)
- Non-ischemic ulcers over the dorsum of DIP, PIP, MCP and over elbows will also be evaluated for healing as secondary outcomes

7.6.6 Digital Gangrene

Digital gangrene will be recorded as a separate event and may co-exist with digital ulcers that affect the same digit.

Gangrene is defined as diffuse necrosis (pathologic death of deeper, e.g. subcutaneous, structures) due to obstruction, loss, or diminution of blood supply; it is of black color and painful, and may be localized to parts of the finger or involve an entire finger. It may be wet or dry, reflecting a degree of adjacent tissue perfusion, time course of necrosis and presence or type of associated secondary infection.

7.6.7 Raynaud’s attacks assessment

Raynaud’s attacks will be assessed using the composite of the following 6 individual outcome measures in order to minimize the measurement variability and placebo response(7): Raynaud’s condition score, patient assessment of Raynaud’s phenomenon, physician assessment of Raynaud’s phenomenon, attack symptoms, duration of attacks, and number of attacks per day.

The Raynaud’s condition score is a daily patient assessment of Raynaud’s phenomenon activity using a 0-10 ordinal scale. It incorporates the cumulative frequency, duration, severity and impact of Raynaud’s phenomenon attacks, reflecting the overall degree that Raynaud’s has affected use of the participant’s hands. The Raynaud’s condition score, along with details of the frequency and duration of Raynaud’s attacks, will be incorporated into the daily diary that participants will be asked to complete for 1 week (7 days) at the time points shown below.

The patient and physician assessment assesses the severity of Raynaud's phenomenon in the past week using a 0-100 VAS.

Frequency: Daily diary to be completed by participant for 7 consecutive days prior to study visits at Screening and Week 12.

7.6.8 Patient-Reported Outcomes (PROs) / Health-Related Quality of Life (HRQoL) questionnaires

Three patient-reported outcomes (PROs)—the HAQ-DI/SHAQ, HDISS-DU and PROMIS-29—will be completed by all participants in the study.

Scleroderma Health Assessment Questionnaire (HAQ-DI/SHAQ)

The HAQ-DI consists of 8 domains from the Health Assessment Questionnaire disability index, a HRQoL instrument that measures self-reported function in 8 domains of activity in 20 weighted responses and a VAS of pain experienced in the past week. It additionally measures 5 domains specific to scleroderma using a continuous VAS: Raynaud's phenomenon, digital tip ulcers, lung symptoms, gastrointestinal symptoms, and a global patient assessment. The VAS subscales of the SHAQ were shown to be significantly correlated with objective parameters and were responsive to change in a cohort and in a Raynaud's phenomenon trial in SSc. The SHAQ requires approximately 5 minutes to complete.

Hand Disability in Systemic Schlerosis-DU (HDISS-DU™)

The HDISS-DU™ was developed in accordance with the 2009 guidance from the US Food and Drug Administration.The concepts and items comprising the HDISS-DU were developed based on an initial phase of interviews of digital ulcer (DU) patients that included concept elicitation and cognitive debriefing on the Cochin Hand Function Scale (an 18-item functional disability questionnaire that uses tasks of daily living to assess function), and a second phase of patient cognitive debriefing interviews on a revised instrument. Results from the cognitive debriefing interviews suggest that the draft HDISS-DU is a comprehensive measure assessing the impact of DUs on hand functioning that asks patients to rate their ability to complete common activities over the past 7 days. The psychometric evaluation, including final validation of the HDISS-DU and derivation of a scoring algorithm, is under development based on blinded data from the DUAL studies. The HDISS-DU requires approximately 5 minutes to complete.

Patient-Reported Outcomes Measurement Information System (PROMIS)-29

The PROMIS-29 is a validated instrument to measure the health status of SSc patients, demonstrating moderate to high correlation with other instruments validated in SSc, including the SF-36 physical component score and HAQ-DI. It incorporates 7 core domains from the PROMIS questionnaire, which specifically relate to physical, mental, and social health aspects of chronic illness: pain, fatigue, depression, anxiety, sleep, and physical function, as well as one 11-point rating scale for pain intensity. It contains 8 items with 29 weighted responses in total,

and requires approximately 5 minutes to complete.

7.6.9 Participant and Physician Global Assessment

A global assessment for patient and physician will be obtained.

The participant assessment represents the patient's assessment of the patient's global scleroderma on a 0-10 Likert scale. "On a scale of 0-10, how was your overall health in the last week? 0=Excellent; 10=Extremely Poor. The physician global assessment represents the physician's assessment of the patient's current disease activity on a 0-10 Likert scale. "On a scale of 0-10, how was your patient's overall health in the last week? 0=Excellent; 10=Extremely Poor". Both assessments are made at baseline and Week 16, OLE Week 0 and OLE Week 16.

8. Statistical methods and determination of sample size

8.1 General considerations

This section presents a summary of the planned statistical analyses. A statistical analysis plan (SAP) will be written for the study that contains detailed descriptions of the analyses to be performed. The SAP will be finalized prior to unblinding of the data.

Continuous variables will be summarized using descriptive statistics including n, mean, median, standard deviation, range (e.g., minimum and maximum). Qualitative variables will be summarized using counts and percentages. Summaries will be provided by treatment group and overall. Graphical methods will be heavily used in this pilot study to assess the pattern of response over time for key variables and to assess the relationships among variables. For most outcomes, separate analyses will be performed for the double-blind and open-label extension treatment periods.

Unless otherwise specified, statistical analyses will be performed using SAS Version 9 or higher. Where appropriate, statistical tests will be conducted at the 0.05 significance level (with no adjustments for multiplicity) using two-tailed tests and p-values will be reported.

Given the rare nature of SSc and the consequent small sample size for this pilot Phase IIa study, the statistical power of any comparisons is limited (i.e., there is sufficient power to detect only large treatment differences). As such the analysis will be largely descriptive in nature as the study is not powered to determine a statistical difference between riociguat vs. placebo. The p-values resulting from formal statistical tests will be interpreted from a hypothesis-generating, rather than a confirmatory framework.

8.2 Analysis sets

The main population for efficacy will be the modified intention-to-treat population (MITT), defined as all participants randomized, receiving at least one dose of treatment, and having at least one post-baseline efficacy assessment. Participants will be analyzed by assigned treatment. The primary endpoint and all secondary outcomes will be assessed using this analysis set.

The Per Protocol population (PP) will consist of all participants in the MITT population who did not have a major protocol violation, inclusive of violation of entry criteria. Participants in this population will be referenced as evaluable. Only the primary endpoint will be assessed using this analysis set (sensitivity analysis).

The Safety Population is defined as all participants who were randomized and received at least one dose of the study drug. The Safety Population will be used for all safety analyses. Participants will be analyzed by treatment received. If participants inadvertently receive both active drug and placebo, they will be included in the riociguat group.

Membership in the analysis populations will be determined before study unblinding.

8.3 Statistical and analytical plans

8.3.1 Demographic and other baseline characteristics

Demographic variables and baseline characteristics will be summarized by treatment group.

8.3.2 Efficacy

The primary endpoint is the mean change from baseline to end of the double-blind study treatment phase (Week 16) in the digital ulcer net burden. For the primary analysis, changes in digital ulcer net burden will be compared in the two treatment groups using an ANCOVA model with terms for treatment group and baseline digital ulcer net burden value. If the assumptions of this parametric model are not met, an alternative non-parametric model will be used. This model is based on the extension of the Wilcoxon rank-sum test to allow for covariate adjustment (9). This rank ANCOVA can provide additional power associated with baseline covariate adjustment, even when the outcome variable is not normally distributed 1(10).

Analysis for secondary outcome measures that are continuous will be performed using a similar approach as that for the primary endpoint. We will compare the change in each secondary outcome measure from baseline to week 16 between the two treatment groups using an ANCOVA model or its non-parametric counterpart if the model assumptions aren't met. Analyses of secondary outcomes measures that are discrete will be performed using Fisher's exact tests. Analyses of secondary outcome measure that are counts will be performed using Poisson regression.

8.3.3 Safety

Descriptive summary statistics for treatment-emergent adverse and serious adverse events will be reported. Adverse events will be grouped by body system and grade and will be tabulated as numbers and percentages; serious adverse events will be enumerated and described as appropriate. The total number of adverse events of each grade occurring in the two treatment groups by Week 16 will be compared using a Fisher's exact test. Poisson regression or comparable non-parametric methods will be used to compare the total number of serious adverse

events during the double-blind 16-week period and (separately) during the open-label extension 16-week period, by treatment group. Similar analyses will be conducted for treatment-emergent AEs and SAEs leading to discontinuation of study drug and of special safety interest.

The other safety outcomes will be presented with descriptive statistics.

The safety evaluation of laboratory data will include:

- Incidence rates of treatment-emergent laboratory values outside of normal range.
- Incidence rates of pre-specified laboratory data abnormalities.
- Descriptive analysis of continuous laboratory parameters, and their changes from baseline by treatment group and visit.

Descriptive analysis of vital signs, and their changes from baseline, will be performed by treatment group and visit.
For ECGs, the status pre-treatment and post-treatment will be tabulated. The incidence rates of treatment-emergent ECG abnormalities will be tabulated by treatment group. A descriptive analysis of continuous ECG parameters and their changes from baseline by treatment group and visit will also be presented.

8.4 Planned interim analyses

No formal interim analysis of the double-blind treatment phase is planned.

8.5 Determination of sample size

SSc is a rare disease. The planned sample size of 20 SSc participants is based on practical considerations to obtain preliminary estimates of the magnitude of treatment differences in efficacy and safety rather than a desired power for a pre-specified difference as would be necessary for a confirmatory study. However, with this proposed sample of 20 participants (10 riociguat and 10 placebo), we can calculate the magnitude of treatment differences (riociguat – placebo) for the primary efficacy endpoint – the change from baseline to end of double-blind treatment in digital ulcer net burden (a continuous endpoint), or safety outcomes – characterized by the proportion of participants who experience an AE. There would be 80% power to detect an effect size (mean treatment difference divided by standard deviation) of 1.253 or greater with a two-sided type I error of 5% in the primary endpoint, based on a two- sample t test. Given the pilot nature of this Phase IIa study, the difference between mean change in digital ulcer net burden between riociguat and placebo that can be detected with sufficient power is large. Similarly, we can calculate power for safety outcomes for this sample size: there is 81% power to detect treatment differences of 51%, assuming 40% of placebo-treated participants experience an AE based on a two-sample binomial test.

8.6 Data recording

It is the expectation of the sponsor that all data entered into the eCRF has source documentation available at the site. The site must implement processes to ensure this happens. A source

document checklist will be used at the site to identify the source data for all data points collected and the monitor will work with the site to complete this.

8.7 Monitoring

Given the enrollment period is dictated by seasonal considerations for DU incidence, we anticipate two monitoring visits per site during the course of the study. Once after the first subject has been randomized at the site and once after the study has closed to enrollment and all subjects have completed study visits. The frequency of monitoring visits may be adjusted throughout the life cycle of the study as study conditions and needs evolve. Additional visits can be scheduled at the request of the Study Team or DSMB.

The Clinical Research Monitor will ensure that:

- Data collected and entered into the database are verifiable against source documents for the randomized participants
- Appropriate consent is obtained for each participant prior to study procedures
- Safety and rights of participants are being protected
- Study is conducted in accordance with the currently approved protocol (including study treatment being used in accordance with the protocol)
- Study medication is properly dispensed and accounted for
- Any other study agreements, GCP, and all applicable regulatory requirements are met.

The investigator and the head of the medical institution (where applicable) agrees to allow the monitor direct access to all relevant documents.

8.8 Archiving

Essential documents shall be archived safely and securely in such a way that ensures that they are readily available upon authorities' request.

Participant (hospital) files will be archived according to local regulations and in accordance with the maximum period of time permitted by the hospital, institution or private practice. Where the archiving procedures do not meet the minimum timelines required by the sponsor, alternative arrangements must be made to ensure the availability of the source documents for the required period.

The investigator/institution notifies the sponsor if the archival arrangements change (eg, relocation or transfer of ownership).

The investigator site file is not to be destroyed without the sponsor's approval.

The contract with the investigator/institution will contain all regulations relevant for the study center.

9. Premature termination of the study

The sponsor (Dr. Khanna) has the right to close this study (or, if applicable, individual segments thereof [e.g., treatment arms; dose steps; centers]) at any time, which may be due but not limited to the following reasons:

- If risk-benefit ratio becomes unacceptable owing to, for example,
  - Safety findings from this study (e.g., SAEs)
  - Results of any interim analysis
  - Results of parallel clinical studies
  - Results of parallel animal studies (e.g., toxicity, teratogenicity, carcinogenicity or reproduction toxicity).
- If the study conduct (e.g., recruitment rate; drop-out rate; data quality; protocol compliance) does not suggest a proper completion of the trial within a reasonable time frame.

The investigator has the right to close his/her center at any time.

For any of the above closures, the following applies:

- Closures should occur only after consultation between involved parties. Final decision on the closure must be in writing.
- All affected institutions (e.g., IEC(s)/IRB(s); competent authority (-ies); study center; head of study center) must be informed as applicable according to local law.
- All study materials (except documentation that has to remain stored at site) must be returned to the sponsor. The investigator will retain all other documents until notification given by the sponsor for destruction.
- In case of a partial study closure, ongoing participants, including those in post study follow-up, must be taken care of in an ethical manner.

Details for individual participant's withdrawal can be found in Section 5.2.1.

10. Ethical and legal aspects

10.1 Ethical and legal conduct of the study

The procedures set out in this protocol, pertaining to the conduct, evaluation, and documentation of this study, are designed to ensure that the sponsor and investigator abide by GCP guidelines and under the guiding principles detailed in the Declaration of Helsinki. The study will also be carried out in keeping with applicable local law(s) and regulation(s).

Strict adherence to all specifications laid down in this protocol is required for all aspects of study conduct; the investigator may not modify or alter the procedures described in this protocol.

Investigators may implement a protocol change after discussing the details and getting IRB approval. Any deviations from the protocol must be explained and documented by the investigator.

Details on discontinuation of the entire study or parts thereof can be found in Section 9.

10.2 Compensation for health damage of participants / insurance

This is an investigator-initiated study. Therefore, compensation will be provided per local hospital policies or by participant's medical insurance. There is no compensation provided by the sponsor or Bayer.

11. Appendices

11.1 Child-Pugh Classification of Liver Disease

Factor	+1	+2	+3
**Bilirubin (mg/dL)**	< 2	2 – 3	> 3
**Albumin (g/dL)**	< 2.8	2.8 – 3.5	> 3.5
**INR**	< 1.7	1.7 – 2.3	> 2.3
**Ascites**	None	Mild	Moderate / Severe
**Encephalopathy**	None	Grade II – III or medically controlled	Grade III – IV or poorly controlled

Child-Pugh Class	A	B	C
**Points**	5 -6	7 – 9	10 - 15

INR = International Normalized Ratio.
Source: Pugh RN, Murray-Lyon IM, Dawson JL, Pietroni MC, Williams R. Transection of the oesophagus for bleeding oesophageal varices. Br J Surg 1973;60:646-649

11.2 Digital Ulcer Care Protocol

Digital Ulcer Care protocol:

Active ulcers:

Clean the ulcer once daily. This can be done by either soaking or gently washing the ulcer with sterile normal saline (this can be bought from any drug store). Allow the ulcer to air-dry or pat-dry using a sterile gauze.

Apply vitamin E oil once daily. This should be done by dropping the oil on to the sterile gauze which will be covering the wound. The gauze will then be placed over the ulcer, and secured by either 3M™ Coban (sold as breathable gentle tape), absolute waterproof tape (sold by Nexcare or local pharmacy brand) or a Band-Aid.

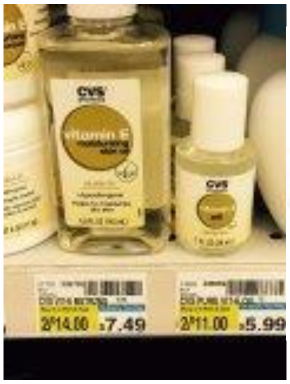

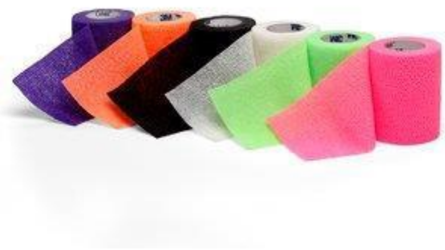
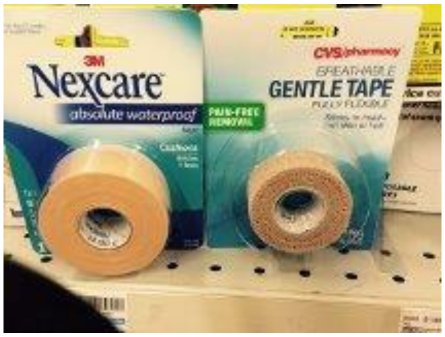

Dressings should be changed at least once daily. If the ulcer appears macerated the option of leaving the wound open at night may be considered.

Indeterminate ulcers with significant eschar/crust formation (no longer open):

Ulcers should continue to be cleaned once daily. AT this time either sterile normal saline or sterile water may be used by either soak or gentle washing.

When eschar/crust forms, hydrogen peroxide once daily should be used (to help remove eschar formation). The 3% hydrogen peroxide solution can be used by soaking or applying with a sterile q- tip, depending on the size of the ulcer. Add 1 table spoon of hydrogen peroxide solution in 5 table spoons of water (1:5 concentration).

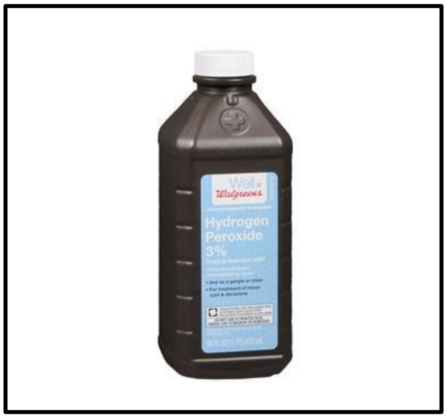

At this stage the ulcer may be kept clean and dry overnight. During activities a dressing of gauze and secured by either Coban, Nexcare waterproof tape or a Band-Aid may be used to protect the

wound.

You may continue with vitamin E oil applied one daily. In this case the vitamin E oil can be applied directly to the ulcer.

Debridement should not be performed during the double-blind phase of the study unless it is clinically indicated. (e.g., for treatment of an infection.)

12. References

1. van den Hoogen F, Khanna D, Fransen J, Johnson SR, Baron M, Tyndall A, et al. 2013 classification criteria for systemic sclerosis: an American College of Rheumatology/European League against Rheumatism collaborative initiative. Arthritis Rheum. 2013 Nov;65(11):2737-47.
2. Tingey T, Shu J, Smuczek J, Pope J. Meta-analysis of healing and prevention of digital ulcers in systemic sclerosis. Arthritis Care Res (Hoboken). 2013 Sep;65(9):1460-71.
3. Beyer C, Reich N, Schindler SC, Akhmetshina A, Dees C, Tomcik M, et al. Stimulation of soluble guanylate cyclase reduces experimental dermal fibrosis. Ann Rheum Dis. 2012 Jun;71(6):1019-26.
4. Beyer C, Zenzmaier C, Palumbo-Zerr K, Mancuso R, Distler A, Dees C, et al. Stimulation of the soluble guanylate cyclase (sGC) inhibits fibrosis by blocking non- canonical TGFbeta signalling. Ann Rheum Dis. 2014 Feb 23.
5. Geschka S, Kretschmer A, Sharkovska Y, Evgenov OV, Lawrenz B, Hucke A, et al. Soluble guanylate cyclase stimulation prevents fibrotic tissue remodeling and improves survival in salt-sensitive Dahl rats. PLoS One. 2011;6(7):e21853.
6. Sharkovska Y, Kalk P, Lawrenz B, Godes M, Hoffmann LS, Wellkisch K, et al. Nitric oxide-independent stimulation of soluble guanylate cyclase reduces organ damage in experimental low-renin and high-renin models. J Hypertens. 2010 Aug;28(8):1666-75.
7. Gladue H, Maranian P, Paulus H, Khanna D. Evaluation of test characteristics for outcome measures used in Raynaud's phenomenon clinical trials. Arthritis Care Res (Hoboken). 2013 Apr;65(4):630-6.
8. Merkel PA, Herlyn K, Martin RW, Anderson JJ, Mayes MD, Bell P, et al. Measuring disease activity and functional status in patients with scleroderma and Raynaud's phenomenon. Arthritis Rheum. 2002;46(9):2410-20.
9. Koch GG, Carr GJ, Amara IA, Stokes ME, Uryniak TJ. Categorical data analysis. In: Berry DA, ed. *Statistical Methodology in the Pharmaceutical Sciences*. New York, NY: Marcel Dekker; 1990:389–473.
10. LaVange LM, Durham TA, Koch GG. Randomization-based nonparametric methods for the analysis of multicentre trials. Stat Methods Med Res. 2005;14:281– 301
