# Supplementary material for: A multicenter randomized, double-blind, placebo-controlled pilot study to assess the efficacy and safety of riociguat in systemic sclerosis-associated digital ulcers
Source: Arthritis Res Ther. 2019 Sep 3;21:202. doi: 10.1186/s13075-019-1979-7 (PMC6724329; doi:10.1186/s13075-019-1979-7)
Supplement: Supplementary file 2 — Further details on the statistical analysis. (PDF 681 kb) [file 13075_2019_1979_MOESM2_ESM.pdf]

RESCUE Statistical Analysis Plan

TRIAL FULL TITLE	A MULTI-CENTER RANDOMIZED, DOUBLE-BLIND, PLACEBO-CONTROLLED PILOT STUDY TO ASSESS THE EFFICACY AND SAFETY OF RIOCIGUAT IN SCLERODERMA-ASSOCIATED DIGITAL ULCERS
SAP VERSION	FINAL
SAP VERSION DATE	22AUG2018
TRIAL STATISTICIAN	Cathie Spino, DSc
Protocol Version (SAP associated with)	Protocol Version 4<br>July 10, 2017
TRIAL PRINCIPAL INVESTIGATOR	Dinesh Khanna, MD, MS
SAP AUTHOR(s)	Cathie Spino, DSc

1 SAP Signatures

I give my approval for this SAP entitled "ASSET Statistical Analysis Plan," dated 14SEP2018.

Statistician (Author)
Name: Cathie Spino, ScD

Signature: 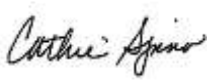
Date: 14AUG2018

Principal Investigator
Name: Dinesh Khanna, MD, MS

Signature: 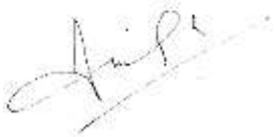

Date: 22AUG2018

2 Table of Contents

1 SAP Signatures 1
2 Table of Contents 2
3 Introduction 4
3.1 Preface 4
3.2 Scope of the analyses 4
4 Study Objectives and End points 4
4.1 Study Objectives 4
4.2 End points 5
5 Study Methods 8
5.1 General Study Design and Plan 8
5.2 Inclusion-Exclusion Criteria and General Study Population 8
5.2.1 Inclusion Criteria 8
5.2.2 Exclusion Criteria 9
5.3 Randomization and Blinding 10
5.4 Study Assessments 10
5.5 Imputation of Dates 13
6 Sample Size 14
7 General Analysis Considerations 15
7.1 Timing of Analyses 15
7.2 Analysis Populations 15
7.2.1 Modified Intention to Treat Population 15
7.2.2 Per Protocol Population 15
7.2.3 Safety Population 15
7.3 Covariates and Subgroups 15
7.3.1 Multi-center Studies 15
7.4 Missing Data 16
7.5 Interim Analyses and Data Monitoring 16
7.6 Multiple Testing 16
8 Summary of Study Data 16
8.1 Subject Disposition 16
8.2 Protocol Deviations 17
8.3 Demographic and Baseline Variables 17
8.4 Treatment Compliance 17
9 Efficacy Analyses 17
9.1 Primary Efficacy Analysis 17
9.2 Secondary Efficacy Analyses 18
9.2.1 Secondary Analyses of Primary Efficacy End point 18
9.2.2 Analyses of Secondary End points 18

10 Safety Analyses 18
10.1 Adverse Events 18
10.2 Deaths, Serious Adverse Events and other Significant Adverse Events 19
10.3 Pregnancies 19
10.4 Clinical Laboratory Evaluations 19
10.5 Extent of Exposure 19
10.6 Prior and Concurrent Medications 20
10.7 Other Safety Measures 20
11 Reporting Conventions 20
12 Summary of Changes to the Protocol and/or SAP 20
15 References 21
16 Listing of Tables, Listings and Figures 21

3 Introduction

3.1 Preface

Systemic sclerosis (SSc) is a rare, orphan disease featuring chronic, fibrosing, autoimmune responses characterized by small vessel vasculopathy, autoantibody production, and fibroblast dysfunction leading to increased deposition of extracellular matrix.

Systemic sclerosis is further divided into 2 subtypes defined by the extent of skin involvement: limited cutaneous systemic sclerosis (lcSSc) and diffuse cutaneous systemic sclerosis (dcSSc). dcSSc is one of the most fatal rheumatic diseases, and is associated with substantial morbidity and many detrimental effects on health-related quality of life.

Raynaud's phenomenon (RP) is an almost universal manifestation of SSc, with 95% of all patients being affected, and resulting in digital ulcers (DUs) in approximately 30% of the patients each year. DUs are a major clinical problem, being associated with substantial morbidity (reduced quality of life, pain, disability and disfigurement) that can escalate to gangrene and amputation in approximately 15% of patients. Treatments that have shown potential include calcium channel blockers, prostacyclin analogues and endothelin receptor antagonists (ERAs).

Riociguat is the first-in-class of a new group of compounds, soluble guanylate cyclase (sGC) stimulators. Riociguat directly stimulates sGC, thereby increasing levels of the signaling molecule cGMP. The cGMP molecule plays a pivotal role in regulating cellular processes, such as vascular tone, proliferation, fibrosis, and inflammation. Two key features of riociguat are (i) it directly stimulates sGC independently of nitric oxide (NO), and (ii) it sensitizes sGC to low levels of NO. Riociguat has recently been approved in the US and Canada for two forms of pulmonary hypertension, namely pulmonary arterial hypertension (PAH) and treatment of chronic thromboembolic pulmonary hypertension (CTEPH).

The goal of the current study is to provide preliminary data on the efficacy and safety of 16 weeks of treatment with riociguat in a randomized, placebo-controlled clinical trial in patients with SSc-associated DU.

3.2 Scope of the analyses

These analyses will assess the efficacy and safety of riociguat in comparison with placebo during the 16-week double-blind period, addressing the primary and secondary objectives of study through the double-blind period. The objectives associated with the open-label extension period are not included in this SAP.

4 Study Objectives and End points

4.1 Study Objectives

Primary Objectives

- To provide preliminary data on the efficacy (digital ulcer net burden) of riociguat administered 3 times daily (TID) in comparison to placebo in patients with SSc.

Secondary Objectives

- To provide preliminary data on safety and additional measures of efficacy of riociguat administered TID as compared with placebo.

4.2 End points

4.2.1 Primary Efficacy End point

Change from baseline to end of double-blind treatment (week 16) in digital ulcer net burden. Digital ulcer net burden is defined as the total number of "active" and indeterminate digital ulcers at an assessment.

4.2.2 Secondary Efficacy End points

- Proportion of participants with healing of their cardinal DU by week 16. For each participant, one digital ulcer must be identified and designated by the investigator as the cardinal ulcer at screening. The cardinal ulcer must have met the qualifications for designation as an active or painful indeterminate ulcer. If only one ulcer was determined at entry to be active or painful indeterminate, it will be designated as the cardinal ulcer. If several digital ulcers qualified, the cardinal ulcer could be either the largest or the most painful ulcer, or the ulcer that disturbed the patient the most. The cardinal ulcer will be selected by the investigator based on the clinical judgment that it was amenable to and evaluable for healing. Cardinal ulcers are considered healed when classified as 'healed' and not 'active' or 'indeterminate' by week 16.
- Proportion of participants with healing of all DUs at baseline by week 16. Baseline DUs are considered healed when classified as 'healed' and not 'active' or 'indeterminate' by week 16. **All** baseline ulcers must be healed for the participant to be classified as having all baseline ulcers healed. Note that this end point does not consider whether a participant develops new DUs during the course of the study.
- Proportion of participants with no DUs at week 16. This end point does not consider the number of ulcers at baseline or during the course of the study; only the absence of 'active' and 'indeterminate' DUs at week 16.
- Proportion of participants with and number of new active and indeterminate DU(s) over the course of the double-blind period.
- Proportion of participants who develop pressure ulcers by location -- Distal Interphalangeal (DIP), Proximal Interphalangeal (PIP), Metacarpophalangeal (MCPs) and elbows – over the course of the double-blind period.
- Proportion of participants with healing of all pressure ulcers by location -- Distal Interphalangeal (DIP), Proximal Interphalangeal (PIP), Metacarpophalangeal (MCPs) and elbows –by the course of the double-blind period. Note that this is defined among those who had pressure ulcer(s) at a certain location at baseline or who had one develop during the course of the double-blind treatment period.
- Time to healing of cardinal DU, defined as the number of weeks from randomization to the earliest of healing, end of the double-blind period, or drop-out. Participants are censored if they drop-out or their cardinal DU has not healed by the end of the double-blind period.
- Time to healing of all baseline DU, defined as the number of weeks from randomization to the earliest of all baseline DU(s) healed, end of the double-blind period, or drop-out. Participants are censored if they drop-out or all of their baseline DU(s) have not healed by the end of the double-blind period.
- Time to development of new ('active' or 'indeterminate') DU during the double-blind period of the trial, defined as the number of weeks from randomization to the earliest of new DU, end of the double-blind period, or drop-out. Participants are censored if they drop-out or have not developed a new DU by the end of the double-blind period.
- Improvement of Raynaud's phenomenon (RP). A diary is completed by each participant to collect daily RP information. Each characteristics is reported by the participant daily for at least 7 consecutive days during the screening phase and at least 7 consecutive days in the 2 weeks between the week 12 and week 16 visits. If less than 7 days are marked, then the average is calculated by accounting by the days where diary is completed (included the days where participants have no RP attack).
    - Raynaud's condition score. The score ranges from 0 to 10 and indicates the difficulty the participant had with their Raynaud's condition. The mean score will be

calculated across the 7-day screening and week 16 periods for each participant.
  - Number of Raynaud's attacks/day. The mean number of Raynaud's attacks each day will be calculated across the 7-day screening and week 16 periods for each participant. For the days when a participant does not have an attack, a score of 0 will be used.
  - Duration of attacks. For the days when a participant does not have an attack, a score of 0 will be used. The mean duration of attacks (in minutes) will be calculated across the 7-day screening and week 16 periods for each participant.
  - Patient assessment of RP: pain, numbness, and tingling during an RP attack. Patient assessments of RP pain, numbness and tingling symptoms are reported on a 0-100 scale. For the days when a participant does not have an attack, a score of 0 will be used. The mean scale score for each symptom will be calculated across the 7-day screening and week 16 periods for each participant.
- Physician assessment of RP:
  - Severity of patient's Raynaud's disease. The mean response on a 0-10 Likert scale will be calculated.
  - Severity of patient's digital ulcer(s). The mean response on a 0-10 Likert scale will be calculated.
  - Change in patient's digital ulcers at Week 16. Physician's responses to the question "At Week 16, compared to the start of the trial do you consider your patient's digital ulcer(s)" as much better | a little better | no change | a little worse | much worse will be summarized as a categorical variable.
  - Change in patient's Raynaud's disease at Week 16. Physician's responses to the question "At Week 16, compared to the start of the trial do you consider your patient's Raynaud's disease" as much better | a little better | no change | a little worse | much worse will be summarized as a categorical variable.
- Patient assessment of RP:
  - Severity of Raynaud's disease. The mean response on a 0-10 Likert scale will be calculated.
  - Severity of digital ulcer(s). The mean response on a 0-10 Likert scale will be calculated.
  - Change in digital ulcers at Week 16. Patient's responses to the question "Compared to START OF THE TRIAL, do you consider that your digital ulcer(s) is:" as much better | a little better | no change | a little worse | much worse will be summarized as a categorical variable.
  - Change in Raynaud's disease at Week 16. Patient's responses to the question "Compared to START OF THE TRIAL, do you consider that your Raynaud's disease is:" as much better | a little better | no change | a little worse | much worse will be summarized as a categorical variable.
- Change from baseline to each follow-up week in:
  - Patient's global assessment for overall disease. Patient global assessment for overall disease: This assessment represents the patient's assessment of the patient's global scleroderma on a 0-10 Likert scale. "On a scale of 0-10, how was your overall health in the last week? 0=Excellent; 10=Extremely Poor.
  - Physician's global assessment for overall disease. Physician global assessment for overall disease: This assessment represents the physician's assessment of the patient's current disease activity on a 0-10 Likert scale. "On a scale of 0-10, how was your patient's overall health in the last week? 0=Excellent; 10=Extremely Poor".
  - PROMIS-29 Profile v2.0 measures in the following domains: physical function, anxiety, depression, fatigue, sleep disturbance, pain interference, and ability to participate in social roles and activities, and a single item on pain intensity. PROMIS-29 Profile v2.0 measure: The National Institutes of Health (NIH) Patient-Reported Outcomes Measurement Information System (PROMIS®) Roadmap initiative (www.nihpromise.org) is a cooperative research program designed to

develop, evaluate, and standardize item banks to measure patient-reported outcomes (PROs) across different medical conditions as well as the US population. PROMIS-29 Profile v2.0 measure contains 29 items, which includes four items each from physical function, anxiety, depression, fatigue, sleep disturbance, pain interference, and satisfaction with social roles domains, and a single item on pain intensity. With the exception of physical function which does not include a time frame, all item banks reference the past 7 days. Three scores are available for each PROMIS domain: cumulative score, instrument score and transformed score; the transformed score (Tscore) will be the score analysed in this study.

  - HAQ-DI overall (HAQ-DI) and 8 categories: Dressing and Grooming, Hygiene, Arising, Reach, Eating, Grip, Walking, Common Daily Activities (IADL). The SHAQ consists of the HAQ-DI (8 domains and an overall score) and 6 visual analogue scales assessing the burden of pain, digital ulcers, Raynaud's, gastrointestinal involvement, breathing, and overall disease. The HAQ-DI is a disease-targeted, musculoskeletal-targeted measure intended for assessing functional ability in arthritis. It is a self-administered 20-question instrument that assesses a patient's level of functional ability and includes questions that involve both upper and lower extremities. The score for each question ranges from 0 (no disability) to 3 (severe disability). There are 8 categories and an overall score (HAQ-DI). It has a 7-day recall period and has been extensively used in SSc.
  - Composite score for hand function. The sum of the individual scores for dressing, hygiene, and grip from the HAQ-DI will be calculated. These categories are associated with hand function.
  - The Hand Disability in Systemic Sclerosis-DU (HDISS-DU). Participants were asked to answer 24 questions on the use of the hand(s) affected by DUs over the past 7 days on a 6-point scale from scored from 1–6 (1=yes, without difficulty; 2=yes, with a little difficulty; 3=yes, with some difficulty; 4=yes with much difficulty; 5=nearly impossible to do & used unaffected hand only; 6=impossible). Note that "used unaffected hand only" should be rescored to 5. The response option of "Did not do this activity in the past 7 days" will be scored as missing. The total HDISS-DU® score is calculated as a mean of valid items, with a total number of possible valid items equal to 24; higher scores represent increased disability in hand functioning. For missing items/values, the average score will be calculated out of the number of valid items; for example, if three items are missing from the questionnaire, the average will be calculated as the sum of the 21 valid items divided by 21.
  - Scleroderma-HAQ-DI visual analogue scales (VAS) assessing burden of digital ulcers, Raynaud's disease, gastrointestinal involvement, breathing, and overall disease.
- Proportion of participants who experience digital ischemia requiring intravenous prostacyclin or digital gangrene or amputation during the trial.
- Proportion of participants who develop osteomyelitis during the trial
- Changes in vascular biomarkers in the plasma (VEGF, tPA, sE-Selectin, BFGF, VCAM-1, ICAM) from baseline to week 16. Change is defined as (1) the absolute difference (week 16 – baseline value) and as (2) the proportion of participants whose values are > 2 standard deviations from the mean, based on a population of healthy volunteers who are matched on sex and age to those from the RESCUE study.

4.2.3 Secondary Safety End points

- Adverse events
- Adverse events of special interest: symptomatic hypotension; serious haemoptysis
- Clinically significant changes in vital signs
- Laboratory test abnormalities. In general, for quantitative laboratory values reported as "<" or "≤" the lower limit of quantitation (LLOQ) or limit of detection (LOD), one-half of the reported value (i.e., LLOQ/LOD) will be used for analysis. For quantitative laboratory values

reported as “>” or “≥” the upper limit of quantitation (ULOQ), the reported value (i.e., ULOQ) will be used for analysis. The number and percentage of subjects with values < or > limits of quantitation or detection will also be provided.

For analysis purposes, repeat laboratory test results will not be used unless the original laboratory value is missing or indicated as invalid, in which case the first non-missing repeat laboratory value will be used for data analysis.

- Clinical tolerability of the drug

5 Study Methods

5.1 General Study Design and Plan

This clinical trial is a US, multicenter, double-blind, randomized placebo-controlled, parallel- group study with a total of 20 participants planned to be randomized (approximately 10 participants to the riociguat group and 10 to the placebo group). In addition, a standardized wound care protocol (see Appendix 11.2) will be followed by the investigators and digital photography will be taken of the cardinal ulcer.

The study will allow standard of care medications for the management of DU as background therapy. These may include calcium channel blockers, low dose aspirin, angiotensin enzyme inhibitors, etc. and will be determined by the participant’s local physician.

The schema below describes the main elements of the study design:

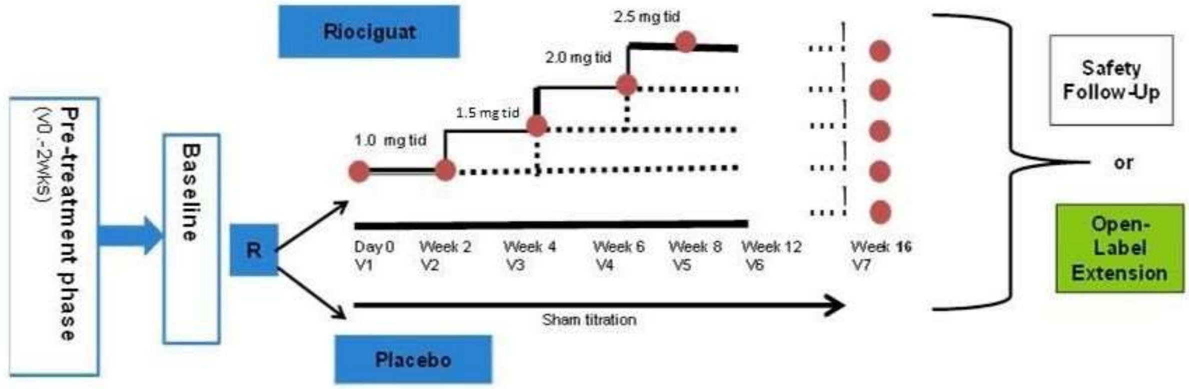

Figure 4-1. Main Study Treatment phase design
Abbreviations: V = visit; R = randomization; TID = 3 times a day.

5.2 Inclusion-Exclusion Criteria and General Study Population

5.2.1 Inclusion Criteria

1. Signed written informed consent
2. Men or women aged 18 years and older
3. Diagnosis of Systemic sclerosis, as defined by 2013 American College of Rheumatology/ European Union League Against Rheumatism classification of SSc
4. Patients had to have at least one visible, active ischemic DU or painful indeterminate DU at screening located at or distal to the proximal interphalangeal joint, and that developed or worsened within 8 weeks prior to screening.
5. Females of reproductive potential (FRP) must have a negative, pre-treatment urine pregnancy test.

6. FRP must obtain monthly urine pregnancy tests during treatment and one month after treatment discontinuation. Post-menopausal women (defined as no menses for at least 1 year or post-surgical from bilateral oophorectomy) are not required to undergo a pregnancy test.
7. FRP and all non-vasectomized male participants must agree to use reliable contraception when sexually active. (For FRP's, 'Adequate contraception' is defined as any combination of at least 2 effective methods of birth control, of which at least one is a physical barrier (e.g., condoms with hormonal contraception or implants or combined oral contraceptives, certain intrauterine devices). This applies from the time of signing the informed consent form until one month after the last study drug administration.)
8. Oral corticosteroids (≤ 10 mg/day of prednisone or equivalent), nonsteroidal anti-inflammatory drugs (NSAIDs), angiotentin receptor blockers, angiotensin converting enzyme (ACE) inhibitors and calcium channel blockers are permitted if the participant is on a stable dose for ≥ 2 weeks prior to and including the baseline visit
9. Ability to comply with the clinical visits schedule and the study-related procedures.

5.2.2 Exclusion Criteria

1. Active DU related to calcinosis (as assessed by clinical examination or radiographic evaluation at screening)
2. Medical and surgical history
   - Major surgery (including joint surgery) within 8 weeks prior to screening
   - Participants with a history of malignancy in the last 5 years other than non-melanoma skin cell cancers cured by local resection or carcinoma in situ
3. Hepatic-related criteria
   - Hepatic insufficiency classified as Child-Pugh C at screening (see Appendix 11.1 for classification table) at screening visit
4. Renal-related criteria
   - Estimated glomerular filtration rate (eGFR) < 15 mL/min/1.73m2 (MDRD formula) or on dialysis at the screening visit
5. Cardiovascular-related criteria
   - Sitting systolic blood pressure < 95 mmHg at the screening visit
   - Sitting heart rate < 50 beats per minute (BPM) at the screening visit
   - Left ventricular ejection fraction < 40% prior to screening on echocardiogram done as part of clinical care
6. Pulmonary-related criteria
   - Active state of hemoptysis or pulmonary hemorrhage, including those events managed by bronchial artery embolization
   - Any history of bronchial artery embolization or massive hemoptysis within 3 months prior to screening. Massive hemoptysis being defined as acute bleeding >240 mL in a 24-hour period or recurrent bleeding >100 mL/d over several days
   - PAH requiring pharmacologic therapy.
   - Significant pulmonary disease with FVC ≤ 50% of predicted, or DLCO (uncorrected for hemoglobin) ≤ 40% of predicted
7. Laboratory examinations
   - Participants with hemoglobin < 9.0 g/dL, white blood cell (WBC) count < 3000/mm^3 (< 3 × 10^9/L), platelet count < 100,000/mm^3 (< 3 × 10^9/L) at the screening visit
8. Prior and concomitant therapy
   - Concomitant use of nitrates or NO donors (such as amyl nitrate) in any form, including topical; phosphodiesterase (PDE) 5 (PDE5) inhibitors (such as sildenafil, tadalafil, vardenafil); and nonspecific PDE5 inhibitors (theophylline, dipyridamole). If the patient is on PDE5 inhibitors, a wash out of 3 days is required for sildenafil and 7 days for tadalafil or vardenafil prior to the baseline visit
   - Concomitant Endothelin receptor antagonist

- Patients who are actively smoking at time of consent. (Quit date of two weeks prior to screening acceptable)

9. Pregnant or breastfeeding women
10. Other
    - Any other condition or therapy that would make the participant unsuitable for this study and will not allow participation for the full planned study period
    - Participation in another clinical study with an investigational drug or medical device within 30 days prior to randomization (phase I-III clinical studies)

Note: One re-assessment of laboratory parameters is allowed during the screening phase to assess the eligibility of participants.

5.3 Randomization and Blinding

At the baseline visit, participants who met all of the inclusion and none of the exclusion criteria were randomized in 1:1 fashion to riociguat or placebo using a web-based randomization system. At the time of randomization, patients were assigned a unique randomization number; no participant was to begin treatment prior to randomization. The DCC prepared the randomization schedule, using computer-generated block randomization with the block size(s) known only by the DCC. A secure web-based application was built for use by the coordinators to enter participant information (e.g., participant ID, stratification factor(s)) and to obtain the randomization number. The coordinators printed the randomization # and bottle assignment information from this system and sent the information to the research pharmacy for dispensing. The study was conducted in double-blind fashion. Active riociguat and placebo tablet formulations were identical in appearance (size, shape, color) and smell. The packaging and labeling was designed to maintain blinded conditions for the investigator's team and the participants. The study data remained blinded until database lock and authorization of data release according to standard operating procedures.

5.4 Study Assessments

The following table provides the Schedule of Evaluations used in the study:

**Study Period: Double-Blind Phase**		**Dose Titration Period**					**Stable Dosing Period**		**Safety[h] Follow Up**
**Study Visit**	**Screening** Visit 0	**Baseline** Visit 1	**Week 2** Visit 2	**Week 4** Visit 3	**Week 6** Visit 4	**Week 8** Visit 5	**UNSH[f]**	**Week 12** Visit 6	**Week 16[g]** Visit 7	**Week 20** Visit 8
**Window (days):**	14	+4	+/-4	+/-4	+/-4	+/-4		+/-4	+/-4	+/-4
**Type of Contact:**	**Office**	**Office**	**Office**	**Office**	**Office**	**Office**	**Office**	**Office**	**Office**	**Office/Phone Call**
Informed consent	X									
Eligibility assessment	X	X								
Demographics, including smoking & alcohol history	X									
Complete medical history	X									
Prior/Concomitant therapy	X	X	X	X	X	X		X	X	X
Vitals	X	X	X	X	X	X	X	X	X	X
Physical examination	X			X			X		X	X
12-lead ECG	X					X			X	
Hematology, serum chemistry[a]	X					X			X	
Urine pregnancy test (women of childbearing potential)	X	X		X		X		X	X	
Hand x-rays for calcinosis (*not required at Screening if performed in prior 6 months*)	X									
Physician's Global Assessment		X							X	
Patient's Global Assessment		X							X	
PROMIS -29		X							X	
HAQ-DI/SHAQ		X							X	
HDISS-DU		X							X	
Raynaud's phenomenon diary	X[b]	X[c]						X[b]	X[c]	
Digital Ulcer Assessment	X	X	X	X	X	X		X	X	X
Digital photo of cardinal ulcer	X								X	
Dispense study medication		X	X	X	X	X	X	X	X[d]	
Assess for Adverse Events		X	X	X	X	X	X	X	X	X
Plasma Biomarkers[e]		X							X	

a	Participants with eGFR 15-29 ml/min or other laboratory abnormalities per physician judgment can repeat labs once during the screening period
b	Dispense 7-day diary;
c	Collect 7-day diary;
d	Open-Label study drug will be dispensed at this visit if participant is continuing to the OLE phase of the study. If a participant is exiting the study, all study drug should be returned at this visit and no more will be dispensed.
e	10 mL for biomarkers: VEGF, tPA, sE-Selectin, BFGF, VCAM-1, ICAM
f	This visit is for participants who experience toleration issues and return to clinic for assessment and dispense a lower dose.
g	Complete these visit assessment for any participants who terminate/withdrawal from the study prior to visit 7.
h	Complete this visit for any subject who does not enter the OLE. This can be a phone call if there are no new or ongoing adverse events attributable to the study medication and no digital ulcers to assess.

- Given flexibility around subject and care provider scheduling, visits are not required to have occurred on a specific date, but rather within a defined window.
- Where analyses reference the timing of outcomes and/or covariates, the nominal visit or time point as collected in the database will be used. For instances when there are scheduled visits and unscheduled visits in the same analysis time window, scheduled visits will be selected over unscheduled visits. If there are multiple observations for scheduled visits within a window, the one closest to the visit target date will be utilized. Where two observations are equi-distant from the target date the later will be utilized.

Analysis Time Windows

Period	Visit<br>Study Month (Day)	Lower bound of Window (Day)	Target Date (Day) and Per protocol window	Upper bound of Window (Day)
Screening	Screening	-14	-14 to -1	-1
Dose Titration	Baseline (0)	0	0 to 4	7
	Week 2 (14)	8	14 ± 4	21
	Week 4 (28)	22	28 ± 4	35
	Week 6 (42)	36	42 ± 4	49
	Week 8 (56)	50	56 ± 4	70
Stable Dosing	Week 12 (84)	71	84 ± 4	98
	Week 16 (112)	99	112 ± 4	126
Safety Follow-up	Week 20 (140)*	127	448 ± 10	154

*participants who enter into the open-label phase of the study did not have a week 20 visit; participant data from the open-label extension period are not included in the study.

5.5 Imputation of Dates

If partial dates occur, the convention for replacing missing dates for the purpose of calculating derived variables is as follows:

For partial original baseline or historical condition dates (e.g., diagnosis): (a) if only the day is missing, and the month and year match the first dose date, then the day is assigned the first day of the month (01); otherwise the day assigned is 15; and (b) if both the day and month are missing then the day/month assigned is the first day of July (01JUL), as long as the date is before the first dose date; otherwise, the day/month assigned is the first day of January (01JAN).

If start dates are entirely missing for adverse events or medications, then adverse events will be classified as treatment-emergent and medications will be classified as concomitant. For partial AE or concomitant medication start dates or end dates, the table below describes the date imputation.

Missing	Condition – START DATE	Condition – END DATE	Imputation	Classification
Start Day	Start month & year match those of the first dose date	End date ≥ first dose date or ongoing	Set start date = first dose date	AE = treatment-emergent & med = concomitant
		End date < first dose date	Set start date = 01	AE = not treatment emergent & med = prior
	Start year < first dose year	End date ≥ first dose date or ongoing	Set start date = 15	AE = not treatment-emergent & med = concomitant
		End date < first dose date	Set start date = 01	AE = not treatment emergent & med = prior
	Start year = first dose year and start month < first dose month	End date ≥ first dose date or ongoing	Set start date = 15	AE = not treatment-emergent & med = concomitant
		End date < first dose date	Set start date = 01	AE = not treatment emergent & med = prior
	Start year = first dose year and start month > first dose month	All cases	Set start date = 15	AE = treatment-emergent & med = concomitant
Start Day & Start Month	Start year matches first dose date	End date ≥ first dose date or ongoing	Set start date = first dose date	AE = treatment-emergent & med = concomitant
		End date < first dose date	Set start date = 1 and start month = end date month	AE = not treatment emergent & med = prior
	Start year < first dose year	All cases	Set start date = 1 and start month = JAN	
	Start year > first dose year	All cases	Set start day =1 and start month = JAN	AE = treatment-emergent & med = concomitant
End Day	NA	All	Set end date = min(last day of month, day of visit date)	NA
End Day & End Month	NA	All	Set end day =31 and end month = DEC, or day and month of visit date if earlier	NA

6 Sample Size

SSc is a rare disease. The planned sample size of 20 SSc participants is based on practical considerations to obtain preliminary estimates of the magnitude of treatment differences in efficacy and safety rather than a desired power for a pre-specified difference as would be necessary for a

confirmatory study. However, with this proposed sample of 20 participants (10 riociguat and 10 placebo), we can calculate the magnitude of treatment differences (riociguat – placebo) for the primary efficacy endpoint – the change from baseline to end of double-blind treatment in digital ulcer net burden (a continuous endpoint), or safety outcomes – characterized by the proportion of participants who experience an AE. There would be 80% power to detect an effect size (mean treatment difference divided by standard deviation) of 1.253 or greater with a two-sided type I error of 5% in the primary endpoint, based on a two- sample t test. Given the pilot nature of this Phase IIa study, the difference between mean change in digital ulcer net burden between riociguat and placebo that can be detected with sufficient power is large. Similarly, we can calculate power for safety outcomes for this sample size: there is 81% power to detect treatment differences of 51%, assuming 40% of placebo-treated participants experience an AE based on a two-sample binomial test.

7 General Analysis Considerations

7.1 Timing of Analyses

The final analysis of the double-blind period will be performed after all randomized participants have completed their 16-week visit or dropped out prior to their 16-week visit, all corresponding data have been entered, cleaned, locked and unblinded as per SABER SOPs. This SAP document was finalized and approved prior to the double-blind database lock and unblinding.

7.2 Analysis Populations

All randomized participants will be used in the analyses of subject disposition.

7.2.1 Modified Intention to Treat Population

The main population for efficacy will be the modified intention-to-treat population (MITT), defined as all participants randomized, receiving at least one dose of treatment, and having at least one post-baseline efficacy assessment. Participants will be analyzed by assigned treatment. The primary endpoint and all secondary outcomes will be assessed using this analysis set. Membership in the mITT analysis population was determined before study unblinding.

7.2.2 Per Protocol Population

The Per Protocol population (PP) will consist of all participants in the MITT population who did not have a major protocol violation. Major protocol deviations are defined as eligibility criteria violations for which no exemption was granted, and study drug compliance <80% and >120%. Membership in the PP analysis population will be determined before study unblinding. Only the primary endpoint will be assessed using this analysis set (sensitivity analysis).

7.2.3 Safety Population

The Safety Population is defined as all participants who were randomized and received at least one dose of the study drug. The Safety Population will be used for all safety analyses, as well as demographic and baseline analyses. Participants will be analyzed by treatment received. If participants inadvertently receive both active drug and placebo, they will be included in the riociguat group.

7.3 Covariates and Subgroups

There are a limited number of covariates that will be incorporated in statistical models in our analyses because of the relatively small sample size in each treatment group: the baseline outcome measure. We will not impute missing values for other baseline covariates in secondary and exploratory analyses in the mITT analysis set.

No planned subgroup analysis will be performed using the primary end point.

7.3.1 Multi-center Studies

Given that SSc is a relatively rare disease, many centers were required to obtain the required sample

size. Study centers will not be incorporated as stratification into the analyses. Descriptive statistics of the primary end point by treatment group, separately by center (Michigan [the largest enrolling site] and the remainder) will be provided.

7.4 Missing Data

We will summarize the extent of missing data over time for the primary end point. We will investigate the missing data mechanism (missing at random, not missing at random), which is important for the validity of our analytic approaches, through exploratory analysis. Exploratory analyses will include plots of the mean profile of digital ulcer net burden at weeks 0, 2, 4, 6, 8, 12 and 16 by treatment group for those who have complete data throughout the study and those who don't, as well as plots of the mean change from baseline at weeks 2, 4, 6, 8, 12 and 16 in digital ulcer net burden in the two treatments within each group (completers and non-completers). If the plots reveal consistent differences between completers and non-completers within each of the treatment groups, then there is evidence that data are not missing at random.

The primary analysis of the primary end point (see section 9.1) will use multiple imputation (which assumes a missing at random mechanism) to impute missing week 16 digital net ulcer burden values. The imputation model will include baseline digital ulcer net burden and all follow-up digital ulcer net burden values (allowing for the dependence of later time points on earlier time points), treatment group, and demographic variables (age and gender). The analysis model will incorporate the uncertainty due to imputation in the calculation of the standard error, as described by Rubin.[1] Pattern mixture models will be used in there is strong evidence that the data are not missing at random.[2]

7.5 Interim Analyses and Data Monitoring

No formal interim analyses were planned nor carried out for this study. The study was overseen by a Data and Safety Monitoring Board (DSMB) that reviewed the pooled and by-treatment subject disposition, study conduct and safety data approximately every 6 months.

7.6 Multiple Testing

Two-sided p-values will be reported, and no adjustments for multiplicity will be made. Given the rare nature of SSc and the consequent small sample size for this pilot Phase IIa study, the statistical power of any comparisons is limited (i.e., there is sufficient power to detect only large treatment differences). As such the analysis will be largely descriptive in nature as the study is not powered to determine a statistical difference between riociguat vs. placebo. The p-values resulting from formal statistical tests will be interpreted from a hypothesis-generating, rather than a confirmatory framework.

8 Summary of Study Data

Descriptive summary statistics will be derived for all data at baseline, separately by treatment group and overall. For efficacy, exploratory and safety data, data will be presented by treatment group. Treatment group will be characterized as "Riociguat" and "Placebo"; for pooled summaries, "Overall" will be used as the column heading. All tables will be annotated with the total population size relevant to that table/treatment, including any missing observations.

For continuous variables, mean, standard deviation, median, interquartile range, minimum and maximum will be reported. For categorical variables, number and percentages will be reported (excluding missing values). Summaries will be provided by treatment group and overall. Graphical methods will be heavily used in this pilot study to assess the pattern of response over time for key variables and to assess the relationships among variables.

8.1 Subject Disposition

The number of participants approached for study participation, the number consented and the number who did not consent (including reasons: screen failures, refusals) will be summarized in a CONSORT

diagram. The number of participants who dropped out prior to randomization, and the reasons for dropout, will be summarized. The number randomized and treated and the number who dropped out in the dose titration period (baseline – week 8) and in the stable dosing period (week 8 – week 16) will be provided, as well as the number in each of the analysis populations (i.e., mITT, PP, Safety). Reasons for post-treatment dropout will be provided.

8.2 Protocol Deviations

Major protocol deviations that exclude a patient from the Per Protocol Population are described in section 7.2.2. A listing of protocol deviations that exclude participants from the Per Protocol population will be provided. A listing of participants who receive exemptions for study eligibility will also be provided.

8.3 Demographic and Baseline Variables

Demographic variables include: age at consent (defined as a continuous variable, e.g., 52.6 years), age by category (18 to 35 years, >35 to 55 years, >55 to 75 years, and >75 years), gender, race and ethnicity.

Baseline is defined as pre-treatment measures, either at screening (if a measure was only assessed at screening) or at baseline (if a measure was assessed at baseline even if also assessed at screening). Baseline variables include:

- Number of active, indeterminate and total digital ulcers at baseline
- Disease duration since first non-Raynaud's symptom at time of consent (years)
- Disease duration since Raynaud's phenomenon at time of consent (years)
- Disease duration of scleroderma diagnosis at time of consent (years)
- Duration of digital ulcers at time of consent (years)
- SHAQ VAS finger ulcers interfere with daily activities
- Raynaud's condition score
- Raynaud's attacks/days
- Pain, numbness, and tingling during a RP attack
- Duration of attacks
- Patient assessment of RP
- Physician assessment of RP
- Creatinine (mg/dL), hemoglobin (g/dL), platelets (K/µl), WBC (K/ µl) (all at screening)

8.4 Treatment Compliance

Compliance with study medication (tablets) will be calculated, for each participant, as the proportion of time (weeks) that a participant took the full or partial contents of the syringe. Specifically, the percent compliance is calculated as 100 x the ratio of the number of weeks during the double-blind period when the participant took the tablets divided by the number of weeks during the double-blind period during which the participant was expected to take study medication. Participants were expected to take study medication unless it was temporarily discontinued due to an AE or permanently discontinued. The study medication log (Form 027), adverse event form (Form 044), serious adverse event form (Form 045) and final status form (Form 035) are used to derive the compliance measure.

The summary statistics will be produced in accordance with section 9.

9 Efficacy Analyses

9.1 Primary Efficacy Analysis

The primary endpoint is the mean change from baseline to end of the double-blind study treatment phase (Week 16) in the digital ulcer net burden. For the primary analysis, changes in digital ulcer net burden will be compared in the two treatment groups using an ANCOVA model with terms for treatment group and baseline digital ulcer net burden value. If the assumptions of this parametric

model are not met, an alternative non-parametric model will be used. This model is based on the extension of the Wilcoxon rank-sum test to allow for covariate adjustment. This rank ANCOVA can provide additional power associated with baseline covariate adjustment, even when the outcome variable is not normally distributed.

Predicted mean change from baseline to week 16 for an exemplary participant by treatment group will be provided, as well as the estimate of the treatment effect at week 16, adjusted for baseline covariates, and corresponding 95% confidence interval and p-value for the treatment effect.

9.2 Secondary Efficacy Analyses

9.2.1 Secondary Analyses of Primary Efficacy End point

Several sensitivity analyses will be performed to assess how alternative approaches to missing data and model assumptions affect the conclusions of the analysis of the primary outcome:

- Analysis of the primary efficacy variable as described above will also be performed on the PP Population.

9.2.2 Analyses of Secondary End points

Analysis for secondary outcome measures that are continuous will be performed using a similar approach as that for the primary endpoint. We will compare the change in each secondary outcome measure from baseline to week 16 between the two treatment groups using an ANCOVA model or its non-parametric counterpart if the model assumptions aren’t met. Analyses of secondary outcomes measures that are discrete will be performed using Fisher’s exact tests. Analyses of secondary outcome measure that are counts will be performed using Poisson regression. Analyses of time-to-event secondary outcome measures will be performed using log-rank tests and summarized using Kaplan-Meier survival plots.

10 Safety Analyses

Safety data, including AEs, clinical laboratory tests, vital signs, physical examinations, and concomitant medication usage will be summarized descriptively by treatment group for the Safety Population; select parameters will also be summarized for the entire population (overall).

10.1 Adverse Events

Descriptive summary statistics for treatment-emergent adverse events (AEs) will be reported. The number of treatment-emergent AEs and the frequencies (number and percentage) of participants with one or more treatment-emergent AE will be summarized by treatment group, overall, by severity, and by body system. Coding of adverse events into body system was performed by the study chair for adverse events and by the medical monitors for serious adverse events. All treatment-emergent AEs related to study drug will be summarized, as will the frequencies of participants with one or more treatment-emergent AE related to study drug. Similarly, all treatment-emergent AEs causing study discontinuation, and frequencies of participants experience these, will be summarized.

AEs and SAEs of special interest include:

- Symptomatic hypotension
- Serious hemoptysis

A listing of these AEs will be presented.

A subject listing of all treatment-emergent AEs and treatment-emergent AEs causing study discontinuation will be presented.

In accordance with clincaltrial.gov reporting requirements, the following table summarizing adverse events is required and will be provided:

- Other (Not Including Serious) Adverse Events: A table of anticipated and unanticipated events (not included in the serious adverse event table) that exceed 5% within either treatment group, grouped by organ system, with number and frequency of such events in each treatment group.

Adverse events that occurred after consent and before treatment will be listed.

10.2 Deaths, Serious Adverse Events and other Significant Adverse Events

Descriptive summary statistics for treatment-emergent serious adverse events (SAEs) will be reported. The number of treatment-emergent SAEs and the frequencies (number and percentage) of participants with one or more treatment-emergent SAE will be summarized by treatment group, overall and by body system. Coding into body system was performed by the medical monitors for SAEs. All treatment-emergent SAEs related to study drug will be summarized, as will the frequencies of participants with one or more treatment-emergent SAE related to study drug. Similarly, all treatment-emergent SAEs causing study discontinuation, and frequency of participants experiencing these, will be summarized.

A subject listing of all treatment emergent SAEs, SAEs causing study discontinuation, and deaths (including the post-treatment follow-up period through month 12) will be presented.

In accordance with clincaltrial.gov reporting requirements, the tables below summarizing deaths and SAEs are required:

- All-Cause Mortality: A table of all anticipated and unanticipated deaths due to any cause, with number and frequency of such events in each treatment group.
- Serious Adverse Events: A table of all anticipated and unanticipated serious adverse events, grouped by organ system, with number and frequency of such events in each treatment group.

10.3 Pregnancies

A listing of all pregnancies occurring after the start of study medication will be provided.

10.4 Clinical Laboratory Evaluations

The safety evaluation of laboratory data will include:

- Incidence rates of treatment-emergent laboratory values outside of normal range.
- Descriptive analysis of continuous laboratory parameters, and their changes from baseline by treatment group and visit.

Descriptive analysis of vital signs, and their changes from baseline, will be performed by treatment group and visit.

For ECGs, the status pre-treatment and post-treatment will be tabulated. The incidence rates of treatment-emergent ECG abnormalities will be tabulated by treatment group. A descriptive analysis of continuous ECG parameters and their changes from baseline by treatment group and visit will also be presented.

10.5 Extent of Exposure

The following outcomes will be summarized descriptively by treatment group:

- Duration of treatment: Treatment duration (in days) is calculated as the stop date of treatment – start date of treatment + 1. Treatment interruptions are ignored.
- Extent of exposure: Exposure (in mg) is calculated for each participant by adding the daily dose (TID dosing is prescribed) over the duration of treatment.

- Cumulative treatment exposure: The number of participants treated at least 1 day, 8 weeks (through the dose titration period) and 16 weeks (through the stable dosing period) will be calculated and summarized.
- Study treatment dose titration by visit: The number of participants at each dose for each visit will be summarized.
- Study treatment dose titration: The number of participants for each dosing pattern (across visits in the dose titration and stable dosing periods) by visit will be summarized.

10.6 Prior and Concurrent Medications

The proportion of participants on immunosuppressive therapies, vasodilators, and PDE-5 inhibitors prior to the start of study medication will be summarized in the baseline table by treatment group and overall, using summary statistics in accordance with section 9.

No medication coding dictionary was used in this study. The investigators characterized concomitant medications by name.

10.7 Other Safety Measures

Vital signs (systolic and diastolic blood pressure) will be summarized using descriptive statistics by clinical visit (through week 16) and treatment group, as both observed value at the time point of interest and the change from baseline values.

11 Reporting Conventions

P-values ≥0.001 will be reported to 3 decimal places; p-values less than 0.001 will be reported as "<0.001". The mean, standard deviation, and any other statistics other than quantiles, will be reported to one decimal place greater than the original data. Quantiles, such as median, or minimum and maximum will use the same number of decimal places as the original data. Estimated parameters, not on the same scale as raw observations (e.g., regression coefficients) will be reported to 3 significant figures.

12 Summary of Changes to the Protocol and/or SAP

The changes from the protocol-specified definitions of aims, outcomes and statistical analytic approaches are outlined below. These changes reflect advances in our knowledge of digital ulcers and scleroderma since the design of the study in 2014-2015 that were not incorporated as protocol amendments, but were discussed during the formation of the Statistical Analysis Plan. These changes are documented herein and represent changes made prior to the database lock.

1. **Additional Secondary End points**

New information from FDA regulators expands the consideration of other digital ulcer end points described below. In addition, an additional measure of hand function has been added, based on categories of the HAQ-DI that are associated with hand function.

PROTOCOL:
N/A

SAP:
Section 4.2.2 Secondary Efficacy End points

- Proportion of participants with healing of all DUs at baseline by week 16. Baseline DUs are considered healed when classified as 'healed' and not 'active' or 'indeterminate' by week 16. **All** baseline ulcers must be healed for the participant to be classified as having all baseline ulcers healed. Note that this end point does not consider whether a participant develops new DUs during the course of the study.
- Proportion of participants with no DUs at week 16. This end point does not consider the

number of ulcers at baseline or during the course of the study; only the absence of 'active' and 'indeterminate' DUs at week 16.

- Change from baseline to Week 16 in the composite score for hand function. The sum of the individual scores for dressing, hygiene, and grip from the HAQ-DI will be calculated. These categories are associated with hand function.

2. **Reporting of Laboratory Data Abnormalities**

The reporting of the incidence of pre-specified laboratory data abnormalities was noted in the protocol; however, there no specific laboratory data abnormalities were defined and standard reporting of laboratory abnormalities that are adverse events are reported.

PROTOCOL:
Section 8.3.3. Safety
The safety evaluation of laboratory data will include:

- Incidence rates of pre-specified laboratory data abnormalities.

SAP:
None

15 References

1. Rubin DB. Multiple imputation for nonresponse in surveys: John Wiley & Sons; 2004.
2. Little RJ, Wang Y. Pattern-mixture models for multivariate incomplete data with covariates. Biometrics. 1996;52(1):98-111. Epub 1996/03/01. PubMed PMID: 8934587.

16 Listing of Tables, Listings and Figures

A separate document provides the mock tables, listings and figures.
