# Supplementary material for: A multicenter randomized, double-blind, placebo-controlled pilot study to assess the efficacy and safety of riociguat in systemic sclerosis-associated digital ulcers
Source: Arthritis Res Ther. 2019 Sep 3;21:202. doi: 10.1186/s13075-019-1979-7 (PMC6724329; doi:10.1186/s13075-019-1979-7)
Supplement: Supplementary file 3 — Comparison of the statsitical change in biomarkers levels from baseline between healthy controls and all patients. (DOCX 23 kb) [file 13075_2019_1979_MOESM3_ESM.docx]

**Supplementary material**

**Plasma biomarkers in healthy controls and patients at baseline (week 0). Data presented as Mean (SD).**

| **Biomarkers** | **Controls**  **(n = 17)** | **All Patients**  **(n = 17)** | **Placebo**  **(n=8)** | **Riociguat**  **(n=9)** | **P-value**  **Control vs. All Patients^1^** | **P-value**  **Placebo vs. Riociguat^1^** |
| --- | --- | --- | --- | --- | --- | --- |
| **cGMP (µM)** | 84.0  (88.5) | 137.8  (57.6) | 162.4  (43.4) | 116.0  (62.1) | **0.04** | 0.10 |
| **CXCL4 (ng/ml)** | 952.2 (315.1) | 1009.0  (589.5) | 1085.1  (722.6) | 941.5  (476.6) | 0.66 | 0.61 |
| **sE-Selectin (ng/ml)** | 34.2  (15.8) | 46.1  (21.6) | 45.5  (13.6) | 46.7  (27.7) | **0.03** | 0.54 |
| **VEGF (pg/ml)** | 113.6  (62.7) | 99.9  (23.3) | 91.3  (98.9) | 107.5  (98.6) | 0.22 | 0.89 |
| **sFLT1 (pg/ml)** | 1450.9  (2151.2) | 1916.1  (3146.1) | N=7  1330.3  (1145.4) | N=8  2428.7  (4245.9) | 0.35 | 0.96 |
| **tPA (ng/ml)** | 3.7  (4.0) | 4.6  (3.9) | 3.3  (1.4) | 5.7  (5.0) | 0.23 | 0.21 |
| **bFGF (pg/ml)** | 1.6  (0.9) | 2.6  (3.2) | 1.7  (0.9) | 3.4  (4.2) | 0.29 | 0.17 |
| **sICAM1 (ng/ml)** | 273.1  (86.8) | 505.8  (178.9) | 460.3  (91.6) | 546.3  (229.6) | **0.0001** | 0.34 |
| **VCAM1 (ng/ml)** | 110.2  (25.4) | 133.9  (43.54) | 118.5  (33.6) | 147.6  (48.5) | 0.06 | 0.18 |
| **PINP (pg/ml)** | 63.3  (79.1) | 23.3  (21.0) | 27.6  (25.7) | 19.5  (16.5) | 0.06 | 0.44 |
| **MMP12 (ng/ml)** | 0.69  (1.1) | 1.4  (2.5) | 0.57  (0.9) | 2.2  (3.2) | 0.29 | **0.046** |
| **Endostatin (ng/ml)** | 826.2  (340.3) | 1104.7  (485.2) | 1006.0  (580.7) | 1192.4  (396.8) | 0.06 | 0.45 |

^1^ by ANCOVA
